# Systematic Examination of Gene Expression and Proteomic Evidence Across Tissues Supports the Role of Mitochondrial Dysregulation in ME/CFS

**DOI:** 10.3390/ijms27041997

**Published:** 2026-02-19

**Authors:** Gregory R. Keele, Mike Enger, Quinn Barnette, Roman Ruiz-Esparza, Manuel Alvarado, Ravi Mathur, Jeran K. Stratford, Stephanie N. Giamberardino, Linda Morris Brown, Bradley T. Webb, Megan Ulmer Carnes

**Affiliations:** 1 RTI International, 3040 E Cornwallis Rd, Research Triangle Park, NC 27709, USA; gkeele@rti.org (G.R.K.); menger@rti.org (M.E.); qbarnette@rti.org (Q.B.); rruizesparza@rti.org (R.R.-E.); manuelalvarado@rti.org (M.A.); rmathur@rti.org (R.M.); jstratford@rti.org (J.K.S.); sgiamberardino@rti.org (S.N.G.); lindabrown@rti.org (L.M.B.); 2 Department of Population Health Sciences, Geisinger, Danville, PA 17822, USA; btwebb1@geisinger.edu; 3 Center for Substance Use Research and Education, Geisinger, Danville, PA 17822, USA; 4 Department of Psychiatry, Virginia Institute for Psychiatric and Behavioral Genetics, Virginia Commonwealth University, Richmond, VA 23298, USA

**Keywords:** myalgic encephalomyelitis, chronic fatigue syndrome, infection-associated chronic illness (IACI), gene expression, proteomics, drug repurposing, mapMECFS, data repository

## Abstract

Myalgic encephalomyelitis/chronic fatigue syndrome (ME/CFS) is a chronic, multisystem disease characterized by post-exertional malaise and persistent fatigue. The cause of ME/CFS is not well understood, and there are no established biomarkers or FDA-approved pharmacotherapies. The clinical heterogeneity of ME/CFS presents challenges to diagnosis and treatment and necessitates collaborative efforts to generate robust findings. This study leveraged gene and protein expression data from the mapMECFS data repository and the DecodeME Genome-Wide Association Study (GWAS) to assess consistent gene signatures across studies. The mitochondrial genes MT-RNR1 and MT-RNR2 exhibited lower expression in ME/CFS cases in two studies. Combining this with increased expression of mitochondrial genes in platelets from another study, this supports mitochondrial dysregulation as having a role in ME/CFS. Furthermore, ME/CFS-associated genes were mapped to compounds in drug databases as possible treatments for further investigation. In muscle gene expression data, 107 approved compounds target 26 genes with functions relevant to mitochondrial support and immunomodulators. From the DecodeME GWAS, 83 approved compounds target 24 genes with functions related to energy metabolism and mitochondrial function. Though little consistency in specific genes was observed across studies, which highlights the need for larger studies, mitochondrial dysfunction in ME/CFS cases was evident across studies.

## 1. Introduction

Myalgic encephalomyelitis/chronic fatigue syndrome (ME/CFS) is a complex, multisystem disease that affects millions of people worldwide [1]. ME/CFS is characterized by persistent and unexplained fatigue, post-exertional malaise (PEM), cognitive impairment, pain, and immune dysregulation [2]. Although these represent the hallmark symptoms of ME/CFS, the disease presents with a highly heterogeneous clinical profile, with symptoms and disease progression varying substantially across individuals [3]. The etiology and pathophysiology of ME/CFS are still poorly understood, and there are no specific diagnostic tests or effective treatments currently available [2]. This unmet clinical need and disease heterogeneity present significant challenges to traditional drug discovery for ME/CFS.

Identifying biomarkers and molecular signatures of ME/CFS could enable earlier and more accurate diagnosis, reduce reliance on subjective symptom reporting, and help distinguish ME/CFS from overlapping conditions. Additionally, molecular signatures may provide insights into the underlying etiology and risk factors of the disease and reveal biological subtypes, allowing researchers to stratify patients for clinical trials and tailor therapies to underlying pathophysiology. To identify biomarkers and molecular signatures of ME/CFS, researchers have profiled multiple omics data types, including transcriptomics and proteomics [4,5,6,7]. The extent of replication across studies, the consistency of results, and whether any discrepancies can be attributed solely to study design are currently unknown. Thus, there is a need to systemically reexamine and compare available data.

Genome-Wide Association Studies (GWAS) have also attempted to link genetic variants, along with their associated genes and proteins, to ME/CFS with the goal of identifying new biomarkers. While early studies were underpowered, the recent DecodeME GWAS represented a cohort of 15,579 ME/CFS cases [8]. Incorporating genomic signals from well-powered GWAS like DecodeME with other omics data resources represents another opportunity to detect consistent biological patterns across ME/CFS studies.

Because omics data can provide insights into the underlying mechanisms of the disease and reveal potential therapeutic targets, they also represent a potential resource for drug repurposing analyses [9] to identify existing compounds that may be effective for treating ME/CFS and its associated symptoms [10]. Drug repurposing has been successfully applied in various fields for decades. For example, zidovudine (AZT) was originally synthesized and tested as an anti-cancer drug in the late 1960s but was later repurposed and approved as the first anti-HIV drug in 1987 [11]. Bringing a novel drug to market is estimated to take over a decade and cost several billion dollars, making drug repurposing an appealing, cost-effective alternative [12,13,14]. Advances in computational methods and the growing availability of open-access biomedical data have supported this by creating a favorable environment for identifying new applications for existing compounds [15]. Data repositories provide researchers with centralized platforms where high-dimensional datasets can be accessed, reused, and integrated across studies. By providing access to previously published data, open data repositories allow researchers to derive new insights without the need to generate new data.

Recognizing the need for a centralized resource to facilitate data reuse and integration across ME/CFS studies, the mapMECFS data repository was created to collate ME/CFS study data [16]. The mapMECFS houses a range of data types to support ME/CFS research including molecular data (e.g., gene expression, DNA methylation, microRNA, proteomics, metabolomics, and cytokine profiles) and clinical and phenotypic data (e.g., demographic information, patient-reported outcome measures, and physical and cognitive assessments) on ME/CFS cases and controls.

In this study, we (1) conduct a comprehensive review of available ME/CFS gene expression and proteomic studies to evaluate cross-study findings, (2) incorporate results from the DecodeME GWAS with the goal of identifying consistent signals, and (3) attempt to identify potential therapeutic candidates via drug repurposing. Recognizing the variability in analytic approaches and experimental design across studies, we applied a single, standardized bioinformatics pipeline across gene expression and proteomic datasets to maximize cross-study comparability.

## 2. Results

### 2.1. ME/CFS Differential Gene Expression and Proteomic Studies—Current State of the Field

We surveyed both mapMECFS and the broader ME/CFS literature for studies that investigated gene expression and protein profiles between cases and controls and found six studies representing 11 gene expression and 10 proteomics datasets (Supplementary Table S1). After filtering by inclusion criteria (e.g., >10 cases and no gene array data for gene expression), four bulk tissue gene expression datasets, one single-cell gene expression dataset, and four proteomic datasets from ME/CFS case/control cohorts were retained for analysis (Table 1). Expression data were generated from multiple tissues or cell types including PBMC [4,17,18], monocytes derived from PBMC [19], and muscle [4]. For protein expression, the datasets were generated from plasma [4,20], extracellular vesicles derived from plasma [21], and cerebral spinal fluid (CSF) [4]. The sample sizes for both expression and proteomics studies ranged from 21 to 67. See Table 1 for more details on these studies.

When the proportion of null effects (p_0_) was examined, the values ranged from 0.709 to 1 with three studies being indistinguishable from a null distribution. A variety of quality control, filtering, and analysis methods were used for the results reported in the primary publications. To reduce heterogeneity, we reanalyzed all datasets using a common analysis workflow (Supplementary Figure S1) (see Methods). We note that these data represent distinct studies with varying scientific questions and experimental conditions, and thus we adjusted our analysis for a specific dataset based on its experimental design (see study-specific analyses in Supplementary File S1 for full analysis code and results).

**Figure 1:**
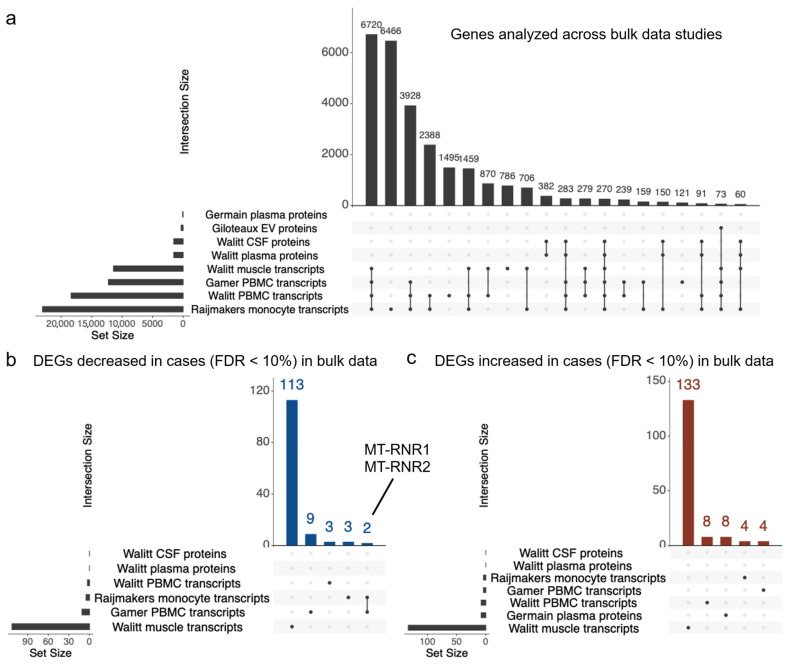
Comparison of genes and differential expression results across eight datasets from bulk tissue samples. (**a**) Overlapping genes analyzed across the eight datasets. Counts of genes with (**b**) decreased and (**c**) increased expression in ME/CFS cases (FDR < 10%) across the eight datasets. Horizontal bars represent the number of genes in each dataset. Vertical bars represent the number of DEGs observed across datasets. Dots and line segments indicate datasets for each DEG set. Genes detected across multiple datasets are highlighted.

Across all data (bulk gene expression and proteomics), a total of 27,374 genes were observed with 18,476 being observed in at least two datasets. For gene expression (e.g., transcripts), 6720 genes were observed and tested across all four bulk gene expression datasets (Figure 1a). The number of proteins quantified across the four protein datasets ranged from 301 to 1281 for the Giloteaux et al. [21] plasma data and Walitt et al. [4] plasma and CSF data, respectively. This variation in number of proteins likely stems, at least in part, from the specific proteomics assays and processing of the data. No genes were observed and analyzed across all datasets.

**Figure 2:**
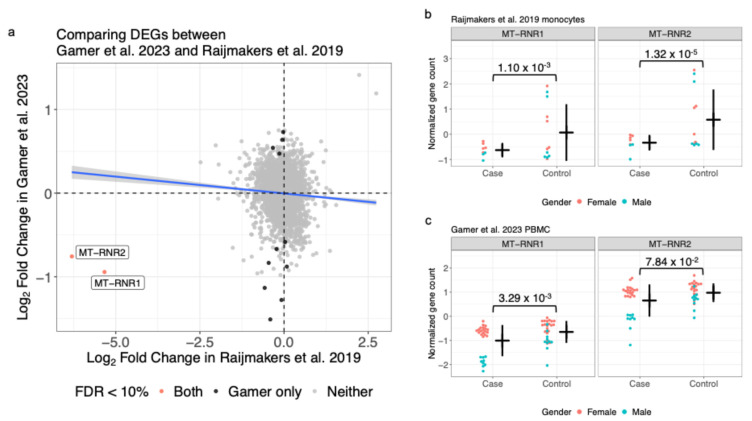
Consistent signal between two studies highlights mitochondrial ribosomal genes. (**a**) Comparison of log_2_ fold change between ME/CFS cases and controls from Gamer et al. [18] and Raijmakers et al. [19]. Blue best fit regression line +/− SE interval comparing log_2_ fold changes included for reference. Vertical and horizontal dashed lines at 0 also included for reference. Expression levels for MT-RNR1 and MT-RNR2 from (**b**) Raijmakers et al. [19] and (**c**) Gamer et al. [18] with mean +/− SD bars for cases and controls. FDR-adjusted *p*-values comparing cases and controls included.

Using an FDR threshold of 10%, bulk expression studies yielded between 9 and 246 significant genes (Table 1). We observed two DEGs that were consistent across multiple studies (Figure 1b). MT-RNR1 and MT-RNR2 encode for the 12S and 16S rRNA in the mitochondrial genome and showed lower expression in ME/CFS cases compared to controls across two studies, notably in PBMC [18] and monocytes derived from PBMC [19] (Figure 2). Both studies showed sex differences between cases and controls with the effect stronger in males. Using the lenient FDR < 30% threshold expands consistent DEGs by two genes: FABP5 had decreased expression in PBMC [18] and muscle [4] from ME/CFS cases and CDNF had increased protein expression in plasma [20] and transcript expression in muscle [4] from ME/CFS cases (Supplementary Figure S2). The full differential expression results are available in Supplementary File S2 as well as from mapMECFS (https://mapmecfs.org/group/keele-enger-systematic-examination-of-gene-expression-and-proteomics).

In contrast, there were no overlapping results across the proteomic datasets. Most (three of four) of the protein datasets showed no significant genes (FDR < 10%). One study, Germain et al. [20], had eight genes that met this threshold (Table 1). None of these overlapped those identified in the gene expression studies.

### 2.2. Differential Gene Expression Analysis of scRNA-Seq Pseudobulk Data

**Figure 3:**
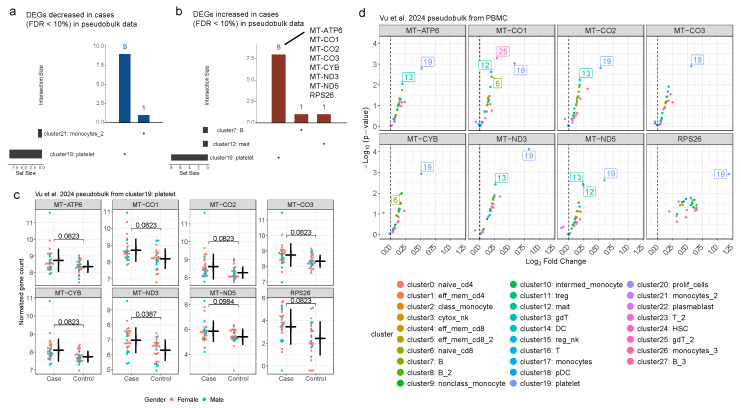
Increased mitochondrial gene expression in platelets. Counts of genes with (**a**) decreased and (**b**) increased expression in ME/CFS cases (FDR < 10%) across the pseudobulk cluster datasets from Vu et al. [5]. Genes with increased expression in platelets highlighted, which represent mostly mitochondrial genes. Horizontal bars represent the number of genes in each cluster. Vertical bars represent the number of DEGs observed across clusters. Dots and line segments indicate clusters for each DEG set. (**c**) Expression levels for eight genes with significantly increased expression in platelets (FDR < 10%) with mean +/- SD bars for cases and controls. FDR-adjusted *p*-values (Padj) comparing cases and controls included. (**d**) Comparison of statistical significance [-Log_10_(*p*-value)] to difference (log_2_ fold change) between ME/CFS cases and controls for genes with significantly increased expression in platelets (FDR < 10%) across clusters. Clusters with *p*-value < 0.01 highlighted with cluster numbers matching legend. Vertical dashed line at 0 included for reference.

Next, we reanalyzed pseudobulk data derived from PBMC samples from 30 ME/CFS cases and 28 controls originally generated and presented by Vu et al. [5]. Consistent with the bulk data from different tissues, most genes were not observed in all clusters (cell types) (Supplementary Figure S3). Based on a lenient threshold (FDR < 30%), no DEGs were detected across multiple clusters (Figure 3a,b, Supplementary Figure S4). Notably, of the 28 clusters, platelets (cluster 19) had the highest number of DEGs with 17 and 282 genes at an FDR of 10% and 30%, respectively (Supplementary File S2). Eight of the 17 are upregulated in ME/CFS cases, and 7 (88%) of those represent genes encoded on the mitochondrial genome, similar to MT-RNR1 and MT-RNR2 identified in the bulk gene expression data. Note that MT-RNR1 and MT-RNR2 specifically were not available in the scRNA-seq generated pseudobulk data. Looking more closely at these genes in the other clusters, cases consistently showed increased expression for these genes when compared to controls. This is consistent with other studies reporting elevated mitochondrial gene expression [22] and hyperactivation of platelets [23] in ME/CFS cases.

### 2.3. Gene-Level Analysis of DecodeME GWAS

DecodeME [8] reported 29 tier 1 genes (Supplementary Figure S5a; Supplementary File S2), representing genes that fell within a genome-wide significant interval and had a posterior probability of colocalization with an eQTL from GTEx [24]. We also defined significantly associated genes based on a 10% FDR threshold with the MAGMA [25] gene-level test, resulting in 178 genes, 14 of which were also in the tier 1 genes (Supplementary Figure S5b; Supplementary File S2). None of the 193 genes from across the tier 1 or MAGMA 10% FDR sets were detected as DEGs.

### 2.4. Drug Repurposing Analysis

**Figure 4:**
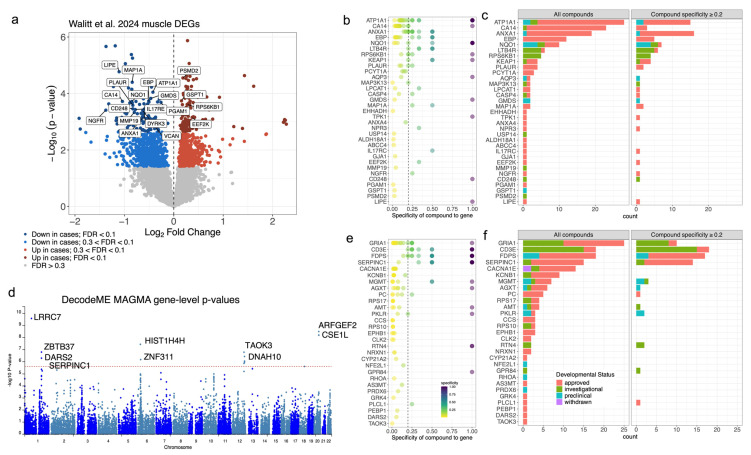
ME/CFS associated genes provide potential for therapeutic compound identification. (**a**) Volcano plot of DEGs in muscle tissue from Walitt et al. [4]. Vertical dashed line at origin included for reference. The top 20 genes with targeting compounds in at least one of four drug databases are highlighted. Genes prioritized based on both statistical significance and mapping to compounds in the databases. (**b**) The specificity of compounds targeting the muscle DEGs (FDR < 10%) in the drug databases. Vertical dashed line at 0.2 specificity included for reference. (**c**) The number of compounds that target each of the muscle DEGs (FDR < 10%) in the drug databases, colored according to the development status of compound, based on either no specificity filter (left) and filtering to compounds with specificity > 0.2. (**d**) Manhattan plot of MAGMA gene-level *p*-values from the DecodeME GWAS [8]. Ten of the top genes indicated with labels. Red horizontal dashed line representing Bonferroni threshold included for reference. (**e**) The specificity of compounds targeting each of the GWAS genes (FDR < 10%) in the drug databases. Vertical dashed line at 0.2 specificity included for reference. (**f**) The number of compounds that target each of the GWAS genes (FDR < 10%) in the drug databases, colored according to the development status of compound, based on either no specificity filter (left) and filtering to compounds with specificity > 0.2.

Next, the gene-centric drug repurposing tool, Realomics [26], was used on the sets of significant genes for each dataset to identify compounds with therapeutic potential for ME/CFS. The highest number of compounds were identified in the Walitt et al. [4] muscle tissue and DecodeME MAGMA-based results [8]. For the 246 DEGs identified in the Walitt et al. [4] muscle bulk gene expression data, 33 genes have at least one corresponding targeting compound in one of the databases (Figure 4a–c, Supplementary File S3). The list of candidate therapeutics represents 139 compounds overall, of which 107 are clinically approved agents, and of those, 76 have specificity ≥ 0.2 (Table 1). These compounds encompass a diverse range of treatments targeting energy metabolism, antiviral, anti-inflammatory, immunomodulatory, neurological, hormonal, and cardiac functions. Of particular interest are those that modulate biological processes thought to be dysregulated in ME/CFS, including (1) mitochondrial [27,28,29] and metabolic support [30,31] (e.g., thiamine); (2) immunomodulators [32,33,34] (e.g., dimethyl fumarate/diroximel fumarate and ruxolitinib); and (3) neuromodulator agents [35,36]: opipramol. This list highlights only a subset of the candidate therapeutics identified. However, these results should be interpreted with caution because they have not been evaluated for the treatment of ME/CFS and may have detrimental side effects.

Using the bulk gene expression and scRNA-seq data generated from PBMCs identified only three additional clinically approved compounds with a specificity ≥ 0.2, which did not overlap compounds identified in other sample types. The proteomic studies did not identify any additional compounds with these criteria (Table 1, Supplementary File S3).

Using gene-based analyses from DecodeME GWAS genes [8] (Figure 4d–f), 29 of the 178 genes with an FDR < 10% were associated with at least one compound in one of the databases, including 22 that were targeted by clinically approved compounds. A number of these genes are involved in energy metabolism, including Pyruvate Carboxylase (*PC*), which replenishes intermediates of the TCA cycle, and *DARS2*, which is essential for protein synthesis within mitochondria. Other implicated biological pathways are neurons and neuro-immunity (e.g., *CACNA1E*, *GRIA1*, *NRXN1*, *KCNB1*) and cellular maintenance and regulation (e.g., *CLK2*, *MGMT*, *PEBP1*). In total, there were 137 candidate therapeutics overall with 82 representing clinically approved agents and 31 with a specificity ≥ 0.2. When looking at the DecodeME GWAS tier 1 gene list, two additional compounds targeting the *PTGIS* gene and one additional compound targeting *CA10* were identified, albeit each had a specificity of 0.25 or lower (Supplementary File S3). Interestingly, there was overlap between the GWAS and muscle gene expression candidate therapeutics, with two small molecules being identified in both (zoledronic acid: CHEMBL924 and incadronic acid: CHEMBL53950; Supplementary Figure S6). It is important to note that the direction of the effect is not accounted for when identifying these target compounds. Further investigation would be required to evaluate the therapeutic potential of identified compounds.

## 3. Discussion

The mapMECFS repository [16] was used to survey available data from studies that profiled gene and protein expression in ME/CFS to look for consistent evidence across studies and identify genes that could reveal insights about the fundamental biology and etiology of ME/CFS. Genes with consistent or converging evidence represent new avenues for potential treatment because existing gene–drug pairs can be identified from drug databases.

Gene and protein expression analysis have been used to compare the responses of individuals with ME/CFS and healthy controls to exercise stimuli (via cardiopulmonary exercise tests [CPET]) [5,18,21] via study designs with repeated measures at different timepoints relative to stimuli (e.g., before and after CPET). The data collected by these studies can be analyzed in various ways depending on the scientific question. Variation in study design, scientific questions, and technical features, combined with relatively modest sample sizes (*n* < 50), represent a significant challenge to detecting converging evidence across studies. Even with these caveats, this study has shown evidence of mitochondrial dysregulation in ME/CFS across studies and identified candidate drugs for repurposing using existing results.

The overlap of analyzed gene transcripts and proteins across studies is generally limited. This is not surprising given that it is technically challenging to quantify thousands of proteins in a sample. Though proteomics technology is making great strides, generally far fewer proteins are quantified in a proteomics study (hundreds to a few thousand) than a gene expression study (15 to 20 thousand). This implicitly results in many genes being observed in only a subset of the datasets. This has likely contributed to the lack of consistent signal observed within the field, along with heterogeneity in study design and methodology.

Ultimately, much larger omics studies of ME/CFS are needed to advance the field in terms of identifying reliable molecular signatures, illuminating the underlying etiology of the disorder, and ultimately developing therapeutic treatments. This is particularly important given the heterogeneity of ME/CFS, which potentially reflects different etiologies, subtypes, disease stages, or underlying mechanisms. Genetic studies of ME/CFS [37,38,39,40,41,42] have similarly been hampered by underpowered sample sizes (in the context of genome-wide association studies for a highly complex phenotype) to produce reliable genetic signatures. Large-scale biobanks, such as UK Biobank [43] and All of Us [44], continue to expand with data types relevant to ME/CFS, including omics (e.g., metabolomics and proteomics) and electronic health records. These powerful resources contain information on participants that span the heterogeneity of ME/CFS and are now beginning to be leveraged for studies on how genetics and molecular intermediates (i.e., biomarkers) influence ME/CFS [45].

Our study supports the hypothesis of mitochondrial dysregulation in individuals with ME/CFS. We note that the direction of the effect for mitochondrial gene differential expression differed between bulk tissue and scRNA clusters, with MT-RNR1 and MT-RNR2 expression decreased in ME/CFS cases compared to increased expression in platelets for the seven mitochondrial genes (MT-ATP6, MT-CO1, MT-CO2, MT-CO3, MT-CYB, MT-ND3, and MT-ND5). Nevertheless, these results implicate mitochondrial dysregulation as a potentially complex feature of ME/CFS observed across studies, which is consistent with other studies [27,28].

The findings for MT-RNR1 and MT-RNR2, which we reproduced from the initial report by Raijmakers et al. [19], are particularly robust because they were replicated in the PBMC data from Gamer et al. [18]. MT-RNR1 and MT-RNR2 were not originally reported by Gamer et al. [18], which was more focused on findings related to PEM. MT-RNR1 and MT-RNR2 are both genes found in the non-nuclear mitochondrial genome and encode the mitochondrial ribosomal RNA 12S and 16S subunits, respectively. The peptides encoded by MT-RNR1 and MT-RNR2 are also known as MOTS-C (mitochondrial open reading frame of the 12S rRNA-c) and humanin, respectively. There are numerous preclinical studies of MOTS-C for a wide range of proposed applications [46]. Notably in the context of ME/CFS, MOTS-C is upregulated in response to exercise [47], considered an exercise mimetic [48], and a potential performance enhancing drug [49]. However, it should be noted that the peptide MOTS-C is not FDA approved, there are no active MOTS-C clinical trials, and safety risks have been documented [50,51]. Similar to MOTS-C, humanin is upregulated in response to exercise [47] and has been investigated in a wide variety of preclinical studies in which mitochondrial dysfunction and energy regulation have been implicated including in Alzheimer’s disease and diabetes. Because preclinical studies involve treatment with these endogenous bio-identical peptides, human clinical trials using MOTS-C and humanin are likely feasible. This could potentially bypass the need to develop small molecules to modulate MT-RNR1 and MT-RNR2 activity or abundance in mitochondria. We note that modified peptides are a rapidly growing area of therapeutics, e.g., GLP-1 receptor agonists for obesity and diabetes [52].

Signatures of mitochondrial dysfunction have been noted in other infection-associated chronic illnesses (IACIs) [53], such as post-acute sequelae of COVID (PASC), i.e., long COVID, post-treatment Lyme disease syndrome (PTLDS), chronic Q fever fatigue syndrome, and post viral fatigue syndromes [54]. These signatures include elevated biomarkers of oxidative stress and mitochondrial damage in long COVID [55,56] and PTLDS [57]. These debilitating chronic disorders share an infection-associated origin as well as exhibit similar clinical features (e.g., fatigue and cognitive dysfunction). Further understanding of how mitochondrial dysfunction contributes to ME/CFS could also prove meaningful for other IACIs.

Another promising but underdeveloped avenue for ME/CFS research is drug repurposing, which is the process of finding new therapeutic uses for existing drugs that have already passed regulatory approval. Drug repurposing can accelerate the drug development process for ME/CFS and reduce the cost and risk of failure because the safety and pharmacokinetics of the repurposed drugs are already known. Compounds that target ME/CFS-associated genes that are identified through drug repurposing could also represent new information based on their molecular targets and mechanisms of action, which, along with the target genes, could provide insights into the pathophysiology of ME/CFS and further validate potential biomarkers.

Here we have conducted an analysis to identify potential new therapeutics for the treatment of ME/CFS. Across all studies, we identified 201 FDA-approved candidate compounds, of which 89 showed specificity of 0.2 or greater, revealing several potential therapeutics of interest. To date, ME/CFS drug repurposing investigations are limited. Jeffrey et al. [10] identified differentially expressed gene modules in PBMC which they then queried in PharmGKB [58]. Broadly, their approach is similar to ours, although we frame our analysis around single genes rather than pathways and we query four drug databases rather than one. Despite the tissues differing between studies (PBMC compared to muscle), we do observe some system-level overlap, most notably, compounds that affect immune and mitochondrial function.

Although it is not feasible to systemically assess or discuss the plausibility of the full catalog of results here, we highlight selected compounds that appear to have additional evidence supporting their relationship to ME/CFS biology, symptoms, or features. First, thiamine (Vitamin B1) is associated with the gene TPK1 (DEG p_adj_ = 0.093) and at high doses has shown promise in improving fatigue and cognitive symptoms [59], likely by boosting mitochondrial function [60]. Mitochondrial and metabolic support in ME/CFS through various supplements has previously been proposed [61,62]. We also identified 14 compounds associated with the gene ATP1A1 (DEG p_adj_ = 0.029), which encodes the alpha-1 subunit a Na+/K+-ATPase pump and plays a vital role in regulating blood pressure by controlling sodium and potassium ion movement. One notable compound associated with ATP1A1 is artemether, an antimalarial medication. Artemether is an artemisinin derivative, and another drug in this class, artesunate, has been investigated as a ME/CFS treatment [63]. Dimethyl fumarate and diroximel fumarate target the product of the KEAP1 gene (Kelch-like ECH-associated protein 1; DEG p_adj_ = 0.071) and have immunomodulatory and antioxidant effects that could address neuroinflammation and oxidative stress. Two compounds target the PLAUR gene (DEG p_adj_ = 0.039) including alteplase and ruxolitinib. Ruxolitinib is a JAK1/2 inhibitor, has anti-inflammatory effects, and has been proposed for ME/CFS-related immune dysregulation [64]. Five compounds target the gene EBP (DEG p_adj_ = 0.039) including buflomedil, clomiphene, opipramol, triparanol, and trifluperidol. Opipramol is a neuropsychiatric and neuromodulatory treatment which has anxiolytic and antidepressant properties that could benefit ME/CFS-related anxiety, sleep disturbances, and mood symptoms.

Although some of the identified compounds appear to be plausible treatments for ME/CFS, we emphasize that these findings are preliminary and should be interpreted with caution. Further research is necessary to ascertain the viability and efficacy of these options and would require rigorous clinical trials to determine their effectiveness in individuals with ME/CFS. Nevertheless, drug repurposing represents an important initial step towards identifying potential interventions to treat ME/CFS.

This study aimed to collect accessible gene expression and proteomic data for ME/CFS, process through a common pipeline, and search for converging evidence, but there are some notable limitations. First, gene expression data were limited to RNA-seq for technical reasons, and older array-based results were excluded. Second, FDR was used, except for with the DecodeME tier 1 results, instead of family-wise multiple testing correction such as Bonferroni. Although this strategy is statistically justified particularly for omics data with non-independent tests, some of the reported results will represent false positives. Therefore, some caution is warranted when interpreting the results. Third, metabolomic and cytokine data were not considered and should be investigated in future studies. Finally, although many candidate gene–drug pairs were identified, drug repurposing for ME/CFS faces several challenges, such as the lack of validated animal models or model systems for testing and screening potential drugs, and the difficulty of accessing and integrating heterogeneous and dispersed data sources.

## 4. Materials and Methods

### 4.1. Gene Expression and Proteomic Data Harmonization and Analysis Framework

First, we sought to identify, ingest, and harmonize available gene and protein expression datasets from ME/CFS case/control studies to enable integration into drug repurposing tools and databases to aid in therapeutic discovery. The search started with the data available in the mapMECFS data repository [16] (https://mapmecfs.org (accessed on 9 July 2025)). The mapMECFS is the largest repository of ME/CFS-specific data and enables data findability and reanalysis of published ME/CFS data and the potential to increase the effective sample size by utilizing multiple studies. The query was expanded to other potential sources of data, including systematic querying of PubMed and Gene Expression Omnibus [65] (Supplementary Table S1). The selection of gene expression studies was constrained to those that used RNA-seq based assays, excluding studies without available gene count data [66] or that used older microarray assay [10,67,68], to maximize the harmonization of analysis and results across studies. Only studies with 10 or more ME/CFS cases and that used large-scale assays were included (i.e., small studies and those focused on a small number of candidate genes were excluded [69,70]). A small number of proteomic studies were excluded for not having processed data available [7,71].

To improve comparability, we defined a consistent statistical framework to be used across the studies (Supplementary Figure S1). For gene expression studies, publicly available raw gene count data were obtained and used as input for differential gene expression analysis via the DESeq2 R package [72] (v1.46.0) to test for genes with expression levels that differed between cases and controls. Proteomics studies employed the aptamer-based SomaScan assay [73] and tandem mass tag mass spectrometry [74]. Differential protein expression analysis was performed by first log_10_-transforming protein intensities, which were then individually tested for differences between cases and controls through analysis of variance.

For studies with repeated measures, we kept only baseline measures (i.e., before PEM induction or an exercise challenge) to improve the comparability with case-control studies. Most of the studies included both females and males, which we adjusted for in subsequent analysis. We used two significance thresholds, including a 10% false discovery rate (FDR) to define higher confidence ME/CFS-associated genes and a lenient 30% FDR threshold coupled with detection across multiple studies. This approach would potentially allow us to identify additional genes with some support across multiple studies. We also estimated the proportion of null effects for each dataset (p_0_), representing the overall proportion of genes or proteins in the data with no difference between cases and controls, as a single summary statistic of the extent of signal in each dataset. This is necessary to evaluate whether the summary statistics, in aggregate from a given study, contain a mixture of true and null effects. Underpowered studies may not yield any significant results after applying family-wise multiple testing correction but a p_0_ < 1 supports that the study is appropriate for FDR analysis. Alternatively, if p_0_~1, this implies that the results are unlikely to be different from a null distribution and not merely underpowered.

Differing from the other RNA-seq-based studies, the data from Vu et al. [5] represent single-cell RNA-seq (scRNA-seq) from peripheral blood mononuclear cell (PBMC) samples from ME/CFS cases and controls. Seurat, an R package for analyzing single-cell data [75], was used by Vu et al. [5] to (1) perform cluster analysis on the single-cell-level data for dimension reduction, resulting in 29 clusters, (2) define marker genes to annotate clusters with specific cell types, and then (3) summarize as pseudobulk quantitative data. Using the pseudobulk data from Vu et al. [5], the 29 clusters were filtered to remove any with fewer than 10 cases, resulting in the exclusion of one cluster with two individuals. Using the remaining 28 clusters, we performed differential gene expression using the same pipeline as the bulk data (as described above).

### 4.2. Gene-Level Analysis of DecodeME GWAS Summary Statistics

We obtained the summary statistics from the DecodeME GWAS [8], specifically based on 15,579 ME/CFS cases and 259,909 UK Biobank population controls with European ancestry. We used the Functional Mapping and Annotation of Genome-Wide Association Studies (FUMA) [76] (v1.5.2) web portal (https://fuma.ctglab.nl/) to perform a gene-level association analysis using Multi-Marker Analysis of Genomic Annotation (MAGMA) [25] (v1.08). A 10 Kbp window around each gene was used for assigning SNPs to genes. We used two definitions for ME/CFS-associated genes: (1) the tier 1 genes defined by the DecodeME GWAS original manuscript [8], which was based on co-localization analysis with GTEx [24] eQTL and (2) a 10% FDR threshold for the MAGMA results.

### 4.3. Drug Repurposing Analysis with Realomics

For the drug repurposing analysis, we used the Realomics tool [26]. Realomics accepts a user-supplied list of prioritized genes known to be associated with a phenotype and queries four drug databases (Pharos [77], Open Targets [78], Therapeutic Target Database [TTD] [79], and DrugBank [80]) to identify compounds known to target the priority genes. Briefly, we defined sets of ME/CFS-associated genes (mapped to GRCh38 Ensembl gene IDs) for each dataset using a 10% FDR threshold for gene or protein expression datasets and DecodeME MAGMA results, as well as DecodeME tier 1 genes. Each set of genes were input individually into Realomics [25], producing a dataset-specific set of compounds. Only compounds with ChEMBL IDs were retained as potential therapeutics of interest. We note that gene–compound annotations in databases represent a range of relationships, such as compounds that bind gene protein products. Here we do not attempt to filter gene–compound pairs based on relationship type because that level of information is highly variable across compounds and databases. Identified compounds were prioritized based on the number of ME/CFS-associated genes with which they were associated and their specificity to ME/CFS-associated genes. This specificity metric provides information about a compound’s potential for off-target effects with treatment. The relative specificity of a drug is defined as the number of target genes/total number of genes targeted by the drug. Determining a specificity threshold empirically is challenging, but the Realomics developers recommend a specificity filtering threshold of 0.2 as a starting point. As such, drugs with a relative specificity less than 0.2 were considered nonspecific because more than 80% of the gene targets were not considered ME/CFS-associated and thus less likely to be good repurposing candidates.

## 5. Conclusions

This study surveyed gene expression and proteomics studies, many of which are now available in the mapMECFS repository, to perform reanalysis under a standardized framework toward detecting consistent gene signatures across multiple studies. Although consistent gene signatures were limited at the level of individual genes (MT-RNR1 and MT-RNR2 had reduced expression in two studies), signs of mitochondrial dysregulation were observed more broadly. Drug repurposing analysis was performed on each study’s gene results, representing a potential avenue for discovering treatment options for ME/CFS. All analysis code and results data from all studies have been made available on mapMECFS (https://mapmecfs.org/group/keele-enger-systematic-examination-of-gene-expression-and-proteomics, accessed on 9 July 2025), which ensures reproducibility of the findings. This study demonstrates the value of these rich datasets as resources for secondary analyses.

## Figures and Tables

**Table 1:** Gene expression and proteomics datasets used in this study.

Data Type	Assay	Publication/ mapMECFS Reference	Tissue	Sample Size ^a^	Study Design	Null Proportion (p0) %	Number of Genes	DEGS: FDR < 10%	DEGs: FDR < 30%	Number of Approved Compounds ^c^
Gene Expression	scRNA-seq	Vu et al. 2024 [5]	PBMC	30/28	Two samples per subject. Prior to CPET and 24 h post.	63.7 ^b^–100	16,523	20	292	0 (maits) 0 (monocytes) 2 (platelets)
	Bulk RNA-seq	Van Booven et al. 2023 [17] Gamer et al. 2023 [18] https://mapmecfs.org/dataset/me-cfs-case-control-rna-seq-study-lubov-nathanson (accessed on 9 July 2025)	PBMC	33/34	A total of 38 females and 19 males. Prior to CPET, at maximal exertion, and 4 h post.	100	12,261	15	50	1
		Walitt et al. 2024 [4] https://mapmecfs.org/organization/nih-intramural (accessed on 9 July 2025)	PBMC	15/11	Males and females. Post-infectious cases.	99.4	18,369	11	19	0
			Muscle	12/13	Males and females. Post-infectious cases.	70.9	11,423	246	1379	55
		Raijmakers et al. 2019 [19] https://mapmecfs.org/dataset/me-cfs-and-qfs-case-control-rna-expression-study-gse130353 (accessed on 9 July 2025)	Monocytes from PBMC	11/10	Males and females.	95.7	23,066	9	22	0
Proteomics	SomaScan	Walitt et al. 2024 [4] https://mapmecfs.org/organization/nih-intramural (accessed on 9 July 2025)	Plasma	15/18	Males and females. Post-infectious cases.	99.5	1281	0	0	0
			CSF	15/18	Males and females. Post-infectious cases.	100	1281	0	0	0
		Germain et al. 2021 [20] https://mapmecfs.org/dataset/me-cfs-case-control-plasma-proteomics (accessed on 9 July 2025)	Plasma	20/20	All females.	74.6	4739	8	49	0
	Mass spectrometry	Giloteaux et al. 2023 [21] https://mapmecfs.org/dataset/dysregulation-of-ev-protein-cargo-in-me-cfs-cases-and-sedentary-ctrls-in-response-to-max-exercise (accessed on 9 July 2025)	Extracellular vesicles from plasma	18/17	All females. Prior to CPET and 15 min and 24 h post. TMT mass spectrometry.	100	301	0	0	0

^a^ Case/Control; ^b^ Platelets, see Supplementary File S1 for full cluster results; ^c^ Approved compounds with a specificity ≥ 0.2. Acronyms: peripheral blood mononuclear cells (PBMC), cardiopulmonary exercise test (CPET), cerebral spinal fluid (CSF), tandem mass tag (TMT). For a comprehensive list of data investigated, see Supplemental Table S1.

## Data Availability

The data presented in this study (analysis code, results, and links to the data from each input study) are available in mapMECFS at https://mapmecfs.org/group/about/keele-enger-systematic-examination-of-gene-expression-and-proteomics).

## Supplementary Materials

The following supporting information can be downloaded at: https://www.mdpi.com/article/10.3390/ijms27041997/s1. Reference [81] is cited in supplementary file (mapMECFS_DRUG_supplementary_materials.pdf)

## References

[1] Tschopp R., König R.S., Rejmer P., Paris D.H. (2023). Myalgic encephalomyelitis/chronic fatigue syndrome (ME/CFS): A preliminary survey among patients in Switzerland. Heliyon.

[2] Grach S.L., Seltzer J., Chon T.Y., Ganesh R. (2023). Diagnosis and Management of Myalgic Encephalomyelitis/Chronic Fatigue Syndrome. Mayo Clin. Proc..

[3] Unger E.R., Lin J.-M.S., Chen Y., Cornelius M.E., Helton B., Issa A.N., Bertolli J., Klimas N.G., Balbin E.G., Bateman L. (2024). Heterogeneity in Measures of Illness among Patients with Myalgic Encephalomyelitis/Chronic Fatigue Syndrome Is Not Explained by Clinical Practice: A Study in Seven U.S. Specialty Clinics. J. Clin. Med..

[4] Walitt B., Singh K., LaMunion S.R., Hallett M., Jacobson S., Chen K., Enose-Akahata Y., Apps R., Barb J.J., Bedard P. (2024). Deep phenotyping of post-infectious myalgic encephalomyelitis/chronic fatigue syndrome. Nat. Commun..

[5] Vu L.T., Ahmed F., Zhu H., Iu D.S.H., Fogarty E.A., Kwak Y., Chen W., Franconi C.J., Munn P.R., Tate A.E. (2024). Single-cell transcriptomics of the immune system in ME/CFS at baseline and following symptom provocation. Cell Rep. Med..

[6] Glass K.A., Germain A., Huang Y.V., Hanson M.R. (2023). Urine Metabolomics Exposes Anomalous Recovery after Maximal Exertion in Female ME/CFS Patients. Int. J. Mol. Sci..

[7] Milivojevic M., Che X., Bateman L., Cheng A., Garcia B.A., Hornig M., Huber M., Klimas N.G., Lee B., Lee H. (2020). Plasma proteomic profiling suggests an association between antigen driven clonal B cell expansion and ME/CFS. PLoS ONE.

[8] Boutin T., Bretherick A.D., Dibble J.J., Ewaoluwagbemiga E., Northwood E., Samms G.L., Vitart V., Almelid Ø., Genetics Delivery Team, Project and Cohort Delivery Team (2025). Initial findings from the DecodeME genome-wide association study of myalgic encephalomyelitis/chronic fatigue syndrome. medRxiv.

[9] Pulley J.M., Rhoads J.P., Jerome R.N., Challa A.P., Erreger K.B., Joly M.M., Lavieri R.R., Perry K.E., Zaleski N.M., Shirey-Rice J.K. (2020). Using What We Already Have: Uncovering New Drug Repurposing Strategies in Existing Omics Data. Annu. Rev. Pharmacol. Toxicol..

[10] Jeffrey M.G., Nathanson L., Aenlle K., Barnes Z.M., Baig M., Broderick G., Klimas N.G., Fletcher M.A., Craddock T.J.A. (2019). Treatment Avenues in Myalgic Encephalomyelitis/Chronic Fatigue Syndrome: A Split-gender Pharmacogenomic Study of Gene-expression Modules. Clin. Ther..

[11] Pushpakom S., Iorio F., Eyers P.A., Escott K.J., Hopper S., Wells A., Doig A., Guilliams T., Latimer J., McNamee C. (2019). Drug repurposing: Progress, challenges and recommendations. Nat. Rev. Drug Discov..

[12] DiMasi J.A., Grabowski H.G., Hansen R.W. (2016). Innovation in the pharmaceutical industry: New estimates of R&D costs. J. Health Econ..

[13] Pinzi L., Rastelli G. (2023). Trends and Applications in Computationally Driven Drug Repurposing. Int. J. Mol. Sci..

[14] Parvathaneni V., Kulkarni N.S., Muth A., Gupta V. (2019). Drug repurposing: A promising tool to accelerate the drug discovery process. Drug Discov. Today.

[15] Masoudi-Sobhanzadeh Y., Omidi Y., Amanlou M., Masoudi-Nejad A. (2020). Drug databases and their contributions to drug repurposing. Genomics.

[16] Mathur R., Carnes M.U., Harding A., Moore A., Thomas I., Giarrocco A., Long M., Underwood M., Townsend C., Ruiz-Esparza R. (2021). mapMECFS: A portal to enhance data discovery across biological disciplines and collaborative sites. J. Transl. Med..

[17] Van Booven D.J., Gamer J., Joseph A., Perez M., Zarnowski O., Pandya M., Collado F., Klimas N., Oltra E., Nathanson L. (2023). Stress-Induced Transcriptomic Changes in Females with Myalgic Encephalomyelitis/Chronic Fatigue Syndrome Reveal Disrupted Immune Signatures. Int. J. Mol. Sci..

[18] Gamer J., Van Booven D.J., Zarnowski O., Arango S., Elias M., Kurian A., Joseph A., Perez M., Collado F., Klimas N. (2023). Sex-Dependent Transcriptional Changes in Response to Stress in Patients with Myalgic Encephalomyelitis/Chronic Fatigue Syndrome: A Pilot Project. Int. J. Mol. Sci..

[19] Raijmakers R.P.H., Jansen A.F.M., Keijmel S.P., Ter Horst R., Roerink M.E., Novakovic B., Joosten L.A.B., Van Der Meer J.W.M., Netea M.G., Bleeker-Rovers C.P. (2019). A possible role for mitochondrial-derived peptides humanin and MOTS-c in patients with Q fever fatigue syndrome and chronic fatigue syndrome. J. Transl. Med..

[20] Germain A., Levine S.M., Hanson M.R. (2021). In-Depth Analysis of the Plasma Proteome in ME/CFS Exposes Disrupted Ephrin-Eph and Immune System Signaling. Proteomes.

[21] Giloteaux L., Glass K.A., Germain A., Franconi C.J., Zhang S., Hanson M.R. (2024). Dysregulation of extracellular vesicle protein cargo in female myalgic encephalomyelitis/chronic fatigue syndrome cases and sedentary controls in response to maximal exercise. J. Extracell. Vesicles.

[22] Missailidis D., Annesley S.J., Allan C.Y., Sanislav O., Lidbury B.A., Lewis D.P., Fisher P.R. (2020). An Isolated Complex V Inefficiency and Dysregulated Mitochondrial Function in Immortalized Lymphocytes from ME/CFS Patients. Int. J. Mol. Sci..

[23] Nunes J.M., Kruger A., Proal A., Kell D.B., Pretorius E. (2022). The Occurrence of Hyperactivated Platelets and Fibrinaloid Microclots in Myalgic Encephalomyelitis/Chronic Fatigue Syndrome (ME/CFS). Pharmaceuticals.

[24] GTEx Consortium (2013). The Genotype-Tissue Expression (GTEx) project. Nat. Genet..

[25] de Leeuw C.A., Mooij J.M., Heskes T., Posthuma D. (2015). MAGMA: Generalized gene-set analysis of GWAS data. PLoS Comput. Biol..

[26] Stratford J.K., Carnes M.U., Willis C., Minto M.S., Elnimeiry L., Mathur R., Schu M., Quach B.C., Carter J., Nolen T. (2025). Identifying compounds to treat opiate use disorder by leveraging multi-omic data integration and multiple drug repurposing databases. Transl. Psychiatry.

[27] Morris G., Maes M. (2014). Mitochondrial dysfunctions in Myalgic Encephalomyelitis / chronic fatigue syndrome explained by activated immuno-inflammatory, oxidative and nitrosative stress pathways. Metab. Brain Dis..

[28] Syed A.M., Karius A.K., Ma J., Wang P., Hwang P.M. (2025). Mitochondrial Dysfunction in Myalgic Encephalomyelitis/Chronic Fatigue Syndrome. Physiology.

[29] Wood E., Hall K.H., Tate W. (2021). Role of mitochondria, oxidative stress and the response to antioxidants in myalgic encephalomyelitis/chronic fatigue syndrome: A possible approach to SARS-CoV-2 ‘long-haulers’?. Chronic Dis. Transl. Med..

[30] Hoel F., Hoel A., Pettersen I.K.N., Rekeland I.G., Risa K., Alme K., Sørland K., Fosså A., Lien K., Herder I. (2021). A map of metabolic phenotypes in patients with myalgic encephalomyelitis/chronic fatigue syndrome. JCI Insight.

[31] Davis L., Higgs M., Snaith A., Lodge T.A., Strong J., Espejo-Oltra J.A., Kujawski S., Zalewski P., Pretorius E., Hoerger M. (2025). Dysregulation of lipid metabolism, energy production, and oxidative stress in myalgic encephalomyelitis/chronic fatigue syndrome, Gulf War Syndrome and fibromyalgia. Front. Neurosci..

[32] Fletcher M.A., Zeng X.R., Maher K., Levis S., Hurwitz B., Antoni M., Broderick G., Klimas N.G. (2010). Biomarkers in Chronic Fatigue Syndrome: Evaluation of Natural Killer Cell Function and Dipeptidyl Peptidase IV/CD26. PLoS ONE.

[33] Marshall-Gradisnik S., Huth T., Chacko A., Johnston S., Smith P., Staines D. (2016). Natural killer cells and single nucleotide polymorphisms of specific ion channels and receptor genes in myalgic encephalomyelitis/chronic fatigue syndrome. Appl. Clin. Genet..

[34] Lorusso L., Mikhaylova S.V., Capelli E., Ferrari D., Ngonga G.K., Ricevuti G. (2009). Immunological aspects of chronic fatigue syndrome. Autoimmun. Rev..

[35] Wirth K.J., Scheibenbogen C., Paul F. (2021). An attempt to explain the neurological symptoms of Myalgic Encephalomyelitis/Chronic Fatigue Syndrome. J. Transl. Med..

[36] Murga Gandasegui I., Aranburu Laka L., Gargiulo P.-Á., Gómez-Esteban J.-C., Lafuente Sánchez J.-V. (2021). Myalgic Encephalomyelitis/Chronic Fatigue Syndrome: A Neurological Entity?. Medicina.

[37] Das S., Taylor K., Kozubek J., Sardell J., Gardner S. (2022). Genetic risk factors for ME/CFS identified using combinatorial analysis. J. Transl. Med..

[38] Dibble J.J., McGrath S.J., Ponting C.P. (2020). Genetic risk factors of ME/CFS: A critical review. Human Mol. Genet..

[39] Hajdarevic R., Lande A., Mehlsen J., Rydland A., Sosa D.D., Strand E.B., Mella O., Pociot F., Fluge Ø., Lie B.A. (2022). Genetic association study in myalgic encephalomyelitis/chronic fatigue syndrome (ME/CFS) identifies several potential risk loci. Brain Behav. Immun..

[40] Schlauch K.A., Khaiboullina S.F., De Meirleir K.L., Rawat S., Petereit J., Rizvanov A.A., Blatt N., Mijatovic T., Kulick D., Palotás A. (2016). Genome-wide association analysis identifies genetic variations in subjects with myalgic encephalomyelitis/chronic fatigue syndrome. Transl. Psychiatry.

[41] Smith A.K., Fang H., Whistler T., Unger E.R., Rajeevan M.S. (2011). Convergent Genomic Studies Identify Association of GRIK2 and NPAS2 with Chronic Fatigue Syndrome. Neuropsychobiology.

[42] Ueland M., Hajdarevic R., Mella O., Strand E.B., Sosa D.D., Saugstad O.D., Fluge Ø., Lie B.A., Viken M.K. (2022). No replication of previously reported association with genetic variants in the T cell receptor alpha (TRA) locus for myalgic encephalomyelitis/chronic fatigue syndrome (ME/CFS). Transl. Psychiatry.

[43] Sudlow C., Gallacher J., Allen N., Beral V., Burton P., Danesh J., Downey P., Elliott P., Green J., Landray M. (2015). UK biobank: An open access resource for identifying the causes of a wide range of complex diseases of middle and old age. PLoS Med..

[44] Denny J.C., Rutter J.L., Goldstein D.B., Philippakis A., Smoller J.W., Jenkins G., Dishman E., All of Us Research Program Investigators, All of Us Research Program Investigators (2019). The “All of Us” Research Program. N. Engl. J. Med..

[45] Beentjes S.V., Miralles Méharon A., Kaczmarczyk J., Cassar A., Samms G.L., Hejazi N.S., Khamseh A., Ponting C.P. (2025). Replicated blood-based biomarkers for myalgic encephalomyelitis not explicable by inactivity. EMBO Mol. Med..

[46] Zheng Y., Wei Z., Wang T. (2023). MOTS-c: A promising mitochondrial-derived peptide for therapeutic exploitation. Front. Endocrinol..

[47] von Walden F., Fernandez-Gonzalo R., Norrbom J., Emanuelsson E.B., Figueiredo V.C., Gidlund E.-K., Norrbrand L., Liu C., Sandström P., Hansson B. (2021). Acute endurance exercise stimulates circulating levels of mitochondrial-derived peptides in humans. J. Appl. Physiol..

[48] Reynolds J.C., Lai R.W., Woodhead J.S.T., Joly J.H., Mitchell C.J., Cameron-Smith D., Lu R., Cohen P., Graham N.A., Benayoun B.A. (2021). MOTS-c is an exercise-induced mitochondrial-encoded regulator of age-dependent physical decline and muscle homeostasis. Nat. Commun..

[49] Thevis M., Schänzer W. (2016). Emerging drugs affecting skeletal muscle function and mitochondrial biogenesis-Potential implications for sports drug testing programs. Rapid Commun. Mass. Spectrom..

[50] FDA (2024). Certain Bulk Drug Substances for Use in Compounding that May Present Significant Safety Risks. https://www.fda.gov/drugs/human-drug-compounding/certain-bulk-drug-substances-use-compounding-may-present-significant-safety-risks.

[51] Perishable (2024). What is the MOTS-c peptide?|USADA. https://www.usada.org/spirit-of-sport/what-is-mots-c-peptide.

[52] Zheng Z., Zong Y., Ma Y., Tian Y., Pang Y., Zhang C., Gao J. (2024). Glucagon-like peptide-1 receptor: Mechanisms and advances in therapy. Signal Transduct. Target. Ther..

[53] Biffl C., Ashby E., Liao J., Snair M. (2024). Forum on Microbial Threats; Forum on Neuroscience and Nervous System Disorders; Board on Global Health; Board on Health Sciences Policy; Health and Medicine Division; National Academies of Sciences, Engineering, and Medicine. Toward a Common Research Agenda in Infection-Associated Chronic Illnesses: Proceedings of a Workshop.

[54] Molnar T., Lehoczki A., Fekete M., Varnai R., Zavori L., Erdo-Bonyar S., Simon D., Berki T., Csecsei P., Ezer E. (2024). Mitochondrial dysfunction in long COVID: Mechanisms, consequences, and potential therapeutic approaches. Geroscience.

[55] Grossini E., Concina D., Rinaldi C., Russotto S., Garhwal D., Zeppegno P., Gramaglia C., Kul S., Panella M. (2021). Association Between Plasma Redox State/Mitochondria Function and a Flu-Like Syndrome/COVID-19 in the Elderly Admitted to a Long-Term Care Unit. Front. Physiol..

[56] Noonong K., Chatatikun M., Surinkaew S., Kotepui M., Hossain R., Bunluepuech K., Noothong C., Tedasen A., Klangbud W.K., Imai M. (2023). Mitochondrial oxidative stress, mitochondrial ROS storms in long COVID pathogenesis. Front. Immunol..

[57] Peacock B.N., Gherezghiher T.B., Hilario J.D., Kellermann G.H. (2015). New insights into Lyme disease. Redox Biol..

[58] Whirl-Carrillo M., McDonagh E.M., Hebert J.M., Gong L., Sangkuhl K., Thorn C.F., Altman R.B., Klein T.E. (2012). Pharmacogenomics Knowledge for Personalized Medicine. Clin. Pharmacol. Ther..

[59] Polegato B.F., Pereira A.G., Azevedo P.S., Costa N.A., Zornoff L.A.M., Paiva S.A.R., Minicucci M.F. (2019). Role of Thiamin in Health and Disease. Nut Clin. Prac..

[60] El-Hattab A.W., Zarante A.M., Almannai M., Scaglia F. (2017). Therapies for mitochondrial diseases and current clinical trials. Mol. Genet. Metab..

[61] Mantle D., Hargreaves I.P., Domingo J.C., Castro-Marrero J. (2024). Mitochondrial Dysfunction and Coenzyme Q10 Supplementation in Post-Viral Fatigue Syndrome: An Overview. Int. J. Mol. Sci..

[62] Maksoud R., Balinas C., Holden S., Cabanas H., Staines D., Marshall-Gradisnik S. (2021). A systematic review of nutraceutical interventions for mitochondrial dysfunctions in myalgic encephalomyelitis/chronic fatigue syndrome. J. Transl. Med..

[63] Maltsev D. (2022). A comparative study of valaciclovir, valganciclovir, and artesunate efficacy in reactivated HHV-6 and HHV-7 infections associated with chronic fatigue syndrome/myalgic encephalomyelitis. Microbiol. Immunol..

[64] Yang S., Tian M., Dai Y., Wang R., Yamada S., Feng S., Wang Y., Chhangani D., Ou T., Li W. (2024). Infection and chronic disease activate a systemic brain-muscle signaling axis. Sci. Immunol..

[65] Barrett T., Wilhite S.E., Ledoux P., Evangelista C., Kim I.F., Tomashevsky M., Marshall K.A., Phillippy K.H., Sherman P.M., Holko M. (2013). NCBI GEO: Archive for functional genomics data sets--update. Nucleic Acids Res..

[66] Bouquet J., Li T., Gardy J.L., Kang X., Stevens S., Stevens J., VanNess M., Snell C., Potts J., Miller R.R. (2019). Whole blood human transcriptome and virome analysis of ME/CFS patients experiencing post-exertional malaise following cardiopulmonary exercise testing. PLOS ONE.

[67] Presson A.P., Sobel E.M., Papp J.C., Suarez C.J., Whistler T., Rajeevan M.S., Vernon S.D., Horvath S. (2008). Integrated Weighted Gene Co-expression Network Analysis with an Application to Chronic Fatigue Syndrome. BMC Syst Biol..

[68] Fang H., Xie Q., Boneva R., Fostel J., Perkins R., Tong W. (2006). Gene expression profile exploration of a large dataset on chronic fatigue syndrome. Pharmacogenomics.

[69] Ciregia F., Kollipara L., Giusti L., Zahedi R.P., Giacomelli C., Mazzoni M.R., Giannaccini P., Scarpellini P., Urbani A., Sickmann A. (2016). Bottom-up proteomics suggests an association between differential expression of mitochondrial proteins and chronic fatigue syndrome. Transl Psychiatry.

[70] Fernandez-Guerra P., Gonzalez-Ebsen A.C., Boonen S.E., Courraud J., Gregersen N., Mehlsen J., Palmfeldt J., Olsen R.K.J., Brinth L.S. (2021). Bioenergetic and Proteomic Profiling of Immune Cells in Myalgic Encephalomyelitis/Chronic Fatigue Syndrome Patients: An Exploratory Study. Biomolecules.

[71] Sweetman E., Kleffmann T., Edgar C., De Lange M., Vallings R., Tate W. (2020). A SWATH-MS analysis of Myalgic Encephalomyelitis/Chronic Fatigue Syndrome peripheral blood mononuclear cell proteomes reveals mitochondrial dysfunction. J Transl Med..

[72] Love M.I., Huber W., Anders S. (2014). Moderated estimation of fold change and dispersion for RNA-seq data with DESeq2. Genome Biol..

[73] Gold L., Ayers D., Bertino J., Bock C., Bock A., Brody E.N., Carter J., Dalby A.B., Eaton B.E., Fitzwater T. (2010). Aptamer-based multiplexed proteomic technology for biomarker discovery. PLoS ONE.

[74] Thompson A., Schäfer J., Kuhn K., Kienle S., Schwarz J., Schmidt G., Neumann T., Johnstone R., Mohammed A.K.A., Hamon C. (2003). Tandem mass tags: A novel quantification strategy for comparative analysis of complex protein mixtures by MS/MS. Anal. Chem..

[75] Satija R., Farrell J.A., Gennert D., Schier A.F., Regev A. (2015). Spatial reconstruction of single-cell gene expression data. Nat. Biotechnol..

[76] Watanabe K., Taskesen E., van Bochoven A., Posthuma D. (2017). Functional mapping and annotation of genetic associations with FUMA. Nat. Commun..

[77] Kelleher K.J., Sheils T.K., Mathias S.L., Yang J.J., Metzger V.T., Siramshetty V.B., Nguyen D.-T., Jensen L.J., Vidović D., Schürer S.C. (2023). Pharos 2023: An integrated resource for the understudied human proteome. Nucleic Acids Res..

[78] Buniello A., Suveges D., Cruz-Castillo C., Llinares M.B., Cornu H., Lopez I., Tsukanov K., Roldán-Romero J.M., Mehta C., Fumis L. (2025). Open Targets Platform: Facilitating therapeutic hypotheses building in drug discovery. Nucleic Acids Res..

[79] Wang Y., Zhang S., Li F., Zhou Y., Zhang Y., Wang Z., Zhang R., Zhu J., Ren Y., Tan Y. (2020). Therapeutic target database 2020: Enriched resource for facilitating research and early development of targeted therapeutics. Nucleic Acids Res..

[80] Knox C., Wilson M., Klinger C.M., Franklin M., Oler E., Wilson A., Pon A., Cox J., Chin N.E.L., Strawbridge S.A. (2024). DrugBank 6.0: The DrugBank Knowledgebase for 2024. Nucleic Acids Res..

[81] Giloteaux L., Li J., Hornig M., Lipkin W.I., Ruppert D., Hanson M.R. (2023). Proteomics and cytokine analyses distinguish myalgic en-cephalomyelitis/chronic fatigue syndrome cases from controls. J. Transl. Med..

