# Supplementary material for: Systematic Examination of Gene Expression and Proteomic Evidence Across Tissues Supports the Role of Mitochondrial Dysregulation in ME/CFS
Source: Int J Mol Sci. 2026 Feb 19;27(4):1997. doi: 10.3390/ijms27041997 (PMC12940889; doi:10.3390/ijms27041997)
Supplement: Supplementary file 1 [file ijms-27-01997-s001.zip › mapMECFS_DRUG_supplementary_materials-revised.pdf]

Supplementary Materials

Systematic examination of gene expression and proteomic evidence across tissues supports the role of mitochondrial dysregulation in ME/CFS

Gregory R. Keele[1*], Mike Enger[1*], Quinn Barnette[1], Roman Ruiz-Esparza[1], Manuel Alvarado[1], Ravi Mathur [1], Jeran K Stratford[1], Stephanie N. Giamberardino[1], Linda Morris Brown[1], Bradley T. Webb[2,3,4], Megan Ulmer Carnes[1]

[1]RTI International, Research Triangle Park, NC, United States

[2] Department of Population Health Sciences, Geisinger, Danville, PA, United States

[3] Center for Substance Use Research and Education, Geisinger, Danville, PA, United States

[4] Department of Psychiatry, Virginia Institute for Psychiatric and Behavioral Genetics, Virginia Commonwealth University, Richmond, VA, United States

[*]Contributed equally and are designated co-first authors

SUPPLEMENTAL FIGURES

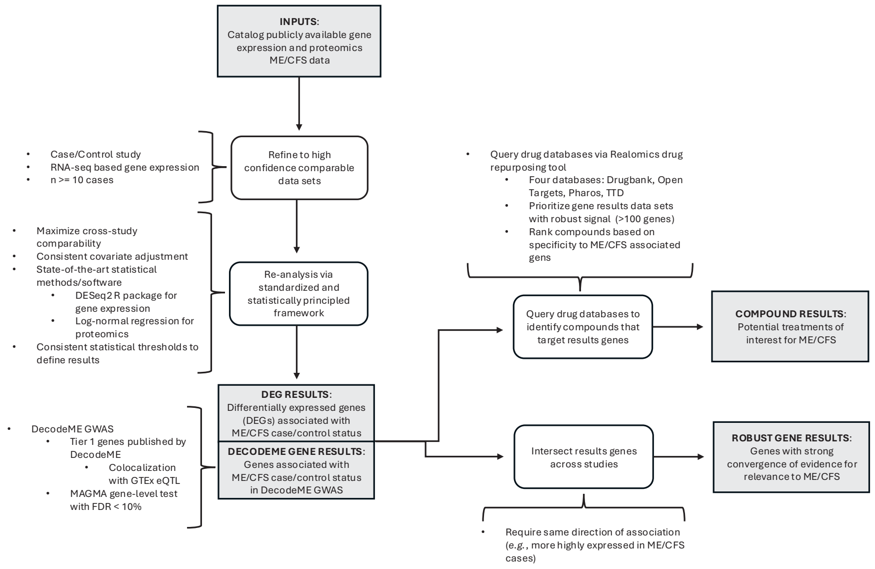

Supplementary Figure S1. Diagram of analytic framework used in curating gene expression and proteomics studies, analysis within study, and integration across studies.

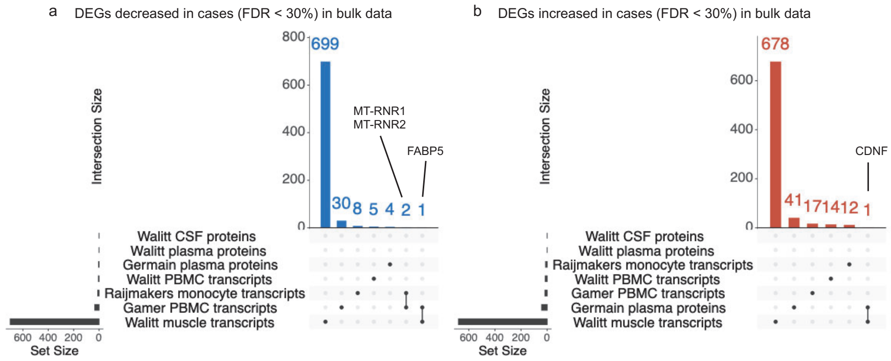

Supplementary Figure S2. Comparison of lenient differential expression results (FDR < 30%) across eight datasets from bulk tissue samples. Counts of genes with (a) decreased and (b) increased expression in ME/CFS cases (FDR < 30%) across the eight datasets. Horizontal bars represent the number of genes in each dataset. Vertical bars represent the number of differentially expressed genes (DEGs) observed across datasets. Dots and line segments indicate datasets for each DEG set. Genes detected across multiple datasets are highlighted.

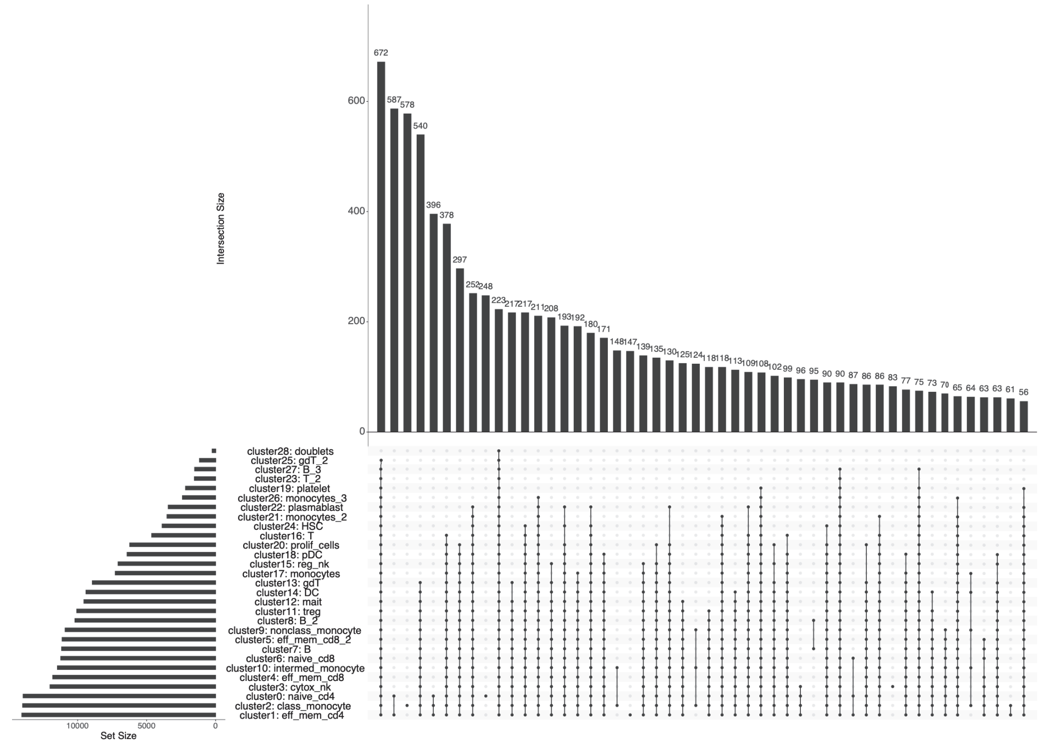

Supplementary Figure S3. Overlap of genes across 29 clusters defined from PBMC scRNA-seq in ME/CFS cases and controls. Genes were filtered based on 70% of samples have >2 pseudobulk counts. Horizontal bars represent the number of genes in each cluster. Vertical bars represent the number of genes observed across clusters. Dots and line segments indicate clusters for each intersection set.

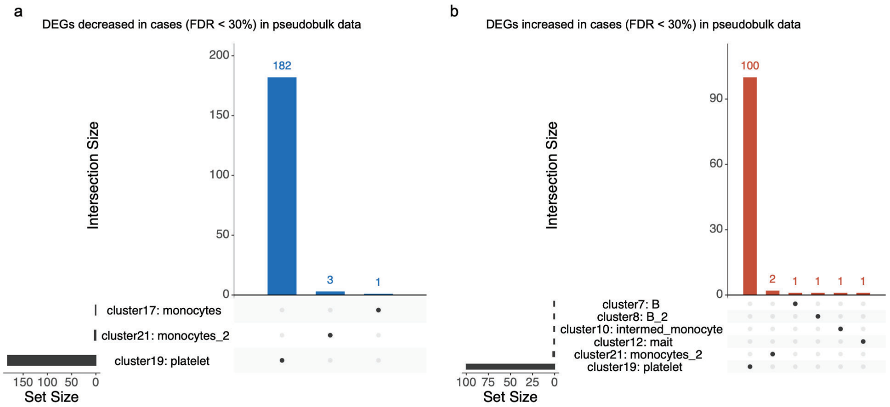

Supplementary Figure S4. Comparison of lenient differential expression results (FDR < 30%) across 28 pseudobulk cluster datasets derived from PBMC samples. Counts of genes with (a) decreased and (b) increased expression in ME/CFS cases (FDR < 30%) across the eight datasets. Horizontal bars represent the number of genes in each dataset. Vertical bars represent the number of differentially expressed genes (DEGs) observed across datasets. Dots indicate clusters for each DEG set.

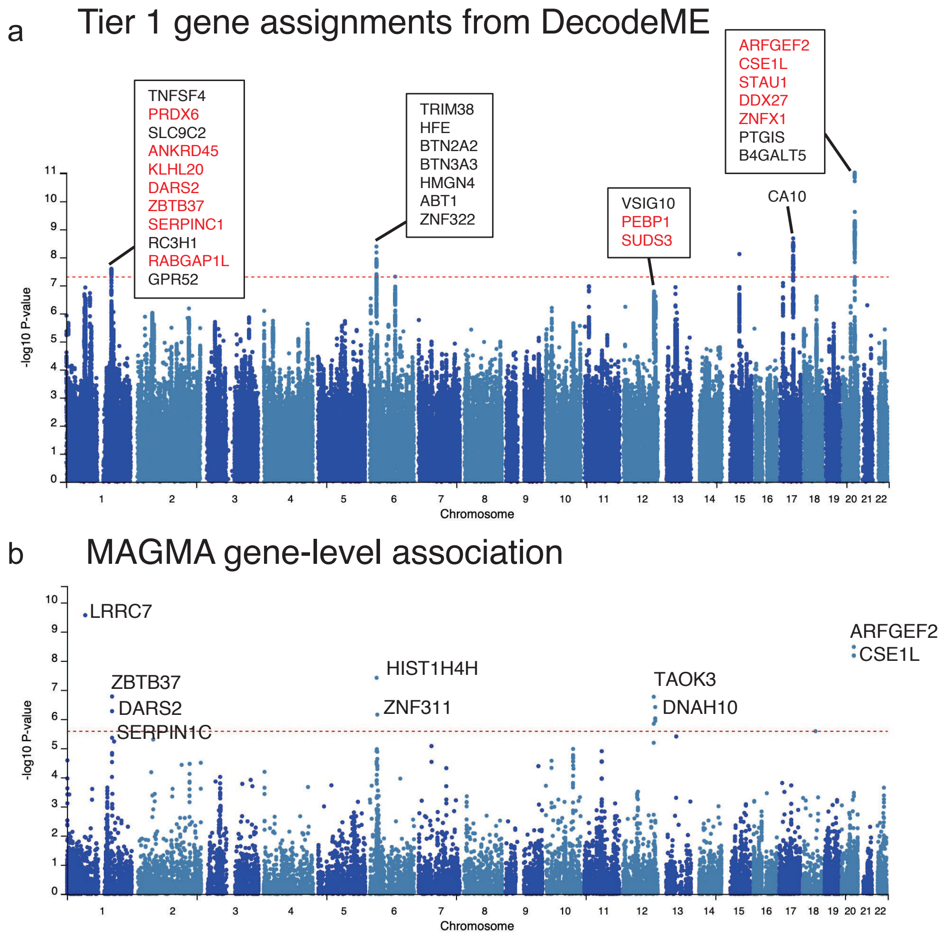

Supplementary Figure S5. ME/CFS-associated genes from DecodeME GWAS. (a) Manhattan plot of SNP-level associations with all 29 tier 1 genes highlighted. Genes with red labels were also surpassed the MAGMA FDR < 10% threshold. (b) Manhattan plot of gene-level associations from MAGMA with select genes highlighted. Red dashed lines represent Bonferroni-adjusted significance thresholds. Plot is also found in Figure 4d.

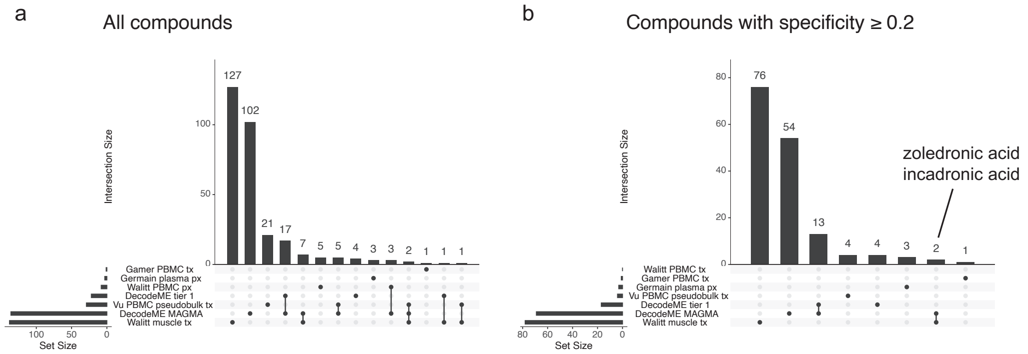

Supplementary Figure S6. Comparison of compounds targeting candidate genes across studies. Counts of compounds that target candidate genes across studies with (a) no filtering based on compound specificity and (b) filtering to ≥ 0.2 specificity to candidate genes. Zoledronic acid and incadronic acid labels added to highlight overlap across two studies. Horizontal bars represent the number of compounds in each dataset. Vertical bars represent the number of compounds observed across datasets. Dots and line segments indicate datasets for each intersection set.

SUPPLEMENTARY TABLES

Supplementary Table S1. Surveyed gene expression and proteomics datasets considered for inclusion in this study. Results and code generated from these data are available on mapMECFS at https://mapmecfs.org/dataset/keele-enger-systematic-examination-of-gene-expression-and-proteomics-code.

Data type	Assay	Publication	Tissue	Sample size[a]	Study design	Data Source	Additional Raw Data Source	Inclusion status
**Gene Expression**	scRNA-seq	Vu et al. 2024[1]	PBMC	30/28	Two samples per subject: Prior to CPET and 24 hr post.	Author Request	GSE214283[b]	Include
	Bulk RNA-seq		Classical monocytes (CD14+) from PBMC	4/4	All females with only post CPET.	NA	GSE214282[b]	Exclude
			Plasma derived particles containing platelets	3/3	All females with pre and post CPET.	NA	GSE236402[b]	Exclude
		Van Booven et al. 2023[2]<br><br>Gamer et al. 2023[3]	PBMC	33/34	38 Females and 19 males. Prior to CPET, at maximal exertion, and 4 hr post.	**mapMECFS** https://mapmecfs.org/dataset/me-cfs-case-control-rna-seq-study-lubov-nathanson	GSE227375[b]	Include
		Walitt et al. 2024[4]	PBMC	15/11	Males and females. Post-infectious cases.	**mapMECFS** https://mapmecfs.org/organization/nih-intramural	GSE251872[b]	Include

			Muscle	12/13	Males and females. Post-infectious cases.	**mapMECFS** https://mapmecfs.org/organization/nih-intramural	GSE245661[b]	Include
		Raijmakers et al. 2019[5]	Monocytes from PBMC	11/10	Males and females.	**mapMECFS** https://mapmecfs.org/dataset/me-cfs-and-qfs-case-control-rna-expression-study-gse130353	GSE130353[b]	Include
		Bouquet et al. 2019[6]	Whole blood	14/11	All females. CPET on two consecutive days (days 1 and 2). Whole blood samples taken on days 1, 2, 3, and 7.	**mapMECFS** https://mapmecfs.org/dataset/me-cfs-case-control-rna-expression-study-following-exercise-gse128078	GSE128078[b]	Exclude
	Gene array	Jeffrey et al. 2019[7]	PBMC	33/21	Males and females.	NA	NA	Exclude
		Presson et al. 2008[8]	PBMC	127/0	Males and females.	NA	NA	Exclude
		Fang et al. 2006[9]	PBMC	167/0	NA	NA	NA	Exclude
**Proteomics**	SomaScan	Walitt et al. 2024[4]	Plasma	15/18	Males and females. Post-infectious cases.	**mapMECFS** https://mapmecfs.org/orga	GSE254030[b]	Include

						nization/nih-intramural		
			CSF	15/18	Males and females. Post-infectious cases.	**mapMECFS** https://mapmecfs.org/organization/nih-intramural	GSE251790[b]	Include
		Germain et al. 2021[10]	Plasma	20/20	All females.	**mapMECFS** https://mapmecfs.org/dataset/me-cfs-case-control-plasma-proteomics	NA	Include
	Mass spectrometry	Giloteaux et al. 2023[11]	Extracellular vesicles from plasma	18/17	All females. Prior to CPET and 15 min and 24 hr post. TMT mass spectrometry.	**mapMECFS** https://mapmecfs.org/dataset/dysregulation-of-ev-protein-cargo-in-me-cfs-cases-and-sedentary-ctrls-in-response-to-max-exercise	NA	Include
		Giloteaux et al. 2023[12]	Plasma	49/49	Males and females. UPLC mass spectrometry.	**mapMECFS** https://mapmecfs.org/dataset/multiomic-analyses-distinguish-me-cfs-cases-from-	ProteomeXchange PXD016622	Exclude

						controls-ev-cytokines		
		Vu et al. 2024[1]	Classical monocytes (CD14+) from PBMC	2/4	All females and post CPET.	NA	Supplemental file at journal	Excluded
		Sweetman et al. 2020[13]	PBMC	11/9	Males and females. SWATH mass spectrometry.	**mapMECFS** https://mapmecfs.org/dataset/a-swath-ms-analysis-of-mecfs-proteomes	NA	Exclude
		Milivojevic et al. 2020[14]	Plasma	39/41	Males and females. UPLC mass spectrometry.	**mapMECFS** https://mapmecfs.org/dataset/me-cfs-case-control-plasma-proteome-analysis	ProteomeXchange PXD016622	Exclude
		Fernandez-Guerra et al. 2021[15]	PBMC	6/6	All females. TMT mass spectrometry.	NA	Supplemental file at journal	Exclude
		Ciregia et al. 2016[16]	Mitochondrial proteins derived from platelets	1/1	Male monozygotic twins that were discordant for ME/CFS. Follow up in 45 cases / 45 controls with western blots for 3 proteins.	NA	NA	Exclude

[a]Case/Control; [b]Gene Expression Omnibus (GEO)

Acronyms: peripheral blood mononuclear cells (PBMC), cardiopulmonary exercise test (CPET), cerebral spinal fluid (CSF), Ultra Performance Liquid Chromatography (UPLC), Sequential Windows Acquisition of all Theoretical Fragment Ion Spectra (SWATH), Tandem Mass Tag (TMT)

SUPPLEMENTARY FILES

Supplemental File S1. Re-analysis reports for each dataset included in study. Includes R code to perform analysis and generate results figures. (HTML)

Supplemental File S2. Differential expression results for each dataset included in study. Each sheet includes results for a different dataset. All genes are included. (XLSX)

Supplemental File S3. Realomics results from differentially expressed genes (FDR < 10%) and DecodeME GWAS genes. Each sheet includes results for a different dataset. (XLSX)

REFERENCES

1. Vu LT, Ahmed F, Zhu H, Iu DSH, Fogarty EA, Kwak Y, et al. Single-cell transcriptomics of the immune system in ME/CFS at baseline and following symptom provocation. Cell Rep Med. 2024;5:101373.

2. Van Booven DJ, Gamer J, Joseph A, Perez M, Zarnowski O, Pandya M, et al. Stress-Induced Transcriptomic Changes in Females with Myalgic Encephalomyelitis/Chronic Fatigue Syndrome Reveal Disrupted Immune Signatures. Int J Mol Sci. 2023;24:2698.

3. Gamer J, Van Booven DJ, Zarnowski O, Arango S, Elias M, Kurian A, et al. Sex-Dependent Transcriptional Changes in Response to Stress in Patients with Myalgic Encephalomyelitis/Chronic Fatigue Syndrome: A Pilot Project. Int J Mol Sci. 2023;24:10255.

4. Walitt B, Singh K, LaMunion SR, Hallett M, Jacobson S, Chen K, et al. Deep phenotyping of post-infectious myalgic encephalomyelitis/chronic fatigue syndrome. Nat Commun. 2024;15:907.

5. Raijmakers RPH, Jansen AFM, Keijmel SP, Ter Horst R, Roerink ME, Novakovic B, et al. A possible role for mitochondrial-derived peptides humanin and MOTS-c in patients with Q fever fatigue syndrome and chronic fatigue syndrome. J Transl Med. 2019;17:157.

6. Bouquet J, Li T, Gardy JL, Kang X, Stevens S, Stevens J, et al. Whole blood human transcriptome and virome analysis of ME/CFS patients experiencing post-exertional malaise following cardiopulmonary exercise testing. Switzer WM, editor. PLOS ONE. 2019;14:e0212193.

7. Jeffrey MG, Nathanson L, Aenlle K, Barnes ZM, Baig M, Broderick G, et al. Treatment Avenues in Myalgic Encephalomyelitis/Chronic Fatigue Syndrome: A Split-gender Pharmacogenomic Study of Gene-expression Modules. Clin Ther. 2019;41:815-835.e6.

8. Presson AP, Sobel EM, Papp JC, Suarez CJ, Whistler T, Rajeevan MS, et al. Integrated Weighted Gene Co-expression Network Analysis with an Application to Chronic Fatigue Syndrome. BMC Syst Biol. 2008;2:95.

9. Fang H, Xie Q, Boneva R, Fostel J, Perkins R, Tong W. Gene expression profile exploration of a large dataset on chronic fatigue syndrome. Pharmacogenomics. 2006;7:429–40.

10. Germain A, Levine SM, Hanson MR. In-Depth Analysis of the Plasma Proteome in ME/CFS Exposes Disrupted Ephrin-Eph and Immune System Signaling. Proteomes. 2021;9:6.

11. Giloteaux L, Glass KA, Germain A, Franconi CJ, Zhang S, Hanson MR. Dysregulation of extracellular vesicle protein cargo in female myalgic encephalomyelitis/chronic fatigue syndrome cases and sedentary controls in response to maximal exercise. J Extracell Vesicles. 2024;13:e12403.

12. Giloteaux L, Li J, Hornig M, Lipkin WI, Ruppert D, Hanson MR. Proteomics and cytokine analyses distinguish myalgic encephalomyelitis/chronic fatigue syndrome cases from controls. J Transl Med. 2023;21:322.

13. Sweetman E, Kleffmann T, Edgar C, De Lange M, Vallings R, Tate W. A SWATH-MS analysis of Myalgic Encephalomyelitis/Chronic Fatigue Syndrome peripheral blood mononuclear cell proteomes reveals mitochondrial dysfunction. J Transl Med. 2020;18:365.

14. Milivojevic M, Che X, Bateman L, Cheng A, Garcia BA, Hornig M, et al. Plasma proteomic profiling suggests an association between antigen driven clonal B cell expansion and ME/CFS. PloS One. 2020;15:e0236148.

15. Fernandez-Guerra P, Gonzalez-Ebsen AC, Boonen SE, Courraud J, Gregersen N, Mehlsen J, et al. Bioenergetic and Proteomic Profiling of Immune Cells in Myalgic Encephalomyelitis/Chronic Fatigue Syndrome Patients: An Exploratory Study. Biomolecules. 2021;11:961.

16. Ciregia F, Kollipara L, Giusti L, Zahedi RP, Giacomelli C, Mazzoni MR, et al. Bottom-up proteomics suggests an association between differential expression of mitochondrial proteins and chronic fatigue syndrome. Transl Psychiatry. 2016;6:e904–e904.
