# Supplementary material for: Systematic Examination of Gene Expression and Proteomic Evidence Across Tissues Supports the Role of Mitochondrial Dysregulation in ME/CFS
Source: Int J Mol Sci. 2026 Feb 19;27(4):1997. doi: 10.3390/ijms27041997 (PMC12940889; doi:10.3390/ijms27041997)
Supplement: Supplementary file 1 [file ijms-27-01997-s001.zip › supplemental_file1_report.html]

Report of re-analyses performed in Keele & Enger et al. manuscript for gene transcript/protein expression studies in ME/CFS


# Report of re-analyses performed in Keele & Enger *et al.* manuscript for gene transcript/protein expression studies in ME/CFS

#### Greg Keele (`gkeele@rti.org`)

#### June 27, 2025

##### **Note: To view this report optimally, please see the output HTML file and open it in a web browser. Some features in the report may not otherwise display correctly.**

```
## Load required R packages
library("readxl")
library("tidyverse")
library("ggrepel")
library("ggbeeswarm")
library("DT")
library("DESeq2")
library("FDRestimation")
library("pcaMethods")
library("UpSetR")
library("data.table")
```

This report includes re-analyses for nine data sets, representing six
publications. Overall, the data consist of bulk RNA-seq data sets from
peripheral blood mononuclear cells (PBMCs), muscle tissue, and monocytes
(derived from PBMCs); a scRNA-seq data set from PBMCs (summarized into
29 cell-type annotated pseudobulk clusters); SomaScan data sets from
plasma and cerebral spinal fluid (CSF); and a tandem mass tag (TMT) mass
spectrometry data set from extracellular vesicles (EVs) (derived from
plasma). See specific analysis for more details.

# Data set reports

## Vu *et al.* 2024 scRNA-seq in PBMCs

### Introduction

These data were published as part of Vu *et al.*
2024 in *Cell Reports Medicine*. This study included
single-cell RNA sequencing (scRNA-seq) of PBMC samples from ME/CFS cases
and controls to investigate postexertional malaise (PEM) via
cardiopulmonary exercise testing (CPET) challenge. The scRNA-seq data
were used to infer cell-type clusters, which were then used to estimate
pseudobulk gene expression profiles for cell-types.

| Study | Data Type | Tissue | Case/Control | Notes |
| --- | --- | --- | --- | --- |
| Vu *et al.* 2024 | Gene expression (Pseudobulk derived from single cell RNA-seq) | PBMC | 30/28 | Females and males |

---

### Analysis

#### Overview

Vu *et
al.* 2024 performed the differential expression analysis of
pseudobulk data using `DESeq2`. They do not describe
covariates they included in the model. Here we also use
`DESeq2` and including sex as a covariate, as well as BMI,
following Walitt *et al.* 2024.

#### Load data

```
## Set to the appropriate directory in your environment
setwd("<Your Directory>")
```

```
## Cell-type cluster annotations
cluster_annot <- read.table("data/pseudobulk/vu_cluster_annot.tsv", header = TRUE, sep = "\t")
cluster_names <- paste0("cluster", cluster_annot$cluster, ": ", cluster_annot$celltype)

## Case/control and covariates (e.g., sex)
sample_annot <- read.table("data/pseudobulk/vu_sample_annot.tsv", header = TRUE, sep = "\t")

## Create a list of data.frames, each element corresponding to a cluster
raw_list <- norm_list <- sample_annot_list <- list()
for (i in 1:nrow(cluster_annot)) {
  ## Raw gene count
  raw_list[[i]] <- read.table(paste0("data/pseudobulk/clusters/", 
                                     paste("cluster", cluster_annot$cluster[i], "scrnaseq_pseudobulk_assay_data_export_for_rti.tsv", sep = "_")),
                              header = TRUE, sep = "\t") %>%
    column_to_rownames("Molecule")
  
  ## Normalized gene count for plotting
  norm_list[[i]] <- varianceStabilizingTransformation(object = as.matrix(raw_list[[i]])) %>%
    as.data.frame
  
  sample_annot_list[[i]] <- data.frame(sample_id = colnames(raw_list[[i]])) %>%
    mutate(ParticipantID = gsub(x = sample_id, pattern = "\\.D.+$", replacement = "", perl = TRUE)) %>%
    left_join(sample_annot %>%
                mutate(ParticipantID = gsub(x = ParticipantID, pattern = "-", replacement = "."))) %>%
    mutate(timepoint = ifelse(grepl(x = sample_id, pattern = "D1"), "D1", "D2"))
}
names(raw_list) <- names(norm_list) <- names(sample_annot_list) <- cluster_names

sample_annot_dat <- list_rbind(sample_annot_list, names_to = "cluster") %>%
  mutate(cluster = factor(cluster, levels = cluster_names))
```

---

#### Distribution of samples across clusters

```
## Sample breakdown across clusters
ggplot(data = sample_annot_dat %>%
         mutate(type = paste(Phenotype, timepoint)),
       aes(x = cluster, fill = type)) +
  geom_bar(stat = "count") +
  scale_fill_manual(values = c("tomato4", "tomato1", "dodgerblue4", "dodgerblue1")) +
  theme_bw() +
  theme(axis.text.x = element_text(angle = 60, hjust = 1))
```

```
## Distribution of mean gene expression (across genes), stratified by clusters
mean_dat <- list_rbind(lapply(raw_list, function(x) data.frame(mean = rowMeans(x))), names_to = "cluster") %>%
  mutate(cluster = factor(cluster, levels = cluster_names)) %>%
  filter(cluster != "cluster28: doublets")

low_count_dat <- lapply(raw_list, function(x) apply(x, 1, function(y) mean(y > 2) >= 0.3)) %>%
  lapply(., function(x) sum(x))
low_count_dat <- data.frame(count = unlist(low_count_dat),
                            cluster = names(low_count_dat)) %>%
  mutate(cluster = factor(cluster, levels = cluster_names)) %>%
  mutate(cluster = factor(cluster, levels = cluster_names)) %>%
  filter(cluster != "cluster28: doublets")

## Looking at mean gene expression levels across clusters
ggplot(data = mean_dat,
       aes(x = log10(mean + 1))) +
  geom_histogram() +
  geom_vline(xintercept = log10(2 + 1), col = "red", linetype = "dashed") +
  geom_text(data = low_count_dat,
            aes(label = count), x = 4, y = 15000) +
  theme_bw() +
  facet_wrap(~cluster)
```

There is good representation of all samples across the clusters
except for cluster28, which will be excluded from further analysis.

In all clusters, the majority of genes are not expressed or have very
low expression. Counts of genes where 30% or more of sample have > 2
counts for each cluster. Red dashed line indicates 2 counts (on the
log10(x + 1) scale.

Here we visualize how genes are distributed across clusters using an
Upset plot.

```
## Looking at the distribution of genes to clusters
all_genes <- rownames(raw_list[[1]])

## Remove cluster28
analyze_cluster_names <- cluster_names[-length(cluster_names)]

# Filter out lowly expressed genes (across all samples) for a given cluster
cluster_gene_sets <- lapply(raw_list, function(x) rownames(raw_list[[1]])[unlist(apply(x, 1, function(y) mean(y > 2) >= 0.3))])

# Making data for Upset plot
all_genes_upset_dat <- list_cbind(lapply(cluster_gene_sets, function(x) as.data.frame(as.numeric(all_genes %in% x)))) %>%
  as.matrix %>%
  as.data.frame

names(all_genes_upset_dat) <- names(cluster_gene_sets)
rownames(all_genes_upset_dat) <- all_genes

upset(data = all_genes_upset_dat,
      order.by = "freq",
      nsets = 29)
```

Notably, the largest set is genes observed in all clusters but the
29th, which is filtered out from further analysis (672). In total, 895
genes are observed in all 28 clusters that are retained for further
analysis.

---

#### Principal component analysis (PCA)

```
pca_list <- pca_var_dat_list <- pca_dat_list <- list()
## Cluster28 only has two samples, will exclude
for (i in 1:(nrow(cluster_annot) - 1)) {
  cluster_pca <- pca(object = norm_list[[i]][cluster_gene_sets[[i]],] %>%
                       t,
                     nPcs = 10)
  
  pca_list[[i]] <- cluster_pca
  
  pca_var_dat_list[[i]] <- data.frame(r2 = cluster_pca@R2, 
                                      pc = names(cluster_pca@R2)) %>%
    mutate(pc = factor(pc, levels = names(cluster_pca@R2)))
  
  pca_dat_list[[i]] <- scores(cluster_pca) %>%
    as.data.frame %>%
    rownames_to_column("sample_id") %>%
    mutate(ParticipantID = gsub(x = sample_id, pattern = "\\.D.+$", replacement = "", perl = TRUE)) %>%
    left_join(sample_annot_list[[i]])
}
names(pca_list) <- names(pca_var_dat_list) <- names(pca_dat_list) <- analyze_cluster_names

pca_var_dat <- list_rbind(pca_var_dat_list, names_to = "cluster") %>%
  mutate(cluster = factor(cluster, levels = analyze_cluster_names))
pca_dat <- list_rbind(pca_dat_list, names_to = "cluster") %>%
  mutate(cluster = factor(cluster, levels = analyze_cluster_names))
```

```
## Scree plot
ggplot(data = pca_var_dat,
       aes(x = pc, y = r2)) +
  geom_point() +
  xlab("") +
  ggtitle("Scree plots by cluster") +
  theme_bw() +
  theme(axis.text.x = element_text(angle = 45, hjust = 1)) +
  facet_wrap(~cluster)
```

##### PCA Bi-plots

###### Sex

PC2 vs PC1

```
ggplot(data = pca_dat,
       aes(x = PC1, y = PC2, col = sex)) +
  geom_point() +
  ggtitle("Bi-plots for PC2 vs PC1 colored by sex") +
  theme_bw() +
  theme(legend.position = "bottom") +
  facet_wrap(~cluster, scale = "free")
```

PC4 vs PC3

```
ggplot(data = pca_dat,
       aes(x = PC3, y = PC4, col = sex)) +
  geom_point() +
  ggtitle("Bi-plots for PC2 vs PC1 colored by sex") +
  theme_bw() +
  theme(legend.position = "bottom") +
  facet_wrap(~cluster, scale = "free")
```

PC6 vs PC5

```
ggplot(data = pca_dat,
       aes(x = PC5, y = PC6, col = sex)) +
  geom_point() +
  ggtitle("Bi-plots for PC6 vs PC5 colored by sex") +
  theme_bw() +
  theme(legend.position = "bottom") +
  facet_wrap(~cluster, scale = "free")
```

PC8 vs PC7

```
ggplot(data = pca_dat,
       aes(x = PC7, y = PC8, col = sex)) +
  geom_point() +
  ggtitle("Bi-plots for PC8 vs PC7 colored by sex") +
  theme_bw() +
  theme(legend.position = "bottom") +
  facet_wrap(~cluster, scale = "free")
```

######

###### Case/Control

PC2 vs PC1

```
ggplot(data = pca_dat,
       aes(x = PC1, y = PC2, col = Phenotype)) +
  geom_point() +
  ggtitle("Bi-plots for PC2 vs PC1 colored by case/control status") +
  scale_color_manual("Case/Control", values = c("plum", "seagreen2")) +
  theme_bw() +
  theme(legend.position = "bottom") +
  facet_wrap(~cluster, scale = "free")
```

PC4 vs PC3

```
ggplot(data = pca_dat,
       aes(x = PC3, y = PC4, col = Phenotype)) +
  geom_point() +
  ggtitle("Bi-plots for PC4 vs PC3 colored by case/control status") +
  scale_color_manual("Case/Control", values = c("plum", "seagreen2")) +
  theme_bw() +
  theme(legend.position = "bottom") +
  facet_wrap(~cluster, scale = "free")
```

PC6 vs PC5

```
ggplot(data = pca_dat,
       aes(x = PC5, y = PC6, col = Phenotype)) +
  geom_point() +
  ggtitle("Bi-plots for PC6 vs PC5 colored by case/control status") +
  scale_color_manual("Case/Control", values = c("plum", "seagreen2")) +
  theme_bw() +
  theme(legend.position = "bottom") +
  facet_wrap(~cluster, scale = "free")
```

PC8 vs PC7

```
ggplot(data = pca_dat,
       aes(x = PC7, y = PC8, col = Phenotype)) +
  geom_point() +
  ggtitle("Bi-plots for PC8 vs PC7 colored by case/control status") +
  scale_color_manual("Case/Control", values = c("plum", "seagreen2")) +
  theme_bw() +
  theme(legend.position = "bottom") +
  facet_wrap(~cluster, scale = "free")
```

###### 

---

A lot of variation in the data looks to be strongly correlated with
sex. This likely stems from X-and Y-linked genes. Notably, we do not
observe any PCs clearly correlating with case/control status. This does
not mean that no genes are differentially expressed between cases and
controls, but likely not in a large-scale sense.

#### Differential gene expression analysis

```
## Run differential gene expression analysis
pseudo_results <- list()
for (i in 1:(nrow(cluster_annot) - 1)) {
  ## D1
  covar_d1_mat <- sample_annot_list[[i]] %>%
    filter(timepoint == "D1") %>%
    mutate(sex = factor(sex),
           case = as.factor(as.numeric(Phenotype == "ME/CFS")),
           bmi_cat = as.factor(bmi_binned)) %>%
    column_to_rownames("sample_id") %>%
    dplyr::select(sex, case, bmi_cat)
  
  ## Case/control
  deseq2_d1_case_dat <- DESeqDataSetFromMatrix(countData = raw_list[[i]][cluster_gene_sets[[i]], rownames(covar_d1_mat)],
                                               colData = covar_d1_mat,
                                               design = ~ sex + bmi_cat + case)
  deseq2_d1_case_results <- DESeq(deseq2_d1_case_dat, minReplicatesForReplace = Inf)
  
  ## Sex 
  deseq2_d1_sex_dat <- DESeqDataSetFromMatrix(countData = raw_list[[i]][cluster_gene_sets[[i]], rownames(covar_d1_mat)],
                                               colData = covar_d1_mat,
                                               design = ~ case + bmi_cat + sex)
  deseq2_d1_sex_results <- DESeq(deseq2_d1_sex_dat, minReplicatesForReplace = Inf)
  
  ## Combine results
  deseq2_d1_results_dat <- results(deseq2_d1_case_results) %>%
    as.data.frame %>%
    rename_with(~ paste("case", .x, sep = "_"), .cols = -baseMean) %>%
    rownames_to_column("symbol") %>%
    left_join(results(deseq2_d1_sex_results) %>%
                as.data.frame %>%
                dplyr::select(-baseMean) %>%
                rename_with(~ paste("sex", .x, sep = "_"), .cols = everything()) %>%
                rownames_to_column("symbol")) %>%
    arrange(case_padj) %>%
    mutate(case_sig_cat = case_when(case_padj < 0.1 & case_log2FoldChange > 0 ~ "Up in cases; FDR < 0.1",
                                    case_padj < 0.3 & case_log2FoldChange > 0 ~ "Up in cases; 0.3 < FDR < 0.1",
                                    case_padj < 0.5 & case_log2FoldChange > 0 ~ "Up in cases; 0.5 < FDR < 0.3",
                                    case_padj < 0.1 & case_log2FoldChange < 0 ~ "Down in cases; FDR < 0.1",
                                    case_padj < 0.3 & case_log2FoldChange < 0 ~ "Down in cases; 0.3 < FDR < 0.1",
                                    case_padj < 0.5 & case_log2FoldChange < 0 ~ "Down in cases; 0.5 < FDR < 0.3",
                                    case_padj > 0.5 ~ "FDR > 0.5")) %>%
    mutate(case_sig_cat = factor(case_sig_cat, levels = c("Down in cases; FDR < 0.1", 
                                                          "Down in cases; 0.3 < FDR < 0.1", 
                                                          "Down in cases; 0.5 < FDR < 0.3",
                                                          "Up in cases; 0.5 < FDR < 0.3",
                                                          "Up in cases; 0.3 < FDR < 0.1",
                                                          "Up in cases; FDR < 0.1",
                                                          "FDR > 0.5"))) %>%
    mutate(sex_sig_cat = case_when(sex_padj < 0.1 & sex_log2FoldChange > 0 ~ "Up in males; FDR < 0.1",
                                   sex_padj < 0.3 & sex_log2FoldChange > 0 ~ "Up in males; 0.3 < FDR < 0.1",
                                   sex_padj < 0.5 & sex_log2FoldChange > 0 ~ "Up in males; 0.5 < FDR < 0.3",
                                   sex_padj < 0.1 & sex_log2FoldChange < 0 ~ "Up in females; FDR < 0.1",
                                   sex_padj < 0.3 & sex_log2FoldChange < 0 ~ "Up in females; 0.3 < FDR < 0.1",
                                   sex_padj < 0.5 & sex_log2FoldChange < 0 ~ "Up in females; 0.5 < FDR < 0.3",
                                   sex_padj > 0.5 ~ "FDR > 0.5")) %>%
    mutate(sex_sig_cat = factor(sex_sig_cat, levels = c("Up in females; FDR < 0.1", 
                                                        "Up in females; 0.3 < FDR < 0.1", 
                                                        "Up in females; 0.5 < FDR < 0.3",
                                                        "Up in males; 0.5 < FDR < 0.3",
                                                        "Up in males; 0.3 < FDR < 0.1",
                                                        "Up in males; FDR < 0.1",
                                                        "FDR > 0.5")))
  
  ## D2
  covar_d2_mat <- sample_annot_list[[i]] %>%
    filter(timepoint == "D2") %>%
    mutate(sex = factor(sex),
           case = as.factor(as.numeric(Phenotype == "ME/CFS")),
           bmi_cat = as.factor(bmi_binned)) %>%
    column_to_rownames("sample_id") %>%
    dplyr::select(sex, case, bmi_cat)
  
  ## Case/control
  deseq2_d2_case_dat <- DESeqDataSetFromMatrix(countData = raw_list[[i]][cluster_gene_sets[[i]], rownames(covar_d2_mat)],
                                               colData = covar_d2_mat,
                                               design = ~ sex + bmi_cat + case)
  deseq2_d2_case_results <- DESeq(deseq2_d2_case_dat, minReplicatesForReplace = Inf)
  
  ## Sex 
  deseq2_d2_sex_dat <- DESeqDataSetFromMatrix(countData = raw_list[[i]][cluster_gene_sets[[i]], rownames(covar_d2_mat)],
                                              colData = covar_d2_mat,
                                              design = ~ case + bmi_cat + sex)
  deseq2_d2_sex_results <- DESeq(deseq2_d2_sex_dat, minReplicatesForReplace = Inf)
  
  ## Combine results
  deseq2_d2_results_dat <- results(deseq2_d2_case_results) %>%
    as.data.frame %>%
    rename_with(~ paste("case", .x, sep = "_"), .cols = -baseMean) %>%
    rownames_to_column("symbol") %>%
    left_join(results(deseq2_d2_sex_results) %>%
                as.data.frame %>%
                dplyr::select(-baseMean) %>%
                rename_with(~ paste("sex", .x, sep = "_"), .cols = everything()) %>%
                rownames_to_column("symbol")) %>%
    arrange(case_padj) %>%
    mutate(case_sig_cat = case_when(case_padj < 0.1 & case_log2FoldChange > 0 ~ "Up in cases; FDR < 0.1",
                                    case_padj < 0.3 & case_log2FoldChange > 0 ~ "Up in cases; 0.3 < FDR < 0.1",
                                    case_padj < 0.5 & case_log2FoldChange > 0 ~ "Up in cases; 0.5 < FDR < 0.3",
                                    case_padj < 0.1 & case_log2FoldChange < 0 ~ "Down in cases; FDR < 0.1",
                                    case_padj < 0.3 & case_log2FoldChange < 0 ~ "Down in cases; 0.3 < FDR < 0.1",
                                    case_padj < 0.5 & case_log2FoldChange < 0 ~ "Down in cases; 0.5 < FDR < 0.3",
                                    case_padj > 0.5 ~ "FDR > 0.5")) %>%
    mutate(case_sig_cat = factor(case_sig_cat, levels = c("Down in cases; FDR < 0.1", 
                                                          "Down in cases; 0.3 < FDR < 0.1", 
                                                          "Down in cases; 0.5 < FDR < 0.3",
                                                          "Up in cases; 0.5 < FDR < 0.3",
                                                          "Up in cases; 0.3 < FDR < 0.1",
                                                          "Up in cases; FDR < 0.1",
                                                          "FDR > 0.5"))) %>%
    mutate(sex_sig_cat = case_when(sex_padj < 0.1 & sex_log2FoldChange > 0 ~ "Up in males; FDR < 0.1",
                                   sex_padj < 0.3 & sex_log2FoldChange > 0 ~ "Up in males; 0.3 < FDR < 0.1",
                                   sex_padj < 0.5 & sex_log2FoldChange > 0 ~ "Up in males; 0.5 < FDR < 0.3",
                                   sex_padj < 0.1 & sex_log2FoldChange < 0 ~ "Up in females; FDR < 0.1",
                                   sex_padj < 0.3 & sex_log2FoldChange < 0 ~ "Up in females; 0.3 < FDR < 0.1",
                                   sex_padj < 0.5 & sex_log2FoldChange < 0 ~ "Up in females; 0.5 < FDR < 0.3",
                                   sex_padj > 0.5 ~ "FDR > 0.5")) %>%
    mutate(sex_sig_cat = factor(sex_sig_cat, levels = c("Up in females; FDR < 0.1", 
                                                        "Up in females; 0.3 < FDR < 0.1", 
                                                        "Up in females; 0.5 < FDR < 0.3",
                                                        "Up in males; 0.5 < FDR < 0.3",
                                                        "Up in males; 0.3 < FDR < 0.1",
                                                        "Up in males; FDR < 0.1",
                                                        "FDR > 0.5")))
  
  these_results <- list(D1 = deseq2_d1_results_dat,
                        D2 = deseq2_d2_results_dat)
  pseudo_results[[i]] <- these_results
}
names(pseudo_results) <- analyze_cluster_names
```

##### Sex DEGs

Sex explains a large amount of the variation in the
`Cluster0: naive_cd4` data, with PC2 largely separating based
on sex.

```
ggplot(data = pseudo_results$`cluster0: naive_cd4`$D1,
       aes(x = sex_log2FoldChange, y = -log10(sex_pvalue))) +
  geom_point() +
  geom_label_repel(data = pseudo_results$`cluster0: naive_cd4`$D1 %>% filter(-log10(sex_pvalue) > 100),
                   aes(label = symbol)) +
  xlab("Sex LFC") + ylab("-log10(Sex p-value)") +
  ggtitle("Cluster0: D1 naive_cd4") +
  theme_bw()
```

These DEGs confirm that sex-linked genes are powering this variation,
with X-linked genes like *XIST* and Y-linked genes like
*PRKY* and *DDX3Y*.

Observation of sex differences speaks to the quality of the data, but
we are primarily interested in differences between ME/CFS cases and
controls.

##### Case/control status DEGs

###### Increased in cases

```
## Define sets
# Up in cases
case_Up_fdr0.1_sets <- lapply(pseudo_results, function(x) x$D1 %>% filter(case_padj < 0.1 & case_log2FoldChange > 0) %>% pull(symbol))
case_Up_fdr0.3_sets <- lapply(pseudo_results, function(x) x$D1 %>% filter(case_padj < 0.3 & case_log2FoldChange > 0) %>% pull(symbol))
case_Up_fdr0.5_sets <- lapply(pseudo_results, function(x) x$D1 %>% filter(case_padj < 0.5 & case_log2FoldChange > 0) %>% pull(symbol))

## Define Upset data
case_Up_fdr0.1_upset_dat <- list_cbind(lapply(case_Up_fdr0.1_sets, function(x) as.data.frame(as.numeric(all_genes %in% x)))) %>%
  as.matrix %>%
  as.data.frame
case_Up_fdr0.3_upset_dat <- list_cbind(lapply(case_Up_fdr0.3_sets, function(x) as.data.frame(as.numeric(all_genes %in% x)))) %>%
  as.matrix %>%
  as.data.frame
case_Up_fdr0.5_upset_dat <- list_cbind(lapply(case_Up_fdr0.5_sets, function(x) as.data.frame(as.numeric(all_genes %in% x)))) %>%
  as.matrix %>%
  as.data.frame

names(case_Up_fdr0.1_upset_dat) <- names(case_Up_fdr0.1_sets)
rownames(case_Up_fdr0.1_upset_dat) <- all_genes
names(case_Up_fdr0.3_upset_dat) <- names(case_Up_fdr0.3_sets)
rownames(case_Up_fdr0.3_upset_dat) <- all_genes
names(case_Up_fdr0.5_upset_dat) <- names(case_Up_fdr0.5_sets)
rownames(case_Up_fdr0.5_upset_dat) <- all_genes

# Upset plots
upset(data = case_Up_fdr0.1_upset_dat,
      order.by = "freq",
      nintersects = NA,
      main.bar.color = "tomato4",
      text.scale = 2,
      nsets = sum(colSums(case_Up_fdr0.1_upset_dat) > 0))
```

```
upset(data = case_Up_fdr0.3_upset_dat,
      order.by = "freq",
      nintersects = NA,
      main.bar.color = "tomato3",
      text.scale = 1.5,
      nsets = sum(colSums(case_Up_fdr0.3_upset_dat) > 0))
```

```
upset(data = case_Up_fdr0.5_upset_dat,
      order.by = "freq",
      nintersects = NA,
      main.bar.color = "tomato1",
      text.scale = 1,
      nsets = sum(colSums(case_Up_fdr0.5_upset_dat) > 0))
```

###### Decreased in cases

```
## Define sets
# Down in cases
case_Down_fdr0.1_sets <- lapply(pseudo_results, function(x) x$D1 %>% filter(case_padj < 0.1 & case_log2FoldChange < 0) %>% pull(symbol))
case_Down_fdr0.3_sets <- lapply(pseudo_results, function(x) x$D1 %>% filter(case_padj < 0.3 & case_log2FoldChange < 0) %>% pull(symbol))
case_Down_fdr0.5_sets <- lapply(pseudo_results, function(x) x$D1 %>% filter(case_padj < 0.5 & case_log2FoldChange < 0) %>% pull(symbol))

## Define Upset data
case_Down_fdr0.1_upset_dat <- list_cbind(lapply(case_Down_fdr0.1_sets, function(x) as.data.frame(as.numeric(all_genes %in% x)))) %>%
  as.matrix %>%
  as.data.frame
case_Down_fdr0.3_upset_dat <- list_cbind(lapply(case_Down_fdr0.3_sets, function(x) as.data.frame(as.numeric(all_genes %in% x)))) %>%
  as.matrix %>%
  as.data.frame
case_Down_fdr0.5_upset_dat <- list_cbind(lapply(case_Down_fdr0.5_sets, function(x) as.data.frame(as.numeric(all_genes %in% x)))) %>%
  as.matrix %>%
  as.data.frame

names(case_Down_fdr0.1_upset_dat) <- names(case_Down_fdr0.1_sets)
rownames(case_Down_fdr0.1_upset_dat) <- all_genes
names(case_Down_fdr0.3_upset_dat) <- names(case_Down_fdr0.3_sets)
rownames(case_Down_fdr0.3_upset_dat) <- all_genes
names(case_Down_fdr0.5_upset_dat) <- names(case_Down_fdr0.5_sets)
rownames(case_Down_fdr0.5_upset_dat) <- all_genes

# Upset plots
upset(data = case_Down_fdr0.1_upset_dat,
      order.by = "freq",
      nintersects = NA,
      main.bar.color = "dodgerblue4",
      text.scale = 2,
      nsets = sum(colSums(case_Down_fdr0.1_upset_dat) > 0))
```

```
upset(data = case_Down_fdr0.3_upset_dat,
      order.by = "freq",
      nintersects = NA,
      main.bar.color = "dodgerblue3",
      text.scale = 2,
      nsets = sum(colSums(case_Down_fdr0.3_upset_dat) > 0))
```

```
upset(data = case_Down_fdr0.5_upset_dat,
      order.by = "freq",
      nintersects = NA,
      main.bar.color = "dodgerblue1",
      text.scale = 1.5,
      nsets = sum(colSums(case_Down_fdr0.5_upset_dat) > 0))
```

###### Mitochondrial DEGs in platelets

`Cluster19: platelet` stands out, having the most DEGs in
both directions.

```
mtco1_plot_dat <- norm_list$`cluster19: platelet` %>%
  rownames_to_column("gene_name") %>%
  pivot_longer(-gene_name, names_to = "sample_id", values_to = "count") %>%
  mutate(cluster = "cluster19: platelet") %>%
  left_join(sample_annot_list$`cluster19: platelet`) %>%
  filter(gene_name %in% case_Up_fdr0.1_sets$`cluster19: platelet`) %>%
  mutate(case = ifelse(Phenotype == "ME/CFS", "case", "control"))

ggplot(data = mtco1_plot_dat %>%
         filter(timepoint == "D1"),
       aes(x = case, y = count)) +
  geom_boxplot(fill = "white", col = "black") +
  geom_beeswarm(aes(col = case)) +
  xlab("Case/control status") + ylab("Normalized gene count") +
  labs(col = "Case/control status") +
  theme_bw() +
  facet_wrap(~gene_name, nrow = 2, scales = "free_y")
```

Seven mitochondria-encoded genes appear to have elevated expression
in platelets.

```
## Combine results
combined_pseudo_results_dat <- NULL
for (i in 1:length(pseudo_results)) {
  combined_pseudo_results_dat <- bind_rows(combined_pseudo_results_dat,
                                           pseudo_results[[i]]$D1 %>%
                                             mutate(cluster = names(pseudo_results)[i]))
}
combined_pseudo_results_dat <- combined_pseudo_results_dat %>%
  mutate(cluster = factor(cluster, levels = cluster_names[1:28]))

mt_genes <- grep(x = case_Up_fdr0.1_sets$`cluster19: platelet`, pattern = "MT-", value = TRUE)

mt_results <- combined_pseudo_results_dat %>%
  filter(symbol %in% mt_genes) %>%
  mutate(cluster_num = gsub(x = cluster, pattern = "cluster", replacement = "")) %>%
  mutate(cluster_num = gsub(x = cluster_num, pattern = ":.+$", perl = TRUE, replacement = ""))

ggplot(data = mt_results,
       aes(x = case_log2FoldChange, y = -log10(case_pvalue), col = cluster)) +
  geom_text(aes(label = cluster_num)) +
  geom_vline(xintercept = 0, linetype = "dashed") +
  xlab("Case/control LFC") + ylab("-Log10(case/control p-value)") +
  theme_bw() +
  theme(legend.position = "bottom") +
  facet_wrap(~symbol)
```

Though these DEGs are primarily detected in platelets, we see similar
direction of effects in the other clusters.

We will now produce the same visuals as we did for the bulk data
results, including the extent of null signal, volcano plots, DEG counts,
and gene tables.

##### P-value distributions

```
## Histogram of p-values
ggplot(data = combined_pseudo_results_dat,
       aes(x = case_pvalue)) +
  geom_histogram(fill = "gray", col = "black") +
  xlab("Case/control p-value") +
  theme_bw() +
  facet_wrap(~cluster)
```

```
pseudo_d1_results <- list()
for (i in 1:length(pseudo_results)) {
  pseudo_d1_results[[i]] <- pseudo_results[[i]]$D1
}
names(pseudo_d1_results) <- names(pseudo_results)

p0_dat <- data.frame(cluster = names(pseudo_d1_results),
                     p0 = sapply(1:length(pseudo_d1_results), function(i) get.pi0(pvalues = pseudo_d1_results[[i]]$case_pvalue))) %>%
  mutate(cluster = factor(cluster, levels = cluster_names[1:28]))

ggplot(data = p0_dat,
       aes(x = cluster, y = p0, fill = cluster)) + 
  geom_bar(stat = "identity") +
  geom_hline(yintercept = 1, linetype = "dashed") +
  guides(fill = "none") +
  xlab("") +
  theme_bw() +
  theme(axis.text.x = element_text(angle = 60, hjust = 1, vjust = 1))
```

```
p0_dat
```

```
##                         cluster        p0
## 1           cluster0: naive_cd4 1.0000000
## 2         cluster1: eff_mem_cd4 1.0000000
## 3      cluster2: class_monocyte 1.0000000
## 4            cluster3: cytox_nk 0.9715047
## 5         cluster4: eff_mem_cd8 1.0000000
## 6       cluster5: eff_mem_cd8_2 1.0000000
## 7           cluster6: naive_cd8 1.0000000
## 8                   cluster7: B 0.9673086
## 9                 cluster8: B_2 1.0000000
## 10  cluster9: nonclass_monocyte 1.0000000
## 11 cluster10: intermed_monocyte 1.0000000
## 12              cluster11: treg 1.0000000
## 13              cluster12: mait 1.0000000
## 14               cluster13: gdT 1.0000000
## 15                cluster14: DC 1.0000000
## 16            cluster15: reg_nk 1.0000000
## 17                 cluster16: T 1.0000000
## 18         cluster17: monocytes 1.0000000
## 19               cluster18: pDC 1.0000000
## 20          cluster19: platelet 0.6367384
## 21      cluster20: prolif_cells 1.0000000
## 22       cluster21: monocytes_2 0.9631728
## 23       cluster22: plasmablast 1.0000000
## 24               cluster23: T_2 1.0000000
## 25               cluster24: HSC 0.9170531
## 26             cluster25: gdT_2 1.0000000
## 27       cluster26: monocytes_3 1.0000000
## 28               cluster27: B_3 1.0000000
```

We observed support for non-null signal in five of the clusters, with
the largest levels in `cluster19: platelet` unsurprisingly
given the counts of DEGs.

##### Volcano plots

```
combined_pseudo_results_dat <- combined_pseudo_results_dat %>%
  mutate(case_sig = case_padj < 0.1) %>%
  filter(!is.na(case_padj))  

## Histogram of p-values
ggplot(data = combined_pseudo_results_dat,
       aes(x = case_log2FoldChange, y = -log10(case_pvalue))) +
  geom_point(aes(col = case_sig)) +
  geom_label_repel(data = combined_pseudo_results_dat %>% 
                     filter(case_padj < 0.1),
                   aes(label = symbol),
                   size = 2) +
  xlab("Case/control LFC") + ylab("-Log10(case/control p-value)") +
  scale_color_manual("FDR < 0.1", values = c("gray", "red")) +
  theme_bw() +
  theme(legend.position = "bottom") +
  facet_wrap(~cluster, nrow = 4)
```

##### Differentially expressed gene counts

```
deg_count_dat <- combined_pseudo_results_dat %>%
  filter(!is.na(case_sig_cat)) %>%
  filter(case_sig_cat != "FDR > 0.5") %>%
  group_by(cluster, case_sig_cat) %>%
  tally

ggplot(data = deg_count_dat,
       aes(x = case_sig_cat, y = n, fill = case_sig_cat)) +
  geom_bar(stat = "identity") +
  geom_text(data = deg_count_dat,
            aes(y = n + max(n)*0.085, label = n), size = 6) +
  scale_fill_manual("FDR < 0.1", values = c("dodgerblue4", "dodgerblue3", "dodgerblue1",
                                            "tomato1", "tomato3", "tomato4")) +
  guides(fill = "none") +
  xlab("") + ylab("count") +
  theme_bw() +
  theme(axis.text.x = element_text(size = 12, angle = 60, hjust = 1),
        axis.title.x = element_text(size = 12),
        axis.text.y = element_text(size = 12),
        axis.title.y = element_text(size = 12),
        legend.position = "bottom") +
  facet_wrap(~ cluster)
```

#### Differential gene expression tables

```
deseq2_sig_cat <- combined_pseudo_results_dat %>%
  filter(case_padj < 0.5) %>%
  dplyr::select(case_sig_cat) %>%
  distinct %>%
  filter(!is.na(case_sig_cat)) %>%
  pull(case_sig_cat) %>%
  as.character

deseq2_results_table <- combined_pseudo_results_dat %>%
      filter(!is.na(case_padj)) %>%
      dplyr::select(symbol, cluster, baseMean, case_log2FoldChange, case_lfcSE, case_pvalue, case_padj, case_sig_cat) %>%
      dplyr::rename(log2FoldChange = case_log2FoldChange,
                    lfcSE = case_lfcSE,
                    pvalue = case_pvalue,
                    padj = case_padj,
                    sig_cat = case_sig_cat) %>%
      filter(padj < 0.5) %>%
      arrange(padj) %>% 
      mutate(baseMean = round(baseMean, 3),
             log2FoldChange = round(log2FoldChange, 3),
             lfcSE = round(lfcSE, 3),
             pvalue = round(pvalue, 7),
             padj = round(padj, 3),
             sig_cat = as.character(sig_cat))

## Redefining colors because of weird bug in DT color handling
# Can't seem to handle colors with integer at end of word (e.g., "dodgerblue4")
dodgerblue4_new <- col2rgb("dodgerblue4")[,1]
dodgerblue4_new <- rgb(dodgerblue4_new[1], dodgerblue4_new[2], dodgerblue4_new[3], maxColorValue = 255)
dodgerblue3_new <- col2rgb("dodgerblue3")[,1]
dodgerblue3_new <- rgb(dodgerblue3_new[1], dodgerblue3_new[2], dodgerblue3_new[3], maxColorValue = 255)
dodgerblue1_new <- col2rgb("dodgerblue1")[,1]
dodgerblue1_new <- rgb(dodgerblue1_new[1], dodgerblue1_new[2], dodgerblue1_new[3], maxColorValue = 255)
tomato4_new <- col2rgb("tomato4")[,1]
tomato4_new <- rgb(tomato4_new[1], tomato4_new[2], tomato4_new[3], maxColorValue = 255)
tomato3_new <- col2rgb("tomato3")[,1]
tomato3_new <- rgb(tomato3_new[1], tomato3_new[2], tomato3_new[3], maxColorValue = 255)
tomato1_new <- col2rgb("tomato1")[,1]
tomato1_new <- rgb(tomato1_new[1], tomato1_new[2], tomato1_new[3], maxColorValue = 255)

htmltools::tagList(
      datatable(deseq2_results_table,
                caption = "Differential gene expression results from pseudobulk data derived from PBMC from Vu et al. 2024") %>%
      formatStyle("log2FoldChange", "sig_cat", 
                  backgroundColor = styleEqual(deseq2_sig_cat, c(dodgerblue4_new, tomato4_new, 
                                                                 dodgerblue3_new, tomato3_new,
                                                                 dodgerblue1_new, tomato1_new)),
                  color = "white"))
```

## Gamer *et al.* 2023 bulk RNA-seq in PBMCs

### Introduction

These data were published as part of Gamer *et al.*
2023 and Van Booven
*et al.* 2023, both published in the *International
Journal of Molecular Sciences*. We primarily focus on Gamer *et
al.* 2023 as the more recent study, which includes the female-only
cohort from Van Booven *et al* 2023 and additional male
participants. Both studies were focused on post-exertional malaise (PEM)
in ME/CFS cases. Subjects underwent an exercise challenge and PBMC
samples collected at three different timepoints: baseline before
exercise (T0), at maximal exertion (T1), and four hours after maximal
exertion (T2). We will focus on T0 results when comparing to other
studies.

| Study | Data Type | Tissue | Case/Control | Notes |
| --- | --- | --- | --- | --- |
| Gamer *et al.* 2023 | Gene expression (bulk RNA-seq) | PBMC | 28/30 (for T0) | Females and males |

---

### Analysis

#### Overview

Gamer *et al.*
2023 and Van Booven
*et al.* 2019 performed the differential expression analysis
using `DESeq2`. They filtering out genes from the X and Y
chromosomes and batch correction through ComBat-seq. They further
filtered genes based on requiring gene counts of 50 or higher in all
samples in at least one group (*e.g.*, ME/CFS cases at T0). They
used three different aligners (GSNAP, HISAT2, and STAR). They performed
differential expression analysis for count data from each aligner. They
do not describe the covariates they included in the model. They defined
differentially expressed genes across the different aligners based on
two criteria:

> 1. either a fold change (FC) > 1.5 and false discovery rate (FDR)
>    < 0.10 in one out of three aligners, and FC > 1.4 and FDR <
>    0.15 in the other two aligners; or
> 2. an FC > 1.5 and FDR < 0.10 in at least two out of three
>    aligners.

We will use a simplified approach based on HISAT2-aligned data
because it resulted in fewer genes with outlying data points. We note
that all aligners resulted in generally consistent differentially
expressed gene results. We include sex as a covariate in the model.

#### Load data

```
## Set to the appropriate directory in your environment
setwd("<Your Directory>")
```

```
## Important factors and covariates (e.g., case/control, sex)
sample_annot <- read.table("data/gamer_sample_annot.tsv", header = TRUE, sep = "\t")

## Split into timepoint-specific annotations
sample_annot_t0 <- sample_annot %>%
  filter(timepoint == "T0") %>%
  dplyr::select(-timepoint)
sample_annot_t1 <- sample_annot %>%
  filter(timepoint == "T1") %>%
  dplyr::select(-timepoint)
sample_annot_t2 <- sample_annot %>%
  filter(timepoint == "T2") %>%
  dplyr::select(-timepoint)

## Gene annotations from biomart
gene_annot <- read.table("data/gene_annot_from_biomart_20241001.tsv", 
                         sep = "\t", header = TRUE)

## Gene count data
gene_count_dat <- read.table("data/gamer_pbmc_tx.tsv",
                             header = TRUE, sep = "\t") %>%
  left_join(gene_annot) %>%
  dplyr::rename(symbol = Gene,
                gene_id = Molecule)
```

#### Prepare data for `DESeq2`

```
## Convert covariates to a matrix with ParticipantID as rownames
covar_mat_t0 <- sample_annot_t0 %>%
  mutate(Sex = ifelse(Sex == "Female", 1, 0)) %>%
  dplyr::select(ParticipantID, case, Sex) %>%
  column_to_rownames("ParticipantID") %>%
  mutate(case = factor(case),
         Sex = factor(Sex))
covar_mat_t1 <- sample_annot_t1 %>%
  mutate(Sex = ifelse(Sex == "Female", 1, 0)) %>%
  dplyr::select(ParticipantID, case, Sex) %>%
  column_to_rownames("ParticipantID") %>%
  mutate(case = factor(case),
         Sex = factor(Sex))
covar_mat_t2 <- sample_annot_t2 %>%
  mutate(Sex = ifelse(Sex == "Female", 1, 0)) %>%
  dplyr::select(ParticipantID, case, Sex) %>%
  column_to_rownames("ParticipantID") %>%
  mutate(case = factor(case),
         Sex = factor(Sex))

## Convert gene counts to a matrix with ENSEMBL gene IDs as rownames and ParticipantID as colnames
gene_mat <- gene_count_dat %>%
  dplyr::select(-symbol) %>%
  column_to_rownames("gene_id") %>%
  apply(., 2, function(x) round(x))

## Preparing DESeq2 data structure for analysis
deseq2_count_t0_dat <- DESeqDataSetFromMatrix(countData = gene_mat[,rownames(covar_mat_t0)],
                                              colData = covar_mat_t0,
                                              design = ~ Sex + case)
deseq2_count_t1_dat <- DESeqDataSetFromMatrix(countData = gene_mat[,rownames(covar_mat_t1)],
                                              colData = covar_mat_t1,
                                              design = ~ Sex + case)
deseq2_count_t2_dat <- DESeqDataSetFromMatrix(countData = gene_mat[,rownames(covar_mat_t2)],
                                              colData = covar_mat_t2,
                                              design = ~ Sex + case)
```

#### Running differential expression analysis testing case/control status

```
## T0
deseq2_t0_results <- DESeq(deseq2_count_t0_dat)

deseq2_results_t0_dat <- results(deseq2_t0_results) %>%
  as.data.frame %>%
  arrange(padj) %>%
  rownames_to_column("gene_id") %>%
  ## Merge in gene symbol information
  left_join(gene_count_dat %>%
              dplyr::select(gene_id, symbol)) %>%
  mutate(sig_cat = case_when(padj < 0.1 & log2FoldChange > 0 ~ "Up in cases; FDR < 0.1",
                             padj < 0.3 & log2FoldChange > 0 ~ "Up in cases; 0.3 < FDR < 0.1",
                             padj < 0.5 & log2FoldChange > 0 ~ "Up in cases; 0.5 < FDR < 0.3",
                             padj < 0.1 & log2FoldChange < 0 ~ "Down in cases; FDR < 0.1",
                             padj < 0.3 & log2FoldChange < 0 ~ "Down in cases; 0.3 < FDR < 0.1",
                             padj < 0.5 & log2FoldChange < 0 ~ "Down in cases; 0.5 < FDR < 0.3",
                             padj > 0.5 ~ "FDR > 0.5")) %>%
  mutate(sig_cat = factor(sig_cat, levels = c("Down in cases; FDR < 0.1", 
                                              "Down in cases; 0.3 < FDR < 0.1", 
                                              "Down in cases; 0.5 < FDR < 0.3",
                                              "Up in cases; 0.5 < FDR < 0.3",
                                              "Up in cases; 0.3 < FDR < 0.1",
                                              "Up in cases; FDR < 0.1",
                                              "FDR > 0.5")))

## T1
deseq2_t1_results <- DESeq(deseq2_count_t1_dat)

deseq2_results_t1_dat <- results(deseq2_t1_results) %>%
  as.data.frame %>%
  arrange(padj) %>%
  rownames_to_column("gene_id") %>%
  ## Merge in gene symbol information
  left_join(gene_count_dat %>%
              dplyr::select(gene_id, symbol)) %>%
  mutate(sig_cat = case_when(padj < 0.1 & log2FoldChange > 0 ~ "Up in cases; FDR < 0.1",
                             padj < 0.3 & log2FoldChange > 0 ~ "Up in cases; 0.3 < FDR < 0.1",
                             padj < 0.5 & log2FoldChange > 0 ~ "Up in cases; 0.5 < FDR < 0.3",
                             padj < 0.1 & log2FoldChange < 0 ~ "Down in cases; FDR < 0.1",
                             padj < 0.3 & log2FoldChange < 0 ~ "Down in cases; 0.3 < FDR < 0.1",
                             padj < 0.5 & log2FoldChange < 0 ~ "Down in cases; 0.5 < FDR < 0.3",
                             padj > 0.5 ~ "FDR > 0.5")) %>%
  mutate(sig_cat = factor(sig_cat, levels = c("Down in cases; FDR < 0.1", 
                                              "Down in cases; 0.3 < FDR < 0.1", 
                                              "Down in cases; 0.5 < FDR < 0.3",
                                              "Up in cases; 0.5 < FDR < 0.3",
                                              "Up in cases; 0.3 < FDR < 0.1",
                                              "Up in cases; FDR < 0.1",
                                              "FDR > 0.5")))

## T2
deseq2_t2_results <- DESeq(deseq2_count_t2_dat)

deseq2_results_t2_dat <- results(deseq2_t2_results) %>%
  as.data.frame %>%
  arrange(padj) %>%
  rownames_to_column("gene_id") %>%
  ## Merge in gene symbol information
  left_join(gene_count_dat %>%
              dplyr::select(gene_id, symbol)) %>%
  mutate(sig_cat = case_when(padj < 0.1 & log2FoldChange > 0 ~ "Up in cases; FDR < 0.1",
                             padj < 0.3 & log2FoldChange > 0 ~ "Up in cases; 0.3 < FDR < 0.1",
                             padj < 0.5 & log2FoldChange > 0 ~ "Up in cases; 0.5 < FDR < 0.3",
                             padj < 0.1 & log2FoldChange < 0 ~ "Down in cases; FDR < 0.1",
                             padj < 0.3 & log2FoldChange < 0 ~ "Down in cases; 0.3 < FDR < 0.1",
                             padj < 0.5 & log2FoldChange < 0 ~ "Down in cases; 0.5 < FDR < 0.3",
                             padj > 0.5 ~ "FDR > 0.5")) %>%
  mutate(sig_cat = factor(sig_cat, levels = c("Down in cases; FDR < 0.1", 
                                              "Down in cases; 0.3 < FDR < 0.1", 
                                              "Down in cases; 0.5 < FDR < 0.3",
                                              "Up in cases; 0.5 < FDR < 0.3",
                                              "Up in cases; 0.3 < FDR < 0.1",
                                              "Up in cases; FDR < 0.1",
                                              "FDR > 0.5")))
```

---

### Results

#### T0

##### P-value distribution

```
## Histogram of p-values
ggplot(data = deseq2_results_t0_dat,
       aes(x = pvalue)) +
  geom_histogram(fill = "gray", col = "black") +
  theme_bw()
```

```
## Estimate proportion of p-values that follow the null distribution (Uniform)
get.pi0(pvalues = deseq2_results_t0_dat$pvalue)
```

```
## [1] 1
```

The p-value distribution is not ideal for T0. Nevertheless, we will
look at the differentially expressed genes and look for consistency and
differences across timepoints.

##### Volcano plot

```
ggplot(data = deseq2_results_t0_dat %>% filter(!is.na(padj)),
       aes(x = log2FoldChange, y = -log10(pvalue), col = sig_cat)) +
  geom_point(size = 2) +
  geom_text_repel(data = deseq2_results_t0_dat %>%
                    filter(padj < 0.1),
                  aes(label = symbol), col = "black") +
  scale_color_manual("FDR < 0.1", values = c("dodgerblue4", "dodgerblue3", "dodgerblue1", 
                                             "tomato1", "tomato3", "tomato4",
                                             "gray"),
                     drop = FALSE) +
  xlab("Log2 Fold Change") + ylab("-log10P") +
  theme_bw() +
  theme(axis.text.x = element_text(size = 12),
        axis.title.x = element_text(size = 12),
        axis.text.y = element_text(size = 12),
        axis.title.y = element_text(size = 12),
        legend.position = "bottom")
```

##### Differentially expressed gene counts

```
deg_count_t0_dat <- deseq2_results_t0_dat %>%
  filter(!is.na(sig_cat)) %>%
  filter(sig_cat != "FDR > 0.5") %>%
  group_by(sig_cat) %>%
  tally

ggplot(data = deg_count_t0_dat,
       aes(x = sig_cat, y = n, fill = sig_cat)) +
  geom_bar(stat = "identity") +
  geom_text(data = deg_count_t0_dat,
            aes(y = n + max(n)*0.05, label = n), size = 8) +
  scale_fill_manual("FDR < 0.1", values = c("dodgerblue4", "dodgerblue3", "dodgerblue1",
                                            "tomato1", "tomato3", "tomato4")) +
  guides(fill = "none") +
  xlab("") + ylab("count") +
  theme_bw() +
  theme(axis.text.x = element_text(size = 12, angle = 45, hjust = 1),
        axis.title.x = element_text(size = 12),
        axis.text.y = element_text(size = 12),
        axis.title.y = element_text(size = 12),
        legend.position = "bottom")
```

##### Differential gene expression tables

```
deseq2_sig_cat <- deseq2_results_t0_dat %>%
  filter(padj < 0.5) %>%
  dplyr::select(sig_cat) %>%
  distinct %>%
  filter(!is.na(sig_cat)) %>%
  pull(sig_cat) %>%
  as.character

deseq2_results_t0_table <- deseq2_results_t0_dat %>%
      filter(!is.na(padj)) %>%
      dplyr::select(gene_id, symbol, baseMean, log2FoldChange, lfcSE, pvalue, padj, sig_cat) %>%
      filter(padj < 0.5) %>%
      arrange(padj) %>% 
      mutate(baseMean = round(baseMean, 3),
             log2FoldChange = round(log2FoldChange, 3),
             lfcSE = round(lfcSE, 3),
             pvalue = round(pvalue, 7),
             padj = round(padj, 3),
             sig_cat = as.character(sig_cat))

## Redefining colors because of weird bug in DT color handling
# Can't seem to handle colors with integer at end of word (e.g., "dodgerblue4")
dodgerblue4_new <- col2rgb("dodgerblue4")[,1]
dodgerblue4_new <- rgb(dodgerblue4_new[1], dodgerblue4_new[2], dodgerblue4_new[3], maxColorValue = 255)
dodgerblue3_new <- col2rgb("dodgerblue3")[,1]
dodgerblue3_new <- rgb(dodgerblue3_new[1], dodgerblue3_new[2], dodgerblue3_new[3], maxColorValue = 255)
dodgerblue1_new <- col2rgb("dodgerblue1")[,1]
dodgerblue1_new <- rgb(dodgerblue1_new[1], dodgerblue1_new[2], dodgerblue1_new[3], maxColorValue = 255)
tomato4_new <- col2rgb("tomato4")[,1]
tomato4_new <- rgb(tomato4_new[1], tomato4_new[2], tomato4_new[3], maxColorValue = 255)
tomato3_new <- col2rgb("tomato3")[,1]
tomato3_new <- rgb(tomato3_new[1], tomato3_new[2], tomato3_new[3], maxColorValue = 255)
tomato1_new <- col2rgb("tomato1")[,1]
tomato1_new <- rgb(tomato1_new[1], tomato1_new[2], tomato1_new[3], maxColorValue = 255)

htmltools::tagList(
      datatable(deseq2_results_t0_table,
                caption = "Differential gene expression results in PBMC data at T0 from Gamer et al. 2023") %>%
      formatStyle("log2FoldChange", "sig_cat", 
                  backgroundColor = styleEqual(deseq2_sig_cat, c(dodgerblue4_new, tomato4_new, 
                                                                 dodgerblue3_new, tomato3_new,
                                                                 dodgerblue1_new, tomato1_new)),
                  color = "white"))
```

#### T1

##### P-value distribution

```
## Histogram of p-values
ggplot(data = deseq2_results_t1_dat,
       aes(x = pvalue)) +
  geom_histogram(fill = "gray", col = "black") +
  theme_bw()
```

```
## Estimate proportion of p-values that follow the null distribution (Uniform)
get.pi0(pvalues = deseq2_results_t1_dat$pvalue)
```

```
## [1] 1
```

##### Volcano plot

```
ggplot(data = deseq2_results_t1_dat %>% filter(!is.na(padj)),
       aes(x = log2FoldChange, y = -log10(pvalue), col = sig_cat)) +
  geom_point(size = 2) +
  geom_text_repel(data = deseq2_results_t1_dat %>%
                    filter(padj < 0.1),
                  aes(label = symbol), col = "black") +
  scale_color_manual("FDR < 0.1", values = c("dodgerblue4", "dodgerblue3", "dodgerblue1", 
                                             "tomato1", "tomato3", "tomato4",
                                             "gray"),
                     drop = FALSE) +
  xlab("Log2 Fold Change") + ylab("-log10P") +
  theme_bw() +
  theme(axis.text.x = element_text(size = 12),
        axis.title.x = element_text(size = 12),
        axis.text.y = element_text(size = 12),
        axis.title.y = element_text(size = 12),
        legend.position = "bottom")
```

##### Differentially expressed gene counts

```
deg_count_t1_dat <- deseq2_results_t1_dat %>%
  filter(!is.na(sig_cat)) %>%
  filter(sig_cat != "FDR > 0.5") %>%
  group_by(sig_cat) %>%
  tally

ggplot(data = deg_count_t1_dat,
       aes(x = sig_cat, y = n, fill = sig_cat)) +
  geom_bar(stat = "identity") +
  geom_text(data = deg_count_t1_dat,
            aes(y = n + max(n)*0.05, label = n), size = 8) +
  scale_fill_manual("FDR < 0.1", values = c("dodgerblue4", "dodgerblue3", "dodgerblue1",
                                            "tomato1", "tomato3", "tomato4")) +
  guides(fill = "none") +
  xlab("") + ylab("count") +
  theme_bw() +
  theme(axis.text.x = element_text(size = 12, angle = 45, hjust = 1),
        axis.title.x = element_text(size = 12),
        axis.text.y = element_text(size = 12),
        axis.title.y = element_text(size = 12),
        legend.position = "bottom")
```

##### Differential gene expression tables

```
deseq2_results_t1_table <- deseq2_results_t1_dat %>%
      filter(!is.na(padj)) %>%
      dplyr::select(gene_id, symbol, baseMean, log2FoldChange, lfcSE, pvalue, padj, sig_cat) %>%
      filter(padj < 0.5) %>%
      arrange(padj) %>% 
      mutate(baseMean = round(baseMean, 3),
             log2FoldChange = round(log2FoldChange, 3),
             lfcSE = round(lfcSE, 3),
             pvalue = round(pvalue, 7),
             padj = round(padj, 3),
             sig_cat = as.character(sig_cat))

htmltools::tagList(
      datatable(deseq2_results_t1_table,
                caption = "Differential gene expression results in PBMC data at T1 from Gamer et al. 2023") %>%
      formatStyle("log2FoldChange", "sig_cat", 
                  backgroundColor = styleEqual(deseq2_sig_cat, c(dodgerblue4_new, tomato4_new, 
                                                                 dodgerblue3_new, tomato3_new,
                                                                 dodgerblue1_new, tomato1_new)),
                  color = "white"))
```

#### T2

##### P-value distribution

```
## Histogram of p-values
ggplot(data = deseq2_results_t2_dat,
       aes(x = pvalue)) +
  geom_histogram(fill = "gray", col = "black") +
  theme_bw()
```

```
## Estimate proportion of p-values that follow the null distribution (Uniform)
get.pi0(pvalues = deseq2_results_t2_dat$pvalue)
```

```
## [1] 1
```

The p-value distribution is not ideal for T2. Nevertheless, we will
look at the differentially expressed genes and look for consistency and
differences across timepoints.

##### Volcano plot

```
ggplot(data = deseq2_results_t2_dat %>% filter(!is.na(padj)),
       aes(x = log2FoldChange, y = -log10(pvalue), col = sig_cat)) +
  geom_point(size = 2) +
  geom_text_repel(data = deseq2_results_t2_dat %>%
                    filter(padj < 0.1),
                  aes(label = symbol), col = "black") +
  scale_color_manual("FDR < 0.1", values = c("dodgerblue4", "dodgerblue3", "dodgerblue1", 
                                             "tomato1", "tomato3", "tomato4",
                                             "gray"), 
                     drop = FALSE) +
  xlab("Log2 Fold Change") + ylab("-log10P") +
  theme_bw() +
  theme(axis.text.x = element_text(size = 12),
        axis.title.x = element_text(size = 12),
        axis.text.y = element_text(size = 12),
        axis.title.y = element_text(size = 12),
        legend.position = "bottom")
```

We observe some consistency in differential gene expression across
timepoints. Notably, *MT-RNR1* is detected at T0 and T2 as being
more lowly expressed in ME/CFS cases. At T1, *MT-RNR1* still has
reduced expression in ME/CFS cases, but its significance does not met
FDR < 10%. *MT-RNR2* is also observed in some of the
timepoints. The results are consistent with DEGs in monocytes derived
from PBMC from Raijmakers *et
al.* 2019.

Another result that stands out is that both hemoglobin alpha and beta
are more highly expressed in ME/CFS cases at T1 (maximal exertion).

##### Differentially expressed gene counts

```
deg_count_t2_dat <- deseq2_results_t2_dat %>%
  filter(!is.na(sig_cat)) %>%
  filter(sig_cat != "FDR > 0.5") %>%
  group_by(sig_cat) %>%
  tally

ggplot(data = deg_count_t2_dat,
       aes(x = sig_cat, y = n, fill = sig_cat)) +
  geom_bar(stat = "identity") +
  geom_text(data = deg_count_t2_dat,
            aes(y = n + max(n)*0.05, label = n), size = 8) +
  scale_fill_manual("FDR < 0.1", values = c("dodgerblue4", "dodgerblue3", "dodgerblue1",
                                            "tomato1", "tomato3", "tomato4")) +
  guides(fill = "none") +
  xlab("") + ylab("count") +
  theme_bw() +
  theme(axis.text.x = element_text(size = 12, angle = 45, hjust = 1),
        axis.title.x = element_text(size = 12),
        axis.text.y = element_text(size = 12),
        axis.title.y = element_text(size = 12),
        legend.position = "bottom")
```

##### Differential gene expression tables

```
deseq2_results_t2_table <- deseq2_results_t2_dat %>%
      filter(!is.na(padj)) %>%
      dplyr::select(gene_id, symbol, baseMean, log2FoldChange, lfcSE, pvalue, padj, sig_cat) %>%
      filter(padj < 0.5) %>%
      arrange(padj) %>% 
      mutate(baseMean = round(baseMean, 3),
             log2FoldChange = round(log2FoldChange, 3),
             lfcSE = round(lfcSE, 3),
             pvalue = round(pvalue, 7),
             padj = round(padj, 3),
             sig_cat = as.character(sig_cat))

htmltools::tagList(
      datatable(deseq2_results_t2_table,
                caption = "Differential gene expression results in PBMC data at T2 from Gamer et al. 2023") %>%
      formatStyle("log2FoldChange", "sig_cat", 
                  backgroundColor = styleEqual(deseq2_sig_cat, c(dodgerblue4_new, tomato4_new, 
                                                                 dodgerblue3_new, tomato3_new,
                                                                 dodgerblue1_new, tomato1_new)),
                  color = "white"))
```

## Walitt *et al.* 2024 bulk RNA-seq in PBMCs

### Introduction

These data were published as part of Walitt *et al.*
2024 in *Nature Communications*. This study deeply phenotyped
post-infectious ME/CFS cases, including both gene and protein expression
from multiple tissues, and compared to healthy controls.

| Study | Data Type | Tissue | Case/Control | Notes |
| --- | --- | --- | --- | --- |
| Walitt *et al.* 2024 | Gene expression (bulk RNA-seq) | PBMC | 15/11 | Females and males |

---

### Analysis

#### Overview

Walitt *et
al.* 2024 performed the differential expression analysis using
`limma`. They primarily report results from sex stratified
analyses, which we will avoid due to further reducing a small sample.
Here we use `DESeq2` instead, including sex as a covariate,
as well as BMI, following Walitt *et al.* 2024.

#### Load data

```
## Set to the appropriate directory in your environment
setwd("<Your Directory>")
```

```
## Important factors and covariates (e.g., case/control, sex)
sample_annot <- read.csv("data/walitt_sample_annot.csv", header = TRUE)

## Gene count data
gene_count_dat <- read.table("data/walitt_pbmc_tx.tsv",
                         sep = "\t", header = TRUE) %>%
 dplyr::rename(symbol = Gene,
               gene_id = Molecule)
```

#### Prepare data for `DESeq2`

```
## Convert covariates to a matrix with ParticipantID as rownames
covar_mat <- sample_annot %>%
  dplyr::select(ParticipantID, Group, Birth.Sex, BMI) %>%
  dplyr::rename(case = Group,
                Sex = Birth.Sex) %>%
  mutate(ParticipantID = gsub(x = ParticipantID, pattern = "-", replacement = ".")) %>%
  column_to_rownames("ParticipantID") %>%
  mutate(case = factor(case),
         Sex = factor(Sex),
         BMI = scale(BMI))

## Convert gene counts to a matrix with ENSEMBL gene IDs as rownames and ParticipantID as colnames
gene_mat <- gene_count_dat %>%
  dplyr::select(-symbol) %>%
  column_to_rownames("gene_id")

## Preparing DESeq2 data structure for analysis
deseq2_count_dat <- DESeqDataSetFromMatrix(countData = gene_mat,
                                           colData = covar_mat[colnames(gene_mat),],
                                           design = ~ Sex + BMI + case)
```

#### Running differential expression analysis testing case/control status

```
deseq2_results <- DESeq(deseq2_count_dat)

deseq2_results_dat <- results(deseq2_results) %>%
  as.data.frame %>%
  arrange(padj) %>%
  rownames_to_column("gene_id") %>%
  ## Merge in gene symbol information
  left_join(gene_count_dat %>%
              dplyr::select(gene_id, symbol)) %>%
  mutate(sig_cat = case_when(padj < 0.1 & log2FoldChange > 0 ~ "Up in cases; FDR < 0.1",
                             padj < 0.3 & log2FoldChange > 0 ~ "Up in cases; 0.3 < FDR < 0.1",
                             padj < 0.5 & log2FoldChange > 0 ~ "Up in cases; 0.5 < FDR < 0.3",
                             padj < 0.1 & log2FoldChange < 0 ~ "Down in cases; FDR < 0.1",
                             padj < 0.3 & log2FoldChange < 0 ~ "Down in cases; 0.3 < FDR < 0.1",
                             padj < 0.5 & log2FoldChange < 0 ~ "Down in cases; 0.5 < FDR < 0.3",
                             padj > 0.5 ~ "FDR > 0.5")) %>%
  mutate(sig_cat = factor(sig_cat, levels = c("Down in cases; FDR < 0.1", 
                                              "Down in cases; 0.3 < FDR < 0.1", 
                                              "Down in cases; 0.5 < FDR < 0.3",
                                              "Up in cases; 0.5 < FDR < 0.3",
                                              "Up in cases; 0.3 < FDR < 0.1",
                                              "Up in cases; FDR < 0.1",
                                              "FDR > 0.5")))
```

---

### Results

#### P-value distribution

```
## Histogram of p-values
ggplot(data = deseq2_results_dat,
       aes(x = pvalue)) +
  geom_histogram(fill = "gray", col = "black") +
  theme_bw()
```

```
## Estimate proportion of p-values that follow the null distribution (Uniform)
get.pi0(pvalues = deseq2_results_dat$pvalue)
```

```
## [1] 0.9940661
```

There is some support for differentially expressed genes because the
null proportion is below 100%, though it is very weak.

#### Volcano plot

```
ggplot(data = deseq2_results_dat %>% filter(!is.na(padj)),
       aes(x = log2FoldChange, y = -log10(pvalue), col = sig_cat)) +
  geom_point(size = 2) +
  geom_text_repel(data = deseq2_results_dat %>%
                    filter(padj < 0.1),
                  aes(label = symbol), col = "black") +
  scale_color_manual("FDR < 0.1", values = c("dodgerblue4", "dodgerblue3", "dodgerblue1", 
                                             "tomato1", "tomato3", "tomato4",
                                             "gray")) +
  xlab("Log2 Fold Change") + ylab("-log10P") +
  theme_bw() +
  theme(axis.text.x = element_text(size = 12),
        axis.title.x = element_text(size = 12),
        axis.text.y = element_text(size = 12),
        axis.title.y = element_text(size = 12),
        legend.position = "bottom")
```

#### Differentially expressed gene counts

```
deg_count_dat <- deseq2_results_dat %>%
  filter(!is.na(sig_cat)) %>%
  filter(sig_cat != "FDR > 0.5") %>%
  group_by(sig_cat) %>%
  tally

ggplot(data = deg_count_dat,
       aes(x = sig_cat, y = n, fill = sig_cat)) +
  geom_bar(stat = "identity") +
  geom_text(data = deg_count_dat,
            aes(y = n + max(n)*0.05, label = n), size = 8) +
  scale_fill_manual("FDR < 0.1", values = c("dodgerblue4", "dodgerblue3", "dodgerblue1",
                                            "tomato1", "tomato3", "tomato4")) +
  guides(fill = "none") +
  xlab("") + ylab("count") +
  theme_bw() +
  theme(axis.text.x = element_text(size = 12, angle = 45, hjust = 1),
        axis.title.x = element_text(size = 12),
        axis.text.y = element_text(size = 12),
        axis.title.y = element_text(size = 12),
        legend.position = "bottom")
```

#### Differential gene expression tables

```
deseq2_sig_cat <- deseq2_results_dat %>%
  filter(padj < 0.5) %>%
  dplyr::select(sig_cat) %>%
  distinct %>%
  filter(!is.na(sig_cat)) %>%
  pull(sig_cat) %>%
  as.character

deseq2_results_table <- deseq2_results_dat %>%
      filter(!is.na(padj)) %>%
      dplyr::select(gene_id, symbol, baseMean, log2FoldChange, lfcSE, pvalue, padj, sig_cat) %>%
      filter(padj < 0.5) %>%
      arrange(padj) %>% 
      mutate(baseMean = round(baseMean, 3),
             log2FoldChange = round(log2FoldChange, 3),
             lfcSE = round(lfcSE, 3),
             pvalue = round(pvalue, 7),
             padj = round(padj, 3),
             sig_cat = as.character(sig_cat))

## Redefining colors because of weird bug in DT color handling
# Can't seem to handle colors with integer at end of word (e.g., "dodgerblue4")
dodgerblue4_new <- col2rgb("dodgerblue4")[,1]
dodgerblue4_new <- rgb(dodgerblue4_new[1], dodgerblue4_new[2], dodgerblue4_new[3], maxColorValue = 255)
dodgerblue3_new <- col2rgb("dodgerblue3")[,1]
dodgerblue3_new <- rgb(dodgerblue3_new[1], dodgerblue3_new[2], dodgerblue3_new[3], maxColorValue = 255)
dodgerblue1_new <- col2rgb("dodgerblue1")[,1]
dodgerblue1_new <- rgb(dodgerblue1_new[1], dodgerblue1_new[2], dodgerblue1_new[3], maxColorValue = 255)
tomato4_new <- col2rgb("tomato4")[,1]
tomato4_new <- rgb(tomato4_new[1], tomato4_new[2], tomato4_new[3], maxColorValue = 255)
tomato3_new <- col2rgb("tomato3")[,1]
tomato3_new <- rgb(tomato3_new[1], tomato3_new[2], tomato3_new[3], maxColorValue = 255)
tomato1_new <- col2rgb("tomato1")[,1]
tomato1_new <- rgb(tomato1_new[1], tomato1_new[2], tomato1_new[3], maxColorValue = 255)

htmltools::tagList(
      datatable(deseq2_results_table,
                caption = "Differential gene expression results in PBMC data from Walitt et al. 2024") %>%
      formatStyle("log2FoldChange", "sig_cat", 
                  backgroundColor = styleEqual(deseq2_sig_cat, c(dodgerblue4_new, tomato4_new, 
                                                                 dodgerblue3_new, tomato3_new,
                                                                 dodgerblue1_new, tomato1_new)),
                  color = "white"))
```

## Walitt *et al.* 2024 bulk RNA-seq in muscle tissue

### Introduction

These data were published as part of Walitt *et al.*
2024 in *Nature Communications*. This study deeply phenotyped
post-infectious ME/CFS cases, including both gene and protein expression
from multiple tissues, and compared to healthy controls.

| Study | Data Type | Tissue | Case/Control | Notes |
| --- | --- | --- | --- | --- |
| Walitt *et al.* 2024 | Gene expression (bulk RNA-seq) | Muscle | 12/13 | Females and males |

---

### Analysis

#### Overview

Walitt *et
al.* 2024 performed the differential expression analysis using
`limma`. They primarily report results from sex stratified
analyses, which we will avoid due to further reducing a small sample.
Here we use `DESeq2` instead, including sex as a covariate,
as well as BMI, following Walitt *et al.* 2024.

#### Load data

```
## Set to the appropriate directory in your environment
setwd("<Your Directory>")
```

```
## Important factors and covariates (e.g., case/control, sex)
sample_annot <- read.csv("data/walitt_sample_annot.csv", header = TRUE)

## Gene count data
gene_count_dat <- read.table("data/walitt_muscle_tx.tsv",
                         sep = "\t", header = TRUE) %>%
 dplyr::rename(symbol = Gene,
               gene_id = Molecule)
```

#### Prepare data for `DESeq2`

```
## Convert covariates to a matrix with ParticipantID as rownames
covar_mat <- sample_annot %>%
  dplyr::select(ParticipantID, Group, Birth.Sex, BMI) %>%
  dplyr::rename(case = Group,
                Sex = Birth.Sex) %>%
  mutate(ParticipantID = gsub(x = ParticipantID, pattern = "-", replacement = ".")) %>%
  column_to_rownames("ParticipantID") %>%
  mutate(case = factor(case),
         Sex = factor(Sex),
         BMI = scale(BMI))

## Convert gene counts to a matrix with ENSEMBL gene IDs as rownames and ParticipantID as colnames
gene_mat <- gene_count_dat %>%
  dplyr::select(-symbol) %>%
  column_to_rownames("gene_id")

## Preparing DESeq2 data structure for analysis
deseq2_count_dat <- DESeqDataSetFromMatrix(countData = gene_mat,
                                           colData = covar_mat[colnames(gene_mat),],
                                           design = ~ Sex + BMI + case)
```

#### Running differential expression analysis testing case/control status

```
deseq2_results <- DESeq(deseq2_count_dat)

deseq2_results_dat <- results(deseq2_results) %>%
  as.data.frame %>%
  arrange(padj) %>%
  rownames_to_column("gene_id") %>%
  ## Merge in gene symbol information
  left_join(gene_count_dat %>%
              dplyr::select(gene_id, symbol)) %>%
  mutate(sig_cat = case_when(padj < 0.1 & log2FoldChange > 0 ~ "Up in cases; FDR < 0.1",
                             padj < 0.3 & log2FoldChange > 0 ~ "Up in cases; 0.3 < FDR < 0.1",
                             padj < 0.5 & log2FoldChange > 0 ~ "Up in cases; 0.5 < FDR < 0.3",
                             padj < 0.1 & log2FoldChange < 0 ~ "Down in cases; FDR < 0.1",
                             padj < 0.3 & log2FoldChange < 0 ~ "Down in cases; 0.3 < FDR < 0.1",
                             padj < 0.5 & log2FoldChange < 0 ~ "Down in cases; 0.5 < FDR < 0.3",
                             padj > 0.5 ~ "FDR > 0.5")) %>%
  mutate(sig_cat = factor(sig_cat, levels = c("Down in cases; FDR < 0.1", 
                                              "Down in cases; 0.3 < FDR < 0.1", 
                                              "Down in cases; 0.5 < FDR < 0.3",
                                              "Up in cases; 0.5 < FDR < 0.3",
                                              "Up in cases; 0.3 < FDR < 0.1",
                                              "Up in cases; FDR < 0.1",
                                              "FDR > 0.5")))
```

---

### Results

#### P-value distribution

```
## Histogram of p-values
ggplot(data = deseq2_results_dat,
       aes(x = pvalue)) +
  geom_histogram(fill = "gray", col = "black") +
  theme_bw()
```

```
## Estimate proportion of p-values that follow the null distribution (Uniform)
get.pi0(pvalues = deseq2_results_dat$pvalue)
```

```
## [1] 0.7090957
```

There is decent support for differentially expressed genes because
the null proportion is below 100%.

#### Volcano plot

```
ggplot(data = deseq2_results_dat %>% filter(!is.na(padj)),
       aes(x = log2FoldChange, y = -log10(pvalue), col = sig_cat)) +
  geom_point(size = 2) +
  geom_text_repel(data = deseq2_results_dat %>%
                    filter(padj < 0.1),
                  aes(label = symbol), col = "black") +
  scale_color_manual("FDR < 0.1", values = c("dodgerblue4", "dodgerblue3", "dodgerblue1", 
                                             "tomato1", "tomato3", "tomato4",
                                             "gray")) +
  xlab("Log2 Fold Change") + ylab("-log10P") +
  theme_bw() +
  theme(axis.text.x = element_text(size = 12),
        axis.title.x = element_text(size = 12),
        axis.text.y = element_text(size = 12),
        axis.title.y = element_text(size = 12),
        legend.position = "bottom")
```

#### Differentially expressed gene counts

```
deg_count_dat <- deseq2_results_dat %>%
  filter(!is.na(sig_cat)) %>%
  filter(sig_cat != "FDR > 0.5") %>%
  group_by(sig_cat) %>%
  tally

ggplot(data = deg_count_dat,
       aes(x = sig_cat, y = n, fill = sig_cat)) +
  geom_bar(stat = "identity") +
  geom_text(data = deg_count_dat,
            aes(y = n + max(n)*0.05, label = n), size = 8) +
  scale_fill_manual("FDR < 0.1", values = c("dodgerblue4", "dodgerblue3", "dodgerblue1",
                                            "tomato1", "tomato3", "tomato4")) +
  guides(fill = "none") +
  xlab("") + ylab("count") +
  theme_bw() +
  theme(axis.text.x = element_text(size = 12, angle = 45, hjust = 1),
        axis.title.x = element_text(size = 12),
        axis.text.y = element_text(size = 12),
        axis.title.y = element_text(size = 12),
        legend.position = "bottom")
```

#### Differential gene expression tables

```
deseq2_sig_cat <- deseq2_results_dat %>%
  filter(padj < 0.5) %>%
  dplyr::select(sig_cat) %>%
  distinct %>%
  filter(!is.na(sig_cat)) %>%
  pull(sig_cat) %>%
  as.character

deseq2_results_table <- deseq2_results_dat %>%
      filter(!is.na(padj)) %>%
      dplyr::select(gene_id, symbol, baseMean, log2FoldChange, lfcSE, pvalue, padj, sig_cat) %>%
      filter(padj < 0.5) %>%
      arrange(padj) %>% 
      mutate(baseMean = round(baseMean, 3),
             log2FoldChange = round(log2FoldChange, 3),
             lfcSE = round(lfcSE, 3),
             pvalue = round(pvalue, 7),
             padj = round(padj, 3),
             sig_cat = as.character(sig_cat))

## Redefining colors because of weird bug in DT color handling
# Can't seem to handle colors with integer at end of word (e.g., "dodgerblue4")
dodgerblue4_new <- col2rgb("dodgerblue4")[,1]
dodgerblue4_new <- rgb(dodgerblue4_new[1], dodgerblue4_new[2], dodgerblue4_new[3], maxColorValue = 255)
dodgerblue3_new <- col2rgb("dodgerblue3")[,1]
dodgerblue3_new <- rgb(dodgerblue3_new[1], dodgerblue3_new[2], dodgerblue3_new[3], maxColorValue = 255)
dodgerblue1_new <- col2rgb("dodgerblue1")[,1]
dodgerblue1_new <- rgb(dodgerblue1_new[1], dodgerblue1_new[2], dodgerblue1_new[3], maxColorValue = 255)
tomato4_new <- col2rgb("tomato4")[,1]
tomato4_new <- rgb(tomato4_new[1], tomato4_new[2], tomato4_new[3], maxColorValue = 255)
tomato3_new <- col2rgb("tomato3")[,1]
tomato3_new <- rgb(tomato3_new[1], tomato3_new[2], tomato3_new[3], maxColorValue = 255)
tomato1_new <- col2rgb("tomato1")[,1]
tomato1_new <- rgb(tomato1_new[1], tomato1_new[2], tomato1_new[3], maxColorValue = 255)

htmltools::tagList(
      datatable(deseq2_results_table,
                caption = "Differential gene expression results in muscle data from Walitt et al. 2024") %>%
      formatStyle("log2FoldChange", "sig_cat", 
                  backgroundColor = styleEqual(deseq2_sig_cat, c(dodgerblue4_new, tomato4_new, 
                                                                 dodgerblue3_new, tomato3_new,
                                                                 dodgerblue1_new, tomato1_new)),
                  color = "white"))
```

## Raijmakers *et al.* 2019 bulk RNA-seq in monocytes

### Introduction

These data were published as part of Raijmakers *et
al.* 2019 in the *Journal of Translational Medicine*.
This study was focused on Q fever fatigue syndrome (QFS), which similar
to ME/CFS, is post-infectious fatiguing disorder. The authors collected
RNA from monocytes, using Percoll isolation from PBMC, for 10 QFS cases,
10 ME/CFS cases, 10 recovered Q fever seropositive controls, and 10
healthy controls, all sex and age matched.

| Study | Data Type | Tissue | Case/Control | Notes |
| --- | --- | --- | --- | --- |
| Raijmakers *et al.* 2019 | Gene expression (bulk RNA-seq) | Monoctyes from PBMC | 10/10 | Females and males |

---

### Analysis

#### Overview

Raijmakers
*et al.* 2019 performed the differential expression analysis
using `DESeq2` after filtering out genes with mean count <
1. They do not describe the covariates they included in the model. They
identified two mitochondrial genes, *MT-RNR1* and
*MT-RNR2*, as genes with significantly lower expression in ME/CFS
cases. Our analysis is similar, though we filter to ME/CFS cases and
health controls, impute sex based on the Y chromosome-encoded gene
*DDX3Y*, and then include sex as a covariate.

#### Load data

```
## Set to the appropriate directory in your environment
setwd("<Your Directory>")
```

```
## Gene annotations from biomart
gene_annot <- read.table("data/gene_annot_from_biomart_20241001.tsv", 
                         sep = "\t", header = TRUE)

## Gene count data
gene_count_dat <- read.table("data/raijmakers_monocyte_tx.tsv",
                         sep = "\t", header = TRUE) %>%
  left_join(gene_annot) %>%
  dplyr::rename(symbol = Gene,
                gene_id = Molecule)

## Build sample annotations and impute sex from DDX3Y expression
sample_annot <- data.frame(ParticipantID = names(gene_count_dat)[-1]) %>%
  mutate(case = ifelse(grepl(x = ParticipantID, pattern = "^CFS", perl = TRUE), 1, 0)) %>%
  left_join(gene_count_dat %>%
              filter(symbol == "DDX3Y") %>%
              dplyr::select(-c(gene_id, symbol)) %>%
              pivot_longer(everything(),
                           names_to = "ParticipantID",
                           values_to = "count") %>%
              mutate(Sex = ifelse(count > 100, "Male", "Female")) %>%
              dplyr::select(-count))
```

#### Prepare data for `DESeq2`

```
## Convert covariates to a matrix with ParticipantID as rownames
covar_mat <- sample_annot %>%
  mutate(Sex = ifelse(Sex == "Female", 1, 0)) %>%
  dplyr::select(ParticipantID, case, Sex) %>%
  column_to_rownames("ParticipantID") %>%
  mutate(case = factor(case),
         Sex = factor(Sex))

## Convert gene counts to a matrix with ENSEMBL gene IDs as rownames and ParticipantID as colnames
gene_mat <- gene_count_dat %>%
  dplyr::select(-symbol) %>%
  column_to_rownames("gene_id")

## Filter out lowly expressed genes (mean count < 1)
gene_mat <- gene_mat[apply(gene_mat, 1, function(x) mean(x)) >= 1,]

## Preparing DESeq2 data structure for analysis
deseq2_count_dat <- DESeqDataSetFromMatrix(countData = gene_mat,
                                           colData = covar_mat[colnames(gene_mat),],
                                           design = ~ Sex + case)
```

#### Running differential expression analysis testing case/control status

```
deseq2_results <- DESeq(deseq2_count_dat)

deseq2_results_dat <- results(deseq2_results) %>%
  as.data.frame %>%
  arrange(padj) %>%
  rownames_to_column("gene_id") %>%
  ## Merge in gene symbol information
  left_join(gene_count_dat %>%
              dplyr::select(gene_id, symbol)) %>%
  mutate(sig_cat = case_when(padj < 0.1 & log2FoldChange > 0 ~ "Up in cases; FDR < 0.1",
                             padj < 0.3 & log2FoldChange > 0 ~ "Up in cases; 0.3 < FDR < 0.1",
                             padj < 0.5 & log2FoldChange > 0 ~ "Up in cases; 0.5 < FDR < 0.3",
                             padj < 0.1 & log2FoldChange < 0 ~ "Down in cases; FDR < 0.1",
                             padj < 0.3 & log2FoldChange < 0 ~ "Down in cases; 0.3 < FDR < 0.1",
                             padj < 0.5 & log2FoldChange < 0 ~ "Down in cases; 0.5 < FDR < 0.3",
                             padj > 0.5 ~ "FDR > 0.5")) %>%
  mutate(sig_cat = factor(sig_cat, levels = c("Down in cases; FDR < 0.1", 
                                              "Down in cases; 0.3 < FDR < 0.1", 
                                              "Down in cases; 0.5 < FDR < 0.3",
                                              "Up in cases; 0.5 < FDR < 0.3",
                                              "Up in cases; 0.3 < FDR < 0.1",
                                              "Up in cases; FDR < 0.1",
                                              "FDR > 0.5")))
```

---

### Results

#### P-value distribution

```
## Histogram of p-values
ggplot(data = deseq2_results_dat,
       aes(x = pvalue)) +
  geom_histogram(fill = "gray", col = "black") +
  theme_bw()
```

```
## Estimate proportion of p-values that follow the null distribution (Uniform)
get.pi0(pvalues = deseq2_results_dat$pvalue)
```

```
## [1] 0.9568879
```

There is some support for differentially expressed genes because the
null proportion is below 100%, though it is very weak.

#### Volcano plot

```
ggplot(data = deseq2_results_dat %>% filter(!is.na(padj)),
       aes(x = log2FoldChange, y = -log10(pvalue), col = sig_cat)) +
  geom_point(size = 2) +
  geom_text_repel(data = deseq2_results_dat %>%
                    filter(padj < 0.1),
                  aes(label = symbol), col = "black") +
  scale_color_manual("FDR < 0.1", values = c("dodgerblue4", "dodgerblue3", "dodgerblue1", 
                                             "tomato1", "tomato3", "tomato4",
                                             "gray")) +
  xlab("Log2 Fold Change") + ylab("-log10P") +
  theme_bw() +
  theme(axis.text.x = element_text(size = 12),
        axis.title.x = element_text(size = 12),
        axis.text.y = element_text(size = 12),
        axis.title.y = element_text(size = 12),
        legend.position = "bottom")
```

This analysis reproduces the key finding of *MT-RNR1* and
*MT-RNR2* being more lowly expressed in ME/CFS cases.

#### Differentially expressed gene counts

```
deg_count_dat <- deseq2_results_dat %>%
  filter(!is.na(sig_cat)) %>%
  filter(sig_cat != "FDR > 0.5") %>%
  group_by(sig_cat) %>%
  tally

ggplot(data = deg_count_dat,
       aes(x = sig_cat, y = n, fill = sig_cat)) +
  geom_bar(stat = "identity") +
  geom_text(data = deg_count_dat,
            aes(y = n + max(n)*0.05, label = n), size = 8) +
  scale_fill_manual("FDR < 0.1", values = c("dodgerblue4", "dodgerblue3", "dodgerblue1",
                                            "tomato1", "tomato3", "tomato4")) +
  guides(fill = "none") +
  xlab("") + ylab("count") +
  theme_bw() +
  theme(axis.text.x = element_text(size = 12, angle = 45, hjust = 1),
        axis.title.x = element_text(size = 12),
        axis.text.y = element_text(size = 12),
        axis.title.y = element_text(size = 12),
        legend.position = "bottom")
```

#### Differential gene expression tables

```
deseq2_sig_cat <- deseq2_results_dat %>%
  filter(padj < 0.5) %>%
  dplyr::select(sig_cat) %>%
  distinct %>%
  filter(!is.na(sig_cat)) %>%
  pull(sig_cat) %>%
  as.character

deseq2_results_table <- deseq2_results_dat %>%
      filter(!is.na(padj)) %>%
      dplyr::select(gene_id, symbol, baseMean, log2FoldChange, lfcSE, pvalue, padj, sig_cat) %>%
      filter(padj < 0.5) %>%
      arrange(padj) %>% 
      mutate(baseMean = round(baseMean, 3),
             log2FoldChange = round(log2FoldChange, 3),
             lfcSE = round(lfcSE, 3),
             pvalue = round(pvalue, 7),
             padj = round(padj, 3),
             sig_cat = as.character(sig_cat))

## Redefining colors because of weird bug in DT color handling
# Can't seem to handle colors with integer at end of word (e.g., "dodgerblue4")
dodgerblue4_new <- col2rgb("dodgerblue4")[,1]
dodgerblue4_new <- rgb(dodgerblue4_new[1], dodgerblue4_new[2], dodgerblue4_new[3], maxColorValue = 255)
dodgerblue3_new <- col2rgb("dodgerblue3")[,1]
dodgerblue3_new <- rgb(dodgerblue3_new[1], dodgerblue3_new[2], dodgerblue3_new[3], maxColorValue = 255)
dodgerblue1_new <- col2rgb("dodgerblue1")[,1]
dodgerblue1_new <- rgb(dodgerblue1_new[1], dodgerblue1_new[2], dodgerblue1_new[3], maxColorValue = 255)
tomato4_new <- col2rgb("tomato4")[,1]
tomato4_new <- rgb(tomato4_new[1], tomato4_new[2], tomato4_new[3], maxColorValue = 255)
tomato3_new <- col2rgb("tomato3")[,1]
tomato3_new <- rgb(tomato3_new[1], tomato3_new[2], tomato3_new[3], maxColorValue = 255)
tomato1_new <- col2rgb("tomato1")[,1]
tomato1_new <- rgb(tomato1_new[1], tomato1_new[2], tomato1_new[3], maxColorValue = 255)

htmltools::tagList(
      datatable(deseq2_results_table,
                caption = "Differential gene expression results in muscle data from Walitt et al. 2024") %>%
      formatStyle("log2FoldChange", "sig_cat", 
                  backgroundColor = styleEqual(deseq2_sig_cat, c(dodgerblue4_new, tomato4_new, 
                                                                 dodgerblue3_new, tomato3_new,
                                                                 dodgerblue1_new, tomato1_new)),
                  color = "white"))
```

## Walitt *et al.* 2024 SomaScan proteomics in plasma

### Introduction

These data were published as part of Walitt *et al.*
2024 in *Nature Communications*. This study deeply phenotyped
post-infectious ME/CFS cases, including both gene and protein expression
from multiple tissues, and compared to healthy controls.

| Study | Data Type | Tissue | Case/Control | Notes |
| --- | --- | --- | --- | --- |
| Walitt *et al.* 2024 | Protein expression (SomaScan) | Plasma | 15/18 | Females and males |

---

### Analysis

#### Overview

Walitt *et
al.* 2024 did not perform a traditional differential expression
analysis for the protein data sets. They state they were used in a
partial least squares discrimination analysis (PLSDA), uing
`mixOmics`, though they only present results for metabolites
based on this. This approach is based on choosing variables that are
found to be strongly predictive of the case/control status. Here we
perform a simpler linear regression-based testing of case/control status
with ANOVA, including sex and BMI as a covariate.

### Load data

```
## Set to the appropriate directory in your environment
setwd("<Your Directory>")
```

```
## Important factors and covariates (e.g., case/control, sex)
sample_annot <- read.csv("data/walitt_sample_annot.csv", header = TRUE) %>%
  dplyr::select(ParticipantID, Group, Birth.Sex, BMI) %>%
  dplyr::rename(case = Group,
                Sex = Birth.Sex) %>%
  mutate(ParticipantID = gsub(x = ParticipantID, pattern = "-", replacement = "."))

## Gene count data
protein_dat <- read.table("data/walitt_plasma_px.tsv",
                          sep = "\t", header = TRUE) %>%
 dplyr::rename(symbol = Gene,
               gene_id = Molecule)
```

#### Prepare data for linear regression testing

```
## Grab protein identification information
plasma_annot <- protein_dat %>%
  dplyr::select(gene_id, Protein, symbol, UniProt) %>%
  dplyr::rename(seq_id = gene_id) 

## Process and merge together protein expression data and sample annotations
plasma_dat <- protein_dat %>%
  dplyr::rename(seq_id = gene_id) %>%
  dplyr::select(seq_id, contains("map")) %>%
  pivot_longer(-seq_id, names_to = "ParticipantID", values_to = "intensity") %>%
  pivot_wider(id_cols = ParticipantID, names_from = "seq_id", values_from = "intensity") %>%
  left_join(sample_annot)
```

#### Running differential expression analysis testing case/control status

```
## Grab all the SomaScan aptamer IDs
plasma_soma_seqid <- grep(x = colnames(plasma_dat), pattern = "SeqId", value = TRUE)

## Perform linear regression analysis
### H0: log10(protein) ~ intercept + Sex + BMI + case/control
### HA: log10(protein) ~ intercept + Sex + BMI
pval <- coef <- coef_se <- rep(NA, length(plasma_soma_seqid))
for (i in 1:length(plasma_soma_seqid)) {
  fit1 <- lm(paste0("log10(`", plasma_soma_seqid[i], "`) ~ 1 + Sex + BMI + case"), data = plasma_dat)
  fit0 <- lm(paste0("log10(`", plasma_soma_seqid[i], "`) ~ 1 + Sex + BMI"), data = plasma_dat)
  pval[i] <- anova(fit1, fit0)$`Pr(>F)`[2]
  coef[i] <- fit1$coefficients[2]
  coef_se[i] <- summary(fit1)$coefficients["case", "Std. Error"]
}

## Make results data.frame
results_dat <- data.frame(seq_id = plasma_soma_seqid,
                             pval = pval,
                             coef = coef,
                             coefSE = coef_se) %>%
  mutate(padj = p.adjust(pval, method = "BH")) %>%
  left_join(plasma_annot) %>%
  mutate(sig_cat = case_when(padj < 0.1 & coef > 0 ~ "Up in cases; FDR < 0.1",
                             padj < 0.3 & coef > 0 ~ "Up in cases; 0.3 < FDR < 0.1",
                             padj < 0.5 & coef > 0 ~ "Up in cases; 0.5 < FDR < 0.3",
                             padj < 0.1 & coef < 0 ~ "Down in cases; FDR < 0.1",
                             padj < 0.3 & coef < 0 ~ "Down in cases; 0.3 < FDR < 0.1",
                             padj < 0.5 & coef < 0 ~ "Down in cases; 0.5 < FDR < 0.3",
                             padj > 0.5 ~ "FDR > 0.5")) %>%
  mutate(sig_cat = factor(sig_cat, levels = c("Down in cases; FDR < 0.1", 
                                              "Down in cases; 0.3 < FDR < 0.1", 
                                              "Down in cases; 0.5 < FDR < 0.3",
                                              "Up in cases; 0.5 < FDR < 0.3",
                                              "Up in cases; 0.3 < FDR < 0.1",
                                              "Up in cases; FDR < 0.1",
                                              "FDR > 0.5")))
```

---

### Results

#### P-value distribution

```
## Histogram of p-values
ggplot(data = results_dat,
       aes(x = pval)) +
  geom_histogram(fill = "gray", col = "black") +
  theme_bw()
```

```
## Estimate proportion of p-values that follow the null distribution (Uniform)
get.pi0(pvalues = results_dat$pval)
```

```
## [1] 0.9946849
```

There is weak support for differentially expressed proteins because
the null proportion is just below 100%.

#### Volcano plot

```
ggplot(data = results_dat %>% filter(!is.na(padj)),
       aes(x = coef, y = -log10(pval), col = sig_cat)) +
  geom_point(size = 2) +
  geom_text_repel(data = results_dat %>%
                    filter(padj < 0.5),
                  aes(label = symbol), col = "black") +
  scale_color_manual("FDR < 0.1", values = c("dodgerblue4", "dodgerblue3", "dodgerblue1", 
                                             "tomato1", "tomato3", "tomato4",
                                             "gray"),
                     drop = FALSE) +
  xlab("Log10 Fold Change") + ylab("-log10P") +
  theme_bw() +
  theme(axis.text.x = element_text(size = 12),
        axis.title.x = element_text(size = 12),
        axis.text.y = element_text(size = 12),
        axis.title.y = element_text(size = 12),
        legend.position = "bottom")
```

#### Differentially expressed protein counts

```
dep_count_dat <- results_dat %>%
  filter(!is.na(sig_cat)) %>%
  filter(sig_cat != "FDR > 0.5") %>%
  group_by(sig_cat) %>%
  tally %>%
  complete()

ggplot(data = dep_count_dat,
       aes(x = sig_cat, y = n, fill = sig_cat)) +
  geom_bar(stat = "identity") +
  geom_text(data = dep_count_dat,
            aes(y = n + max(n)*0.05, label = n), size = 8) +
  scale_fill_manual("FDR < 0.1", values = c("dodgerblue1",
                                            "tomato1")) +
  guides(fill = "none") +
  xlab("") + ylab("count") +
  theme_bw() +
  theme(axis.text.x = element_text(size = 12, angle = 45, hjust = 1),
        axis.title.x = element_text(size = 12),
        axis.text.y = element_text(size = 12),
        axis.title.y = element_text(size = 12),
        legend.position = "bottom")
```

#### Differential protein expression tables

```
sig_cat <- results_dat %>%
  filter(padj < 0.5) %>%
  dplyr::select(sig_cat) %>%
  distinct %>%
  filter(!is.na(sig_cat)) %>%
  pull(sig_cat) %>%
  as.character

if (length(sig_cat) > 0) {
  results_table <- results_dat %>%
      filter(!is.na(padj)) %>%
      dplyr::select(seq_id, UniProt, Protein, symbol, coef, coefSE, pval, padj, sig_cat) %>%
      filter(padj < 0.5) %>%
      arrange(padj) %>% 
      mutate(coef = round(coef, 3),
             coefSE = round(coefSE, 3),
             pval = round(pval, 7),
             padj = round(padj, 3),
             sig_cat = as.character(sig_cat))

## Redefining colors because of weird bug in DT color handling
# Can't seem to handle colors with integer at end of word (e.g., "dodgerblue4")
dodgerblue4_new <- col2rgb("dodgerblue4")[,1]
dodgerblue4_new <- rgb(dodgerblue4_new[1], dodgerblue4_new[2], dodgerblue4_new[3], maxColorValue = 255)
dodgerblue3_new <- col2rgb("dodgerblue3")[,1]
dodgerblue3_new <- rgb(dodgerblue3_new[1], dodgerblue3_new[2], dodgerblue3_new[3], maxColorValue = 255)
dodgerblue1_new <- col2rgb("dodgerblue1")[,1]
dodgerblue1_new <- rgb(dodgerblue1_new[1], dodgerblue1_new[2], dodgerblue1_new[3], maxColorValue = 255)
tomato4_new <- col2rgb("tomato4")[,1]
tomato4_new <- rgb(tomato4_new[1], tomato4_new[2], tomato4_new[3], maxColorValue = 255)
tomato3_new <- col2rgb("tomato3")[,1]
tomato3_new <- rgb(tomato3_new[1], tomato3_new[2], tomato3_new[3], maxColorValue = 255)
tomato1_new <- col2rgb("tomato1")[,1]
tomato1_new <- rgb(tomato1_new[1], tomato1_new[2], tomato1_new[3], maxColorValue = 255)

htmltools::tagList(
      datatable(results_table,
                caption = "Differential protein expression results in plasma data from Walitt et al. 2024") %>%
      formatStyle("coef", "sig_cat", 
                  backgroundColor = styleEqual(c("Down in cases; FDR < 0.1", "Up in cases; FDR < 0.1",
                                                 "Down in cases; 0.3 < FDR < 0.1", "Up in cases; 0.3 < FDR < 0.1",
                                                 "Down in cases; 0.5 < FDR < 0.3", "Up in cases; 0.5 < FDR < 0.3"), 
                                               c(dodgerblue4_new, tomato4_new, 
                                                 dodgerblue3_new, tomato3_new,
                                                 dodgerblue1_new, tomato1_new)),
                  color = "white"))
}
```

## Walitt *et al.* 2024 SomaScan proteomics in CSF

### Introduction

These data were published as part of Walitt *et al.*
2024 in *Nature Communications*. This study deeply phenotyped
post-infectious ME/CFS cases, including both gene and protein expression
from multiple tissues, and compared to healthy controls.

| Study | Data Type | Tissue | Case/Control | Notes |
| --- | --- | --- | --- | --- |
| Walitt *et al.* 2024 | Protein expression (SomaScan) | CSF | 15/18 | Females and males |

---

### Analysis

#### Overview

Walitt *et
al.* 2024 did not perform a traditional differential expression
analysis for the protein data sets. They state they were used in a
partial least squares discrimination analysis (PLSDA), uing
`mixOmics`, though they only present results for metabolites
based on this. This approach is based on choosing variables that are
found to be strongly predictive of the case/control status. Here we
perform a simpler linear regression-based testing of case/control status
with ANOVA, including sex and BMI as a covariate.

#### Load data

```
## Set to the appropriate directory in your environment
setwd("<Your Directory>")
```

```
## Important factors and covariates (e.g., case/control, sex)
sample_annot <- read.csv("data/walitt_sample_annot.csv", header = TRUE) %>%
  dplyr::select(ParticipantID, Group, Birth.Sex, BMI) %>%
  dplyr::rename(case = Group,
                Sex = Birth.Sex) %>%
  mutate(ParticipantID = gsub(x = ParticipantID, pattern = "-", replacement = "."))

## Gene count data
protein_dat <- read.table("data/walitt_csf_px.tsv",
                          sep = "\t", header = TRUE) %>%
 dplyr::rename(symbol = Gene,
               gene_id = Molecule)
```

#### Prepare data for linear regression testing

```
## Grab protein identification information
csf_annot <- protein_dat %>%
  dplyr::select(gene_id, Protein, symbol, UniProt) %>%
  dplyr::rename(seq_id = gene_id) 

## Process and merge together protein expression data and sample annotations
csf_dat <- protein_dat %>%
  dplyr::rename(seq_id = gene_id) %>%
  dplyr::select(seq_id, contains("map")) %>%
  pivot_longer(-seq_id, names_to = "ParticipantID", values_to = "intensity") %>%
  pivot_wider(id_cols = ParticipantID, names_from = "seq_id", values_from = "intensity") %>%
  left_join(sample_annot)
```

#### Running differential expression analysis testing case/control status

```
## Grab all the SomaScan aptamer IDs
csf_soma_seqid <- grep(x = colnames(csf_dat), pattern = "SeqId", value = TRUE)

## Perform linear regression analysis
### H0: log10(protein) ~ intercept + Sex + BMI + case/control
### HA: log10(protein) ~ intercept + Sex + BMI
pval <- coef <- coef_se <- rep(NA, length(csf_soma_seqid))
for (i in 1:length(csf_soma_seqid)) {
  fit1 <- lm(paste0("log10(`", csf_soma_seqid[i], "`) ~ 1 + Sex + BMI + case"), data = csf_dat)
  fit0 <- lm(paste0("log10(`", csf_soma_seqid[i], "`) ~ 1 + Sex + BMI"), data = csf_dat)
  pval[i] <- anova(fit1, fit0)$`Pr(>F)`[2]
  coef[i] <- fit1$coefficients[2]
  coef_se[i] <- summary(fit1)$coefficients["case", "Std. Error"]
}

## Make results data.frame
results_dat <- data.frame(seq_id = csf_soma_seqid,
                          pval = pval,
                          coef = coef,
                          coefSE = coef_se) %>%
  mutate(padj = p.adjust(pval, method = "BH")) %>%
  left_join(csf_annot) %>%
  mutate(sig_cat = case_when(padj < 0.1 & coef > 0 ~ "Up in cases; FDR < 0.1",
                             padj < 0.3 & coef > 0 ~ "Up in cases; 0.3 < FDR < 0.1",
                             padj < 0.5 & coef > 0 ~ "Up in cases; 0.5 < FDR < 0.3",
                             padj < 0.1 & coef < 0 ~ "Down in cases; FDR < 0.1",
                             padj < 0.3 & coef < 0 ~ "Down in cases; 0.3 < FDR < 0.1",
                             padj < 0.5 & coef < 0 ~ "Down in cases; 0.5 < FDR < 0.3",
                             padj > 0.5 ~ "FDR > 0.5")) %>%
  mutate(sig_cat = factor(sig_cat, levels = c("Down in cases; FDR < 0.1", 
                                              "Down in cases; 0.3 < FDR < 0.1", 
                                              "Down in cases; 0.5 < FDR < 0.3",
                                              "Up in cases; 0.5 < FDR < 0.3",
                                              "Up in cases; 0.3 < FDR < 0.1",
                                              "Up in cases; FDR < 0.1",
                                              "FDR > 0.5")))
```

---

### Results

#### P-value distribution

```
## Histogram of p-values
ggplot(data = results_dat,
       aes(x = pval)) +
  geom_histogram(fill = "gray", col = "black") +
  theme_bw()
```

```
## Estimate proportion of p-values that follow the null distribution (Uniform)
get.pi0(pvalues = results_dat$pval)
```

```
## [1] 1
```

There is essentially no support for differentially expressed proteins
because the null proportion is at 100%. Furthermore, none of the
proteins have an adjusted p-value < 0.5.

#### Volcano plot

```
ggplot(data = results_dat %>% filter(!is.na(padj)),
       aes(x = coef, y = -log10(pval), col = sig_cat)) +
  geom_point(size = 2) +
  geom_text_repel(data = results_dat %>%
                    filter(padj < 0.5),
                  aes(label = symbol), col = "black") +
  scale_color_manual("FDR < 0.1", values = c("dodgerblue4", "dodgerblue3", "dodgerblue1", 
                                             "tomato1", "tomato3", "tomato4",
                                             "gray"),
                     drop = FALSE) +
  xlab("Log10 Fold Change") + ylab("-log10P") +
  theme_bw() +
  theme(axis.text.x = element_text(size = 12),
        axis.title.x = element_text(size = 12),
        axis.text.y = element_text(size = 12),
        axis.title.y = element_text(size = 12),
        legend.position = "bottom")
```

#### Differentially expressed protein counts

```
dep_count_dat <- results_dat %>%
  filter(!is.na(sig_cat)) %>%
  filter(sig_cat != "FDR > 0.5") %>%
  group_by(sig_cat) %>%
  tally

if (nrow(dep_count_dat) > 0) {
  ggplot(data = dep_count_dat,
       aes(x = sig_cat, y = n, fill = sig_cat)) +
  geom_bar(stat = "identity") +
  geom_text(data = dep_count_dat,
            aes(y = n + max(n)*0.05, label = n), size = 8) +
  scale_fill_manual("FDR < 0.1", values = c("dodgerblue4", "dodgerblue3", "dodgerblue1",
                                            "tomato1", "tomato3", "tomato4"),
                    drop = FALSE) +
  guides(fill = "none") +
  xlab("") + ylab("count") +
  theme_bw() +
  theme(axis.text.x = element_text(size = 12, angle = 45, hjust = 1),
        axis.title.x = element_text(size = 12),
        axis.text.y = element_text(size = 12),
        axis.title.y = element_text(size = 12),
        legend.position = "bottom")
}
```

#### Differential protein expression tables

No differentially expressed proteins to report in a table.

```
sig_cat <- results_dat %>%
  filter(padj < 0.5) %>%
  dplyr::select(sig_cat) %>%
  distinct %>%
  filter(!is.na(sig_cat)) %>%
  pull(sig_cat) %>%
  as.character

if (length(sig_cat) > 0) {
  results_table <- results_dat %>%
      filter(!is.na(padj)) %>%
      dplyr::select(seq_id, UniProt, Protein, symbol, coef, coefSE, pval, padj, sig_cat) %>%
      filter(padj < 0.5) %>%
      arrange(padj) %>% 
      mutate(coef = round(coef, 3),
             coefSE = round(coefSE, 3),
             pval = round(pval, 7),
             padj = round(padj, 3))

## Redefining colors because of weird bug in DT color handling
# Can't seem to handle colors with integer at end of word (e.g., "dodgerblue4")
dodgerblue4_new <- col2rgb("dodgerblue4")[,1]
dodgerblue4_new <- rgb(dodgerblue4_new[1], dodgerblue4_new[2], dodgerblue4_new[3], maxColorValue = 255)
dodgerblue3_new <- col2rgb("dodgerblue3")[,1]
dodgerblue3_new <- rgb(dodgerblue3_new[1], dodgerblue3_new[2], dodgerblue3_new[3], maxColorValue = 255)
dodgerblue1_new <- col2rgb("dodgerblue1")[,1]
dodgerblue1_new <- rgb(dodgerblue1_new[1], dodgerblue1_new[2], dodgerblue1_new[3], maxColorValue = 255)
tomato4_new <- col2rgb("tomato4")[,1]
tomato4_new <- rgb(tomato4_new[1], tomato4_new[2], tomato4_new[3], maxColorValue = 255)
tomato3_new <- col2rgb("tomato3")[,1]
tomato3_new <- rgb(tomato3_new[1], tomato3_new[2], tomato3_new[3], maxColorValue = 255)
tomato1_new <- col2rgb("tomato1")[,1]
tomato1_new <- rgb(tomato1_new[1], tomato1_new[2], tomato1_new[3], maxColorValue = 255)

htmltools::tagList(
      datatable(results_table,
                caption = "Differential protein expression results in CSF data from Walitt et al. 2024") %>%
      formatStyle("coef", "sig_cat", 
                  backgroundColor = styleEqual(c("Down in cases; FDR < 0.1", "Up in cases; FDR < 0.1",
                                                 "Down in cases; 0.3 < FDR < 0.1", "Up in cases; 0.3 < FDR < 0.1",
                                                 "Down in cases; 0.5 < FDR < 0.3", "Up in cases; 0.5 < FDR < 0.3"), 
                                               c(dodgerblue4_new, tomato4_new, 
                                                 dodgerblue3_new, tomato3_new,
                                                 dodgerblue1_new, tomato1_new)),
                  color = "white"))
}
```

## Germain *et al.* 2021 SomaScan proteomics in plasma

### Introduction

These data were published as part of Germain *et al.*
2021 in *proteomes*. This study interrogated the plasma
proteomes of 20 female ME/CFS cases and compared to healthy female
controls.

| Study | Data Type | Tissue | Case/Control | Notes |
| --- | --- | --- | --- | --- |
| Germain *et al.* 2021 | Protein expression (SomaScan) | Plasma | 20/20 | Females |

---

### Analysis

#### Overview

Germain *et
al.* 2021 tested for differentially expressed proteins using the
Wilcoxon rank-sum test, a non-parametric approach. This is a reasonable
approach given the relatively small sample size and lack of covariates.
Here we perform a linear regression-based testing of case/control status
with ANOVA to be consistent with our prior analyses of SomaScan data
from Walitt *et al.* 2024.

#### Load data

```
## Set to the appropriate directory in your environment
setwd("<Your Directory>")
```

```
## Important factors and covariates (e.g., case/control, sex)
sample_annot <- read.table("data/germain_sample_annot.tsv", header = TRUE, sep = "\t") %>%
  mutate(case = as.numeric(Phenotype == "ME/CFS")) %>%
  dplyr::select(ParticipantID, case)

## Gene count data
protein_dat <- fread("data/germain_plasma_px.tsv", data.table = FALSE) %>%
 dplyr::rename(symbol = Gene,
               seq_id = Molecule) %>%
 mutate(seq_id = paste("SeqId", seq_id, sep = "."))
```

#### Prepare data for linear regression testing

```
## Grab protein identification information
plasma_annot <- protein_dat %>%
  dplyr::select(seq_id, Protein, symbol, UniProt)

## Process and merge together protein expression data and sample annotations
plasma_dat <- protein_dat %>%
  dplyr::select(-c(Protein, symbol, UniProt)) %>%
  pivot_longer(-seq_id, names_to = "ParticipantID", values_to = "intensity") %>%
  pivot_wider(id_cols = ParticipantID, names_from = "seq_id", values_from = "intensity") %>%
  left_join(sample_annot)
```

#### Running differential expression analysis testing case/control status

```
## Grab all the SomaScan aptamer IDs
plasma_soma_seqid <- grep(x = colnames(plasma_dat), pattern = "SeqId", value = TRUE)

## Perform linear regression analysis
### H0: log10(protein) ~ intercept + Sex + case/control
### HA: log10(protein) ~ intercept + Sex
pval <- coef <- coef_se <- rep(NA, length(plasma_soma_seqid))
for (i in 1:length(plasma_soma_seqid)) {
  fit1 <- lm(paste0("log10(`", plasma_soma_seqid[i], "`) ~ 1 + case"), data = plasma_dat)
  fit0 <- lm(paste0("log10(`", plasma_soma_seqid[i], "`) ~ 1"), data = plasma_dat)
  pval[i] <- anova(fit1, fit0)$`Pr(>F)`[2]
  coef[i] <- fit1$coefficients[2]
  coef_se[i] <- summary(fit1)$coefficients["case", "Std. Error"]
}

## Make results data.frame
results_dat <- data.frame(seq_id = plasma_soma_seqid,
                             pval = pval,
                             coef = coef,
                             coefSE = coef_se) %>%
  mutate(padj = p.adjust(pval, method = "BH")) %>%
  left_join(plasma_annot) %>%
  mutate(sig_cat = case_when(padj < 0.1 & coef > 0 ~ "Up in cases; FDR < 0.1",
                             padj < 0.3 & coef > 0 ~ "Up in cases; 0.3 < FDR < 0.1",
                             padj < 0.5 & coef > 0 ~ "Up in cases; 0.5 < FDR < 0.3",
                             padj < 0.1 & coef < 0 ~ "Down in cases; FDR < 0.1",
                             padj < 0.3 & coef < 0 ~ "Down in cases; 0.3 < FDR < 0.1",
                             padj < 0.5 & coef < 0 ~ "Down in cases; 0.5 < FDR < 0.3",
                             padj > 0.5 ~ "FDR > 0.5")) %>%
  mutate(sig_cat = factor(sig_cat, levels = c("Down in cases; FDR < 0.1", 
                                              "Down in cases; 0.3 < FDR < 0.1", 
                                              "Down in cases; 0.5 < FDR < 0.3",
                                              "Up in cases; 0.5 < FDR < 0.3",
                                              "Up in cases; 0.3 < FDR < 0.1",
                                              "Up in cases; FDR < 0.1",
                                              "FDR > 0.5")))
```

---

### Results

#### P-value distribution

```
## Histogram of p-values
ggplot(data = results_dat,
       aes(x = pval)) +
  geom_histogram(fill = "gray", col = "black") +
  theme_bw()
```

```
## Estimate proportion of p-values that follow the null distribution (Uniform)
get.pi0(pvalues = results_dat$pval)
```

```
## [1] 0.7456472
```

There is decent support for differentially expressed proteins because
the null proportion is below 100%.

#### Volcano plot

```
ggplot(data = results_dat %>% filter(!is.na(padj)),
       aes(x = coef, y = -log10(pval), col = sig_cat)) +
  geom_point(size = 2) +
  geom_text_repel(data = results_dat %>%
                    filter(padj < 0.5),
                  aes(label = symbol), col = "black") +
  scale_color_manual("FDR < 0.1", values = c("dodgerblue4", "dodgerblue3", "dodgerblue1", 
                                             "tomato1", "tomato3", "tomato4",
                                             "gray"),
                     drop = FALSE) +
  xlab("Log10 Fold Change") + ylab("-log10P") +
  theme_bw() +
  theme(axis.text.x = element_text(size = 12),
        axis.title.x = element_text(size = 12),
        axis.text.y = element_text(size = 12),
        axis.title.y = element_text(size = 12),
        legend.position = "bottom")
```

#### Differentially expressed protein counts

```
dep_count_dat <- results_dat %>%
  filter(!is.na(sig_cat)) %>%
  filter(sig_cat != "FDR > 0.5") %>%
  group_by(sig_cat) %>%
  tally

ggplot(data = dep_count_dat,
       aes(x = sig_cat, y = n, fill = sig_cat)) +
  geom_bar(stat = "identity") +
  geom_text(data = dep_count_dat,
            aes(y = n + max(n)*0.05, label = n), size = 8) +
  scale_fill_manual("FDR < 0.1", values = c("dodgerblue3", "dodgerblue1",
                                            "tomato1", "tomato3", "tomato4"),
                    drop = TRUE) +
  guides(fill = "none") +
  xlab("") + ylab("count") +
  theme_bw() +
  theme(axis.text.x = element_text(size = 12, angle = 45, hjust = 1),
        axis.title.x = element_text(size = 12),
        axis.text.y = element_text(size = 12),
        axis.title.y = element_text(size = 12),
        legend.position = "bottom")
```

#### Differential protein expression tables

```
sig_cat <- results_dat %>%
  filter(padj < 0.5) %>%
  dplyr::select(sig_cat) %>%
  distinct %>%
  filter(!is.na(sig_cat)) %>%
  pull(sig_cat) %>%
  as.character

if (length(sig_cat) > 0) {
  results_table <- results_dat %>%
      filter(!is.na(padj)) %>%
      dplyr::select(seq_id, UniProt, Protein, symbol, coef, coefSE, pval, padj, sig_cat) %>%
      filter(padj < 0.5) %>%
      arrange(padj) %>% 
      mutate(coef = round(coef, 3),
             coefSE = round(coefSE, 3),
             pval = round(pval, 7),
             padj = round(padj, 3),
             sig_cat = as.character(sig_cat))

## Redefining colors because of weird bug in DT color handling
# Can't seem to handle colors with integer at end of word (e.g., "dodgerblue4")
dodgerblue4_new <- col2rgb("dodgerblue4")[,1]
dodgerblue4_new <- rgb(dodgerblue4_new[1], dodgerblue4_new[2], dodgerblue4_new[3], maxColorValue = 255)
dodgerblue3_new <- col2rgb("dodgerblue3")[,1]
dodgerblue3_new <- rgb(dodgerblue3_new[1], dodgerblue3_new[2], dodgerblue3_new[3], maxColorValue = 255)
dodgerblue1_new <- col2rgb("dodgerblue1")[,1]
dodgerblue1_new <- rgb(dodgerblue1_new[1], dodgerblue1_new[2], dodgerblue1_new[3], maxColorValue = 255)
tomato4_new <- col2rgb("tomato4")[,1]
tomato4_new <- rgb(tomato4_new[1], tomato4_new[2], tomato4_new[3], maxColorValue = 255)
tomato3_new <- col2rgb("tomato3")[,1]
tomato3_new <- rgb(tomato3_new[1], tomato3_new[2], tomato3_new[3], maxColorValue = 255)
tomato1_new <- col2rgb("tomato1")[,1]
tomato1_new <- rgb(tomato1_new[1], tomato1_new[2], tomato1_new[3], maxColorValue = 255)

htmltools::tagList(
      datatable(results_table,
                caption = "Differential protein expression results in plasma data from Germain et al. 2021") %>%
      formatStyle("coef", "sig_cat", 
                  backgroundColor = styleEqual(c("Down in cases; FDR < 0.1", "Up in cases; FDR < 0.1",
                                                 "Down in cases; 0.3 < FDR < 0.1", "Up in cases; 0.3 < FDR < 0.1",
                                                 "Down in cases; 0.5 < FDR < 0.3", "Up in cases; 0.5 < FDR < 0.3"), 
                                               c(dodgerblue4_new, tomato4_new, 
                                                 dodgerblue3_new, tomato3_new,
                                                 dodgerblue1_new, tomato1_new)),
                  color = "white"))
}
```

## Giloteaux *et al.* 2024 TMT mass spec proteomics in EVs

### Introduction

These data were published in Giloteaux *et al.*
2024 in *Journal of Extracellular Vesicles*. This study
interrogated the proteomes extracellular vessicles isolated from plasma
from female ME/CFS cases and compared to healthy female controls before
and after exercise. Subjects underwent an exercise challenge
(cardiopulmonary exercise test [CPET]) and plasma samples collected at
three different timepoints: prior CPET (T0), 15 minutes post CPET (T1),
and the day following CPET (T2). We will focus on T0 results when
comparing to other studies.

| Study | Data Type | Tissue | Case/Control | Notes |
| --- | --- | --- | --- | --- |
| Giloteaux *et al.* 2024 | Protein expression (mass-spec) | extracellular vessicles | 18/17 | Females |

---

### Analysis

#### Overview

Giloteaux *et
al.* 2024 has a complex tandem mass tag (TMT) experimental
design with 12 TMT 10-plex batches/experiments. Each batch represents
three samples from each of three participants (nine samples total) and
one pooled sample from all samples. Furthermore, each batch is comprised
of only cases or controls (thus six case and six control batches). This
does represent a challenging degree of partial confounding of
case/control status with batch, though the pooled sample somewhat
mitigates this problem.

To accommodate this design, Giloteaux *et al.* 2024 tested for
differentially expressed proteins using a complex bootstrap with
replacement procedure. Briefly, they performed 10,000 boostraps, each
time selecting three values from each TMT batch with replacement,
calculating a fold change (resulting in 18 fold changes), and then
summarizing with a median. 95% confidence intervals were constructed for
each gene based on these 10,000 intervals, and significance declared if
an interval covered 1. Here we perform a more conventional linear
regression-based testing of case/control status with ANOVA to be
consistent with our prior analyses of SomaScan-based data, though we do
note the challenge of any analysis given the batch structure and
relatively small sample size.

Giloteaux *et al.* 2024 also filtered out proteins that were
missing in more than 1/3 of samples (had to be observed in 8 out of 12
TMT batches). They then imputed missing values using a random forest
approach. We will instead treat missing samples as missing, but note
that these results will differ from the published results.

#### Load data

```
## Set to the appropriate directory in your environment
setwd("<Your Directory>")
```

```
## Important factors and covariates (e.g., case/control, sex)
sample_annot <- read.table("data/giloteaux_sample_annot.tsv", header = TRUE, sep = "\t") %>%
  mutate(case = as.numeric(Phenotype == "ME/CFS")) %>%
  dplyr::select(ParticipantID, case, timepoint)

## Split into timepoint-specific annotations
sample_annot_T0 <- sample_annot %>%
  filter(timepoint == "0h") %>%
  dplyr::select(-timepoint)
sample_annot_T1 <- sample_annot %>%
  filter(timepoint == "15min") %>%
  dplyr::select(-timepoint)
sample_annot_T2 <- sample_annot %>%
  filter(timepoint == "24h") %>%
  dplyr::select(-timepoint)

## Gene count data
protein_dat <- fread("data/giloteaux_ev_px.tsv", data.table = FALSE) %>%
 dplyr::rename(symbol = Molecule)
```

#### Prepare data for linear regression testing

```
## Grab protein identification information
ev_annot <- protein_dat %>%
  dplyr::select(symbol, UniProt, Entrez)

## Process and merge together protein expression data and sample annotations
ev_dat <- protein_dat %>%
  dplyr::select(-c(UniProt, Entrez)) %>%
  pivot_longer(-symbol, names_to = "ParticipantID", values_to = "intensity") %>%
  pivot_wider(id_cols = ParticipantID, names_from = "symbol", values_from = "intensity") %>%
  left_join(sample_annot)
```

#### Running differential expression analysis testing case/control status

```
## Grab all the SomaScan aptamer IDs
ev_proteins <- names(ev_dat)[!grepl(x = names(ev_dat), pattern = "ParticipantID|case|timepoint")]

## Stratify by timepoint
ev_dat_T0 <- ev_dat %>%
  filter(grepl(x = ParticipantID, pattern = "0h"))
ev_dat_T1 <- ev_dat %>%
  filter(grepl(x = ParticipantID, pattern = "15min"))
ev_dat_T2 <- ev_dat %>%
  filter(grepl(x = ParticipantID, pattern = "24h"))

## Perform linear regression analysis
### H0: log10(protein) ~ intercept + Sex + case/control
### HA: log10(protein) ~ intercept + Sex
pval <- coef <- coef_se <- rep(NA, length(ev_proteins))
for (i in 1:length(ev_proteins)) {
  fit1 <- lm(paste0("log10(`", ev_proteins[i], "` + 1) ~ 1 + case"), data = ev_dat_T0)
  fit0 <- lm(paste0("log10(`", ev_proteins[i], "` + 1) ~ 1"), data = ev_dat_T0)
  pval[i] <- anova(fit1, fit0)$`Pr(>F)`[2]
  coef[i] <- fit1$coefficients[2]
  coef_se[i] <- summary(fit1)$coefficients["case", "Std. Error"]
}

## Make results data.frame
results_T0_dat <- data.frame(symbol = ev_proteins,
                             pval = pval,
                             coef = coef,
                             coefSE = coef_se) %>%
  mutate(padj = p.adjust(pval, method = "BH")) %>%
  left_join(ev_annot) %>%
  mutate(sig_cat = case_when(padj < 0.1 & coef > 0 ~ "Up in cases; FDR < 0.1",
                             padj < 0.3 & coef > 0 ~ "Up in cases; 0.3 < FDR < 0.1",
                             padj < 0.5 & coef > 0 ~ "Up in cases; 0.5 < FDR < 0.3",
                             padj < 0.1 & coef < 0 ~ "Down in cases; FDR < 0.1",
                             padj < 0.3 & coef < 0 ~ "Down in cases; 0.3 < FDR < 0.1",
                             padj < 0.5 & coef < 0 ~ "Down in cases; 0.5 < FDR < 0.3",
                             padj > 0.5 ~ "FDR > 0.5")) %>%
  mutate(sig_cat = factor(sig_cat, levels = c("Down in cases; FDR < 0.1", 
                                              "Down in cases; 0.3 < FDR < 0.1", 
                                              "Down in cases; 0.5 < FDR < 0.3",
                                              "Up in cases; 0.5 < FDR < 0.3",
                                              "Up in cases; 0.3 < FDR < 0.1",
                                              "Up in cases; FDR < 0.1",
                                              "FDR > 0.5")))

## Perform linear regression analysis
### H0: log10(protein) ~ intercept + Sex + case/control
### HA: log10(protein) ~ intercept + Sex
pval <- coef <- coef_se <- rep(NA, length(ev_proteins))
for (i in 1:length(ev_proteins)) {
  fit1 <- lm(paste0("log10(`", ev_proteins[i], "` + 1) ~ 1 + case"), data = ev_dat_T1)
  fit0 <- lm(paste0("log10(`", ev_proteins[i], "` + 1) ~ 1"), data = ev_dat_T1)
  pval[i] <- anova(fit1, fit0)$`Pr(>F)`[2]
  coef[i] <- fit1$coefficients[2]
  coef_se[i] <- summary(fit1)$coefficients["case", "Std. Error"]
}

## Make results data.frame
results_T1_dat <- data.frame(symbol = ev_proteins,
                                pval = pval,
                                coef = coef,
                                coefSE = coef_se) %>%
  mutate(padj = p.adjust(pval, method = "BH")) %>%
  left_join(ev_annot) %>%
  mutate(sig_cat = case_when(padj < 0.1 & coef > 0 ~ "Up in cases; FDR < 0.1",
                             padj < 0.3 & coef > 0 ~ "Up in cases; 0.3 < FDR < 0.1",
                             padj < 0.5 & coef > 0 ~ "Up in cases; 0.5 < FDR < 0.3",
                             padj < 0.1 & coef < 0 ~ "Down in cases; FDR < 0.1",
                             padj < 0.3 & coef < 0 ~ "Down in cases; 0.3 < FDR < 0.1",
                             padj < 0.5 & coef < 0 ~ "Down in cases; 0.5 < FDR < 0.3",
                             padj > 0.5 ~ "FDR > 0.5")) %>%
  mutate(sig_cat = factor(sig_cat, levels = c("Down in cases; FDR < 0.1", 
                                              "Down in cases; 0.3 < FDR < 0.1", 
                                              "Down in cases; 0.5 < FDR < 0.3",
                                              "Up in cases; 0.5 < FDR < 0.3",
                                              "Up in cases; 0.3 < FDR < 0.1",
                                              "Up in cases; FDR < 0.1",
                                              "FDR > 0.5")))

## Perform linear regression analysis
### H0: log10(protein) ~ intercept + Sex + case/control
### HA: log10(protein) ~ intercept + Sex
pval <- coef <- coef_se <- rep(NA, length(ev_proteins))
for (i in 1:length(ev_proteins)) {
  fit1 <- lm(paste0("log10(`", ev_proteins[i], "` + 1) ~ 1 + case"), data = ev_dat_T2)
  fit0 <- lm(paste0("log10(`", ev_proteins[i], "` + 1) ~ 1"), data = ev_dat_T2)
  pval[i] <- anova(fit1, fit0)$`Pr(>F)`[2]
  coef[i] <- fit1$coefficients[2]
  coef_se[i] <- summary(fit1)$coefficients["case", "Std. Error"]
}

## Make results data.frame
results_T2_dat <- data.frame(symbol = ev_proteins,
                              pval = pval,
                              coef = coef,
                              coefSE = coef_se) %>%
  mutate(padj = p.adjust(pval, method = "BH")) %>%
  left_join(ev_annot) %>%
  mutate(sig_cat = case_when(padj < 0.1 & coef > 0 ~ "Up in cases; FDR < 0.1",
                             padj < 0.3 & coef > 0 ~ "Up in cases; 0.3 < FDR < 0.1",
                             padj < 0.5 & coef > 0 ~ "Up in cases; 0.5 < FDR < 0.3",
                             padj < 0.1 & coef < 0 ~ "Down in cases; FDR < 0.1",
                             padj < 0.3 & coef < 0 ~ "Down in cases; 0.3 < FDR < 0.1",
                             padj < 0.5 & coef < 0 ~ "Down in cases; 0.5 < FDR < 0.3",
                             padj > 0.5 ~ "FDR > 0.5")) %>%
  mutate(sig_cat = factor(sig_cat, levels = c("Down in cases; FDR < 0.1", 
                                              "Down in cases; 0.3 < FDR < 0.1", 
                                              "Down in cases; 0.5 < FDR < 0.3",
                                              "Up in cases; 0.5 < FDR < 0.3",
                                              "Up in cases; 0.3 < FDR < 0.1",
                                              "Up in cases; FDR < 0.1",
                                              "FDR > 0.5")))
```

---

### Results

#### T0

##### P-value distribution

```
## Histogram of p-values
ggplot(data = results_T0_dat,
       aes(x = pval)) +
  geom_histogram(fill = "gray", col = "black") +
  theme_bw()
```

```
## Estimate proportion of p-values that follow the null distribution (Uniform)
get.pi0(pvalues = results_T0_dat$pval)
```

```
## [1] 1
```

The p-value distribution is not ideal for T0. Nevertheless, we will
look at the differentially expressed proteins and look for consistency
and differences across timepoints.

##### Volcano plot

```
ggplot(data = results_T0_dat %>% filter(!is.na(padj)),
       aes(x = coef, y = -log10(pval), col = sig_cat)) +
  geom_point(size = 2) +
  geom_text_repel(data = results_T0_dat %>%
                    filter(padj < 0.5),
                  aes(label = symbol), col = "black") +
  scale_color_manual("FDR < 0.1", values = c("dodgerblue4", "dodgerblue3", "dodgerblue1", 
                                             "tomato1", "tomato3", "tomato4",
                                             "gray"),
                     drop = FALSE) +
  xlab("Log10 Fold Change") + ylab("-log10P") +
  theme_bw() +
  theme(axis.text.x = element_text(size = 12),
        axis.title.x = element_text(size = 12),
        axis.text.y = element_text(size = 12),
        axis.title.y = element_text(size = 12),
        legend.position = "bottom")
```

##### Differentially expressed protein counts

```
dep_count_T0_dat <- results_T0_dat %>%
  filter(!is.na(sig_cat)) %>%
  filter(sig_cat != "FDR > 0.5") %>%
  group_by(sig_cat) %>%
  tally

ggplot(data = dep_count_T0_dat,
       aes(x = sig_cat, y = n, fill = sig_cat)) +
  geom_bar(stat = "identity") +
  geom_text(data = dep_count_T0_dat,
            aes(y = n + max(n)*0.05, label = n), size = 8) +
  scale_fill_manual("FDR < 0.1", values = c("dodgerblue3", "dodgerblue1",
                                            "tomato1", "tomato3", "tomato4"),
                    drop = TRUE) +
  guides(fill = "none") +
  xlab("") + ylab("count") +
  theme_bw() +
  theme(axis.text.x = element_text(size = 12, angle = 45, hjust = 1),
        axis.title.x = element_text(size = 12),
        axis.text.y = element_text(size = 12),
        axis.title.y = element_text(size = 12),
        legend.position = "bottom")
```

##### Differential protein expression tables

```
sig_cat <- results_T0_dat %>%
  filter(padj < 0.5) %>%
  dplyr::select(sig_cat) %>%
  distinct %>%
  filter(!is.na(sig_cat)) %>%
  pull(sig_cat) %>%
  as.character

if (length(sig_cat) > 0) {
  results_T0_table <- results_T0_dat %>%
      filter(!is.na(padj)) %>%
      dplyr::select(symbol, UniProt, Entrez, coef, coefSE, pval, padj, sig_cat) %>%
      filter(padj < 0.5) %>%
      arrange(padj) %>% 
      mutate(coef = round(coef, 3),
             coefSE = round(coefSE, 3),
             pval = round(pval, 7),
             padj = round(padj, 3),
             sig_cat = as.character(sig_cat))

## Redefining colors because of weird bug in DT color handling
# Can't seem to handle colors with integer at end of word (e.g., "dodgerblue4")
dodgerblue4_new <- col2rgb("dodgerblue4")[,1]
dodgerblue4_new <- rgb(dodgerblue4_new[1], dodgerblue4_new[2], dodgerblue4_new[3], maxColorValue = 255)
dodgerblue3_new <- col2rgb("dodgerblue3")[,1]
dodgerblue3_new <- rgb(dodgerblue3_new[1], dodgerblue3_new[2], dodgerblue3_new[3], maxColorValue = 255)
dodgerblue1_new <- col2rgb("dodgerblue1")[,1]
dodgerblue1_new <- rgb(dodgerblue1_new[1], dodgerblue1_new[2], dodgerblue1_new[3], maxColorValue = 255)
tomato4_new <- col2rgb("tomato4")[,1]
tomato4_new <- rgb(tomato4_new[1], tomato4_new[2], tomato4_new[3], maxColorValue = 255)
tomato3_new <- col2rgb("tomato3")[,1]
tomato3_new <- rgb(tomato3_new[1], tomato3_new[2], tomato3_new[3], maxColorValue = 255)
tomato1_new <- col2rgb("tomato1")[,1]
tomato1_new <- rgb(tomato1_new[1], tomato1_new[2], tomato1_new[3], maxColorValue = 255)

htmltools::tagList(
      datatable(results_T0_table,
                caption = "Differential protein expression results in EV data at T0 from Giloteaux et al. 2024") %>%
      formatStyle("coef", "sig_cat", 
                  backgroundColor = styleEqual(c("Down in cases; FDR < 0.1", "Up in cases; FDR < 0.1",
                                                 "Down in cases; 0.3 < FDR < 0.1", "Up in cases; 0.3 < FDR < 0.1",
                                                 "Down in cases; 0.5 < FDR < 0.3", "Up in cases; 0.5 < FDR < 0.3"), 
                                               c(dodgerblue4_new, tomato4_new, 
                                                 dodgerblue3_new, tomato3_new,
                                                 dodgerblue1_new, tomato1_new)),
                  color = "white"))
}
```

#### T1

##### P-value distribution

```
## Histogram of p-values
ggplot(data = results_T1_dat,
       aes(x = pval)) +
  geom_histogram(fill = "gray", col = "black") +
  theme_bw()
```

```
## Estimate proportion of p-values that follow the null distribution (Uniform)
get.pi0(pvalues = results_T0_dat$pval)
```

```
## [1] 1
```

The p-value distribution suggests no significant differentially
expressed proteins at T1. Furthermore, no proteins had an FDR-adjusted
p-value < 0.1.

##### Volcano plot

```
ggplot(data = results_T1_dat %>% filter(!is.na(padj)),
       aes(x = coef, y = -log10(pval), col = sig_cat)) +
  geom_point(size = 2) +
  geom_text_repel(data = results_T1_dat %>%
                    filter(padj < 0.5),
                  aes(label = symbol), col = "black") +
  scale_color_manual("FDR < 0.1", values = c("dodgerblue4", "dodgerblue3", "dodgerblue1", 
                                             "tomato1", "tomato3", "tomato4",
                                             "gray"),
                     drop = FALSE) +
  xlab("Log10 Fold Change") + ylab("-log10P") +
  theme_bw() +
  theme(axis.text.x = element_text(size = 12),
        axis.title.x = element_text(size = 12),
        axis.text.y = element_text(size = 12),
        axis.title.y = element_text(size = 12),
        legend.position = "bottom")
```

##### Differentially expressed protein counts

```
dep_count_T1_dat <- results_T1_dat %>%
  filter(!is.na(sig_cat)) %>%
  filter(sig_cat != "FDR > 0.5") %>%
  group_by(sig_cat) %>%
  tally

if (nrow(dep_count_T1_dat) > 0) {
  ggplot(data = dep_count_T1_dat,
       aes(x = sig_cat, y = n, fill = sig_cat)) +
  geom_bar(stat = "identity") +
  geom_text(data = dep_count_T1_dat,
            aes(y = n + max(n)*0.05, label = n), size = 8) +
  scale_fill_manual("FDR < 0.1", values = c("dodgerblue3", "dodgerblue1",
                                            "tomato1", "tomato3", "tomato4"),
                    drop = TRUE) +
  guides(fill = "none") +
  xlab("") + ylab("count") +
  theme_bw() +
  theme(axis.text.x = element_text(size = 12, angle = 45, hjust = 1),
        axis.title.x = element_text(size = 12),
        axis.text.y = element_text(size = 12),
        axis.title.y = element_text(size = 12),
        legend.position = "bottom")
}
```

No differentially expressed proteins to report at T1.

##### Differential protein expression tables

```
sig_cat <- results_T1_dat %>%
  filter(padj < 0.5) %>%
  dplyr::select(sig_cat) %>%
  distinct %>%
  filter(!is.na(sig_cat)) %>%
  pull(sig_cat) %>%
  as.character

if (length(sig_cat) > 0) {
  results_T1_table <- results_T1_dat %>%
      filter(!is.na(padj)) %>%
      dplyr::select(symbol, UniProt, Entrez, coef, coefSE, pval, padj, sig_cat) %>%
      filter(padj < 0.5) %>%
      arrange(padj) %>% 
      mutate(coef = round(coef, 3),
             coefSE = round(coefSE, 3),
             pval = round(pval, 7),
             padj = round(padj, 3),
             sig_cat = as.character(sig_cat))

## Redefining colors because of weird bug in DT color handling
# Can't seem to handle colors with integer at end of word (e.g., "dodgerblue4")
dodgerblue4_new <- col2rgb("dodgerblue4")[,1]
dodgerblue4_new <- rgb(dodgerblue4_new[1], dodgerblue4_new[2], dodgerblue4_new[3], maxColorValue = 255)
dodgerblue3_new <- col2rgb("dodgerblue3")[,1]
dodgerblue3_new <- rgb(dodgerblue3_new[1], dodgerblue3_new[2], dodgerblue3_new[3], maxColorValue = 255)
dodgerblue1_new <- col2rgb("dodgerblue1")[,1]
dodgerblue1_new <- rgb(dodgerblue1_new[1], dodgerblue1_new[2], dodgerblue1_new[3], maxColorValue = 255)
tomato4_new <- col2rgb("tomato4")[,1]
tomato4_new <- rgb(tomato4_new[1], tomato4_new[2], tomato4_new[3], maxColorValue = 255)
tomato3_new <- col2rgb("tomato3")[,1]
tomato3_new <- rgb(tomato3_new[1], tomato3_new[2], tomato3_new[3], maxColorValue = 255)
tomato1_new <- col2rgb("tomato1")[,1]
tomato1_new <- rgb(tomato1_new[1], tomato1_new[2], tomato1_new[3], maxColorValue = 255)

htmltools::tagList(
      datatable(results_T1_table,
                caption = "Differential protein expression results in EV data at T1 from Giloteaux et al. 2024") %>%
      formatStyle("coef", "sig_cat", 
                  backgroundColor = styleEqual(c("Down in cases; FDR < 0.1", "Up in cases; FDR < 0.1",
                                                 "Down in cases; 0.3 < FDR < 0.1", "Up in cases; 0.3 < FDR < 0.1",
                                                 "Down in cases; 0.5 < FDR < 0.3", "Up in cases; 0.5 < FDR < 0.3"), 
                                               c(dodgerblue4_new, tomato4_new, 
                                                 dodgerblue3_new, tomato3_new,
                                                 dodgerblue1_new, tomato1_new)),
                  color = "white"))
}
```

#### T2

##### P-value distribution

```
## Histogram of p-values
ggplot(data = results_T2_dat,
       aes(x = pval)) +
  geom_histogram(fill = "gray", col = "black") +
  theme_bw()
```

```
## Estimate proportion of p-values that follow the null distribution (Uniform)
get.pi0(pvalues = results_T2_dat$pval)
```

```
## [1] 1
```

The p-value distribution suggests no significant differentially
expressed proteins at T2. Furthermore, no proteins had an FDR-adjusted
p-value < 0.1.

##### Volcano plot

```
ggplot(data = results_T2_dat %>% filter(!is.na(padj)),
       aes(x = coef, y = -log10(pval), col = sig_cat)) +
  geom_point(size = 2) +
  geom_text_repel(data = results_T2_dat %>%
                    filter(padj < 0.5),
                  aes(label = symbol), col = "black") +
  scale_color_manual("FDR < 0.1", values = c("dodgerblue4", "dodgerblue3", "dodgerblue1", 
                                             "tomato1", "tomato3", "tomato4",
                                             "gray"),
                     drop = FALSE) +
  xlab("Log10 Fold Change") + ylab("-log10P") +
  theme_bw() +
  theme(axis.text.x = element_text(size = 12),
        axis.title.x = element_text(size = 12),
        axis.text.y = element_text(size = 12),
        axis.title.y = element_text(size = 12),
        legend.position = "bottom")
```

##### Differentially expressed protein counts

```
dep_count_T2_dat <- results_T2_dat %>%
  filter(!is.na(sig_cat)) %>%
  filter(sig_cat != "FDR > 0.5") %>%
  group_by(sig_cat) %>%
  tally

if(nrow(dep_count_T2_dat) > 0) {
  ggplot(data = dep_count_T2_dat,
       aes(x = sig_cat, y = n, fill = sig_cat)) +
  geom_bar(stat = "identity") +
  geom_text(data = dep_count_T2_dat,
            aes(y = n + max(n)*0.05, label = n), size = 8) +
  scale_fill_manual("FDR < 0.1", values = c("dodgerblue3", "dodgerblue1",
                                            "tomato1", "tomato3", "tomato4"),
                    drop = TRUE) +
  guides(fill = "none") +
  xlab("") + ylab("count") +
  theme_bw() +
  theme(axis.text.x = element_text(size = 12, angle = 45, hjust = 1),
        axis.title.x = element_text(size = 12),
        axis.text.y = element_text(size = 12),
        axis.title.y = element_text(size = 12),
        legend.position = "bottom")
}
```

No differentially expressed proteins to report at T2.

##### Differential protein expression tables

```
sig_cat <- results_T2_dat %>%
  filter(padj < 0.5) %>%
  dplyr::select(sig_cat) %>%
  distinct %>%
  filter(!is.na(sig_cat)) %>%
  pull(sig_cat) %>%
  as.character

if (length(sig_cat) > 0) {
  results_T2_table <- results_T2_dat %>%
      filter(!is.na(padj)) %>%
      dplyr::select(symbol, UniProt, Entrez, coef, coefSE, pval, padj, sig_cat) %>%
      filter(padj < 0.5) %>%
      arrange(padj) %>% 
      mutate(coef = round(coef, 3),
             coefSE = round(coefSE, 3),
             pval = round(pval, 7),
             padj = round(padj, 3),
             sig_cat = as.character(sig_cat))

## Redefining colors because of weird bug in DT color handling
# Can't seem to handle colors with integer at end of word (e.g., "dodgerblue4")
dodgerblue4_new <- col2rgb("dodgerblue4")[,1]
dodgerblue4_new <- rgb(dodgerblue4_new[1], dodgerblue4_new[2], dodgerblue4_new[3], maxColorValue = 255)
dodgerblue3_new <- col2rgb("dodgerblue3")[,1]
dodgerblue3_new <- rgb(dodgerblue3_new[1], dodgerblue3_new[2], dodgerblue3_new[3], maxColorValue = 255)
dodgerblue1_new <- col2rgb("dodgerblue1")[,1]
dodgerblue1_new <- rgb(dodgerblue1_new[1], dodgerblue1_new[2], dodgerblue1_new[3], maxColorValue = 255)
tomato4_new <- col2rgb("tomato4")[,1]
tomato4_new <- rgb(tomato4_new[1], tomato4_new[2], tomato4_new[3], maxColorValue = 255)
tomato3_new <- col2rgb("tomato3")[,1]
tomato3_new <- rgb(tomato3_new[1], tomato3_new[2], tomato3_new[3], maxColorValue = 255)
tomato1_new <- col2rgb("tomato1")[,1]
tomato1_new <- rgb(tomato1_new[1], tomato1_new[2], tomato1_new[3], maxColorValue = 255)

htmltools::tagList(
      datatable(results_T2_table,
                caption = "Differential protein expression results in EV data at T2 from Giloteaux et al. 2024") %>%
      formatStyle("coef", "sig_cat", 
                  backgroundColor = styleEqual(c("Down in cases; FDR < 0.1", "Up in cases; FDR < 0.1",
                                                 "Down in cases; 0.3 < FDR < 0.1", "Up in cases; 0.3 < FDR < 0.1",
                                                 "Down in cases; 0.5 < FDR < 0.3", "Up in cases; 0.5 < FDR < 0.3"), 
                                               c(dodgerblue4_new, tomato4_new, 
                                                 dodgerblue3_new, tomato3_new,
                                                 dodgerblue1_new, tomato1_new)),
                  color = "white"))
}
```

#### Conclusions

Though we did not detect any differentially expressed proteins at FDR
< 10%, the suggestive signal for TNC at T0 is consistent with
Giloteaux *et al.* 2024. Their bootstrap approach to testing may
better accommodate the small sample size and actual data than the
asymptotic statistics used here.

#
